# Connectivity based on glucose dynamics reveals exaggerated sensorimotor network coupling on subject-level in Parkinson’s disease

**DOI:** 10.1007/s00259-024-06796-6

**Published:** 2024-06-17

**Authors:** Marina C. Ruppert-Junck, Vanessa Heinecke, Damiano Librizzi, Kenan Steidel, Maya Beckersjürgen, Frederik A. Verburg, Tino Schurrat, Markus Luster, Hans-Helge Müller, Lars Timmermann, Carsten Eggers, David Pedrosa

**Affiliations:** 1 Neurology Department at Medical Faculty Marburg, Philipps-University Marburg, Marburg, Germany; 2 Neurology Department, University Hospital of Marburg and Gießen, Baldingerstraße, 35043 Marburg, Germany; 3 Center for Mind, Brain and Behavior - CMBB, Universities Marburg and Gießen, Marburg, Germany; 4 Nuclear Medicine Department, Philipps-University Marburg, Marburg, Germany; 5 Department of Radiology and Nuclear Medicine, Erasmus University Medical Center, Rotterdam, The Netherlands; 6 Institute for Medical Bioinformatics and Biostatistics, Philipps-University Marburg, Marburg, Germany; 7 Knappschaftskrankenhaus Bottrop GmbH, Bottrop, Germany

**Keywords:** Glucose Dynamics, Dynamic [^18^F]-Fluorodeoxyglucose-PET, Network degeneration, Metabolic connectivity, Imaging biomarker, Multimodal connectome

## Abstract

**Purpose:**

While fMRI provides information on the temporal changes in blood oxygenation, 2- [18F]fluoro-2-deoxy-D-glucose ([^18^F]FDG)-PET has traditionally offered a static snapshot of brain glucose consumption. As a result, studies investigating metabolic brain networks as potential biomarkers for neurodegeneration have primarily been conducted at the group level. However, recent pioneering studies introduced time-resolved [^18^F]FDG-PET with constant infusion, which enables metabolic connectivity studies at the individual level.

**Methods:**

In the current study, this technique was employed to explore Parkinson’s disease (PD)-related alterations in individual metabolic connectivity, in comparison to inter-subject measures and hemodynamic connectivity. Fifteen PD patients and 14 healthy controls with comparable cognition underwent sequential resting-state dynamic PET with constant infusion and functional MRI. Intrinsic networks were identified by independent component analysis and interregional connectivity calculated for summed static PET images, PET time series and functional MRI.

**Results:**

Our findings revealed an intrinsic sensorimotor network in PD patients that has not been previously observed to this extent. In PD, a significantly higher number of connections in cortical motor areas was observed compared to elderly control subjects, as indicated by both static PET and functional MRI (*p*_Bonferroni−Holm_ = 0.027), as well as constant infusion PET and functional MRI connectomes (*p*_Bonferroni−Holm_ = 0.012). This intensified coupling was associated with disease severity (*ρ* = 0.56, *p* = 0.036).

**Conclusion:**

Metabolic connectivity, as revealed by both static and dynamic PET, provides unique information on metabolic network activity. Subject-level metabolic connectivity based on constant infusion PET may serve as a potential marker for the metabolic network signature in neurodegeneration.

**Supplementary Information:**

The online version contains supplementary material available at 10.1007/s00259-024-06796-6.

## Introduction

Metabolic imaging in neurodegenerative diseases has undergone a fundamental shift towards a network perspective [1]. In their influential study, Clark and Stoessl first described the covarying activity of homotopic brain regions [2]. Subsequently, metabolic equivalents of large-scale networks have been identified and patterns of interregional metabolic activity have been proposed as more sensitive for diagnosis than changes in local glucose uptake measured by 2 - [18F]fluoro-2-deoxy-D-glucose ([^18^F]FDG-) PET in neurodegenerative disorders [3, 4]. In Parkinson’s disease (PD), the second most prevalent neurodegenerative disorder characterised by motor symptoms, a typical covariance pattern involves increased activity in subcortical regions, decreased activity in the parietal and occipital cortex, and reduced metabolic covariance in frontal areas of the canonical resting-state networks [4–6]. However, the search for an individual-level applicable and interpretable biomarker for coherent metabolic network activity in PD remains unresolved.

The extent of coherent network activity detected by functional imaging is influenced by several factors. Specifically, functional MRI (fMRI) networks rely on the hemodynamic response – which reflects the close relationship between neural activity, blood flow and blood oxygen saturation [7]. On the other hand, [^18^F]FDG-PET measures glucose consumption [8]. Although both imaging techniques reflect closely related processes, the spatial convergence between hemodynamic and metabolic resting-state networks is only moderate [5, 9, 10] and typically quantified by the dice coefficient of similarity as an established quantitative measure for cross-modal correspondence in multimodal imaging studies [10, 11]. The moderate convergence has so far been attributed to fundamental differences in image acquisitions between traditional static [^18^F]FDG-PET protocols and fMRI time series [12]. In the former, only one static scan is acquired per subject, providing a snapshot of mean glucose consumption over the observation period [10]. Currently, metabolic covariance, which measures the interregional correlation of [^18^F]FDG uptake, is inaccessible for individual subjects. Instead, interregional correlations are typically expressed as group-level statistics, often referred to as metabolic connectivity [13].

Although widely used, detailed descriptions of the proximity of group-level metabolic connectivity to within-subject metabolic connectivity based on glucose dynamics are still in their earliest stages [14, 15]. Such descriptions were first mentioned in a ground breaking study exploring individual glucose dynamics in healthy young human [16, 17]. Analysing metabolic networks in neurodegeneration on a single-subject basis could improve our understanding of the networks involved and greatly advance our efforts to provide a network biomarker for ongoing neurodegeneration.

This study aimed to present dynamically acquired [^18^F]FDG-PET data from 15 PD patients and 14 healthy control subjects, for the first time, using the constant infusion functional PET (fPET) protocol. The objective was to identify metabolic patterns of the altered interregional communication on an individual level. Additionally, we intended to compare the obtained network to previously utilised methods, namely (1) sPET-based metabolic covariance or (2) hemodynamic network characteristics in PD, to identify their shared and distinct attributes. Participants underwent a multimodal imaging protocol, which included a resting-state fPET and fMRI acquisition. Spatial correspondence between intrinsic networks observed by all modalities and disease-related reconfigurations in PD were analysed using multimodal connectomes integrating neurobiological information from two modalities at both the whole brain level and in the identified motor network. The potential of subject-level metabolic connectivity derived by the dynamic fPET acquisition as disease biomarker was evaluated in relation to disease severity.

## Materials and methods

The study received approval from the local ethics committee of the medical faculty of the Philipps-University of Marburg (146/19). Additionally, authorisation for radiation exposure was obtained by the Federal Office for Radiation Protection. The study was conducted in adherence to the principles outlined in the Declaration of Helsinki. Prior to participation, all subjects provided written informed consent.

### Participants

A total of 15 patients with idiopathic Parkinson’s disease (PD) and 14 healthy control subjects were recruited for the study. Among those, 13 healthy controls and 12 PD patients underwent both imaging modalities. PD patients were recruited through the central study coordination of the Department of Neurology at the University Hospital of Marburg in Germany, whereas healthy control subjects were recruited through advertisements. Both groups had to meet the following inclusion criteria: age > 50 years and German language proficiency. Exclusion criteria included: safety concerns about MRI scanning, pregnancy, presence of neurological disorders other than PD, and blood glucose > 180 mg/dl at the time of PET examination. Additionally, patients were included if they met the following criteria: diagnosis of PD based on diagnostic criteria [18], Hoehn & Yahr stages 1-2.5, and clinically tolerable discontinuation of dopaminergic medication for up to three days. Exclusion criteria included: signs of dementia assessed by the Parkinson Neuropsychometric Dementia Assessment [19] (PANDA) screening tool.

### Clinical assessment

Motor severity was assessed according to part III of the Movement Disorder Society Unified Parkinson’s disease rating scale (MDS-UPDRS-III) in both the OFF- and ON-state [20]. Videotaping of motor performance enabled a blinded and independent assessment by another experienced movement disorder specialist. The levodopa equivalent daily dose (LEDD) for patients was determined using established criteria [21]. All participants underwent a cognitive test battery, which included the Montreal Cognitive Assessment (MoCa) among other tests.

### Neuroimaging

Participants underwent a multimodal resting-state imaging protocol, which involved the sequential use of dynamic [^18^F]FDG-PET scanning and MRI acquisition. In the case of individuals with PD, both scans were conducted while the participants were in the OFF-state of dopaminergic medication. The OFF-state duration for levodopa was 12 h, while for all dopamine agonists it was 72 h.

### Dynamic [^18^F]FDG-PET acquisition

Dynamic [^18^F]FDG-PET scans were acquired in the morning after overnight fasting and after measurements of blood sugar levels via AccuCheck. Participants were positioned in a supine position in a SIEMENS Biograph 6 (Siemens, Germany) with dimmed light and eyes open. On average, 199.3 ± 5.27 MBq [^18^F]FDG was infused continuously using a perfusor (Braun, Germany) at a rate of 0.01 ml/s. Participants were instructed to lay as still as possible and do not think of anything in particular. Following a low-dose CT transmission scan, PET data were acquired in list-mode for 90 min and saved for offline reconstruction. Resulting events were corrected for attenuation and sinograms were reconstructed into 90 1-minute frames with a matrix size of 168 × 168 (voxel size: 4.07 × 4.07 × 1.5 mm) using the 3D Ordered Subset Expectation Maximization (OSEM) algorithm implemented in the software Syngo (Siemens). Reconstructed frames were smoothed with a 5 mm Gaussian kernel, exported as DICOM files and subsequently converted into 4D NifTi file per subject using the dicom2niix tool in MRICroGL (RRID: SCR_024413) [22].

### fMRI acquisition

MRI scanning was performed on a Trio Tim Syngo 3 Tesla MR-scanner (Siemens, Erlangen) at the Department for Psychiatry and Psychotherapy of the University Hospital of Marburg, Germany. Participants were positioned in the scanner and underwent structural MRI with the following parameters: repetition time (TR): 1900 ms, echo time (TE): 2.52 ms, voxel size: 1.0 × 1.0 × 1.0 mm^3^. For the subsequent fMRI measurement, subjects were instructed to keep their eyes opened and to avoid thinking about anything in particular. The eye area was checked by camera throughout the measurement. The 8-minute lasting multiband echo-planar imaging sequence [23–27] was characterised by the following parameters: 490 time points, TR 1040 ms, TE 30.0 ms, 3 × 3 × 3 mm^3^ voxel size and 48 slices. DICOM files were converted into NifTi files using the dicom2niix tool in MRICroGL (RRID: SCR_024413) [22].

### fMRI and FDG-fPET preprocessing and analysis

Preprocessing of MRI scans was performed by using the default preprocessing pipeline implemented in the Conn toolbox v. 21a (RRID: SCR_009550) in batch mode (code available on GitHub repository) and SPM12 (RRID: SCR_007037) except for slice time correction. In brief, functional and structural scans were coregistered and centered. Realignment and unwarping were applied to functional volumes. Functional and structural scans were directly normalized into Montreal Neurological Institute (MNI) standard space and segmented into white matter, grey matter and cerebrospinal fluid (CSF) using the SPM12 unified segmentation and normalisation procedure [28]. The last 30 frames were retained from the dynamic PET data due to low signal intensity of initial frames. Remaining dynamic [^18^F]FDG-PET scans were centered and reoriented if necessary and motion correction was applied to individual frames using affine transformation as implemented in SPM12. The resulting mean motion corrected [^18^F]FDG-PET scans were spatially normalised into MNI space using the “Old Segment” approach in SPM12 with reference to a modality-specific template from healthy elderly subjects [29]. Each modality’s spatially normalised scans were fed into group-level independent component analyses (ICA) using the GIFT toolbox (Translational Research in Neuroimaging & Data Science, https://trendscenter.org/software/gift/,(RRID:SCR_024416)) and intensity-normalised with reference to the global mean. Metabolic time series of all subjects per group were concatenated for spatial ICA. The number of components was set to 10 for all analyses. Resulting components were thresholded at z = 1.5. For the sensorimotor component, the threshold was set to 1 to enable a visualisation of all cortical clusters. To demonstrate consistency with previous PET studies, an exploratory SPM single-subject analysis of hyper/hypometabolism at subject level in comparison to control subjects was also performed and is reported in the extended data.

### ROI-based extraction of glucose dynamics

For interregional connectivity analyses, the data were parcellated based on automated anatomical labeling (AAL) atlas (RRID: SCR_003550) with 116 regions for whole brain analyses in all modalities. For within network connectivity analyses, the derived motor component was binarised and parcellated based on AALv3 to resolve the contribution of small subcortical nuclei. Signal time courses for individual regions of interest (ROIs) were extracted for each subject from dynamic [^18^F]FDG-PET data using the ROI toolbox MarsBaR (RRID: SCR_009605) [30] and intensity-normalised by division by the time frame’s global mean per subject. Interregional connectivity measures were obtained by calculating the Pearson correlation coefficient between all pairs of regions. In detail, group-level sPET based connectivity was calculated via correlating each region’s summed uptake across all frames with all other regions summed uptake per group. fPET-based or fMRI-based connectivity was calculated via correlating each region’s metabolic (30 frames) or hemodynamic time series (490 frames) with all other region’s time series per subject. For group level figures, the mean connectivity per connection was visualised and p-values based on one sample *t*-tests were used to threshold significant connections. Adjacency matrices per group and modality were derived via binarizing the connectivity matrices with only significant connections retained. Binary adjacency matrices were used to create connectome plots using BrainNet Viewer (RRID: SCR_009446) [31]. Cross-modal correspondence was examined by evaluating common edges of the adjacency matrix obtained by each pair of modalities by calculating the Sörensen dice index of similarity: $DC= \frac{2 \,|M1 \cap M2|}{\left|M1\right|+\left|M2\right|}$ with M1 and M2 representing the adjacency matrix of one modality [14]. The DC is an established quantitative measure that is required and standard practice in the field of multimodal imaging. It was also applied to allow drawing conclusions on the comparability with the only other existing fPET data set in healthy subjects [11]. The primary objective for applying a quantitative measure was to investigate whether fPET-based connectivity patterns, which are derived from metabolic time series and methodologically closer to fMRI, exhibit higher comparability to fMRI-based connectivity patterns than connectivity based on sPET. The relation between each connection’s connectivity strength was analysed with linear regression analysis and calculating the Pearson correlation coefficient between both modalities’ connectivity values across all regions. Nodal degree was calculated as the number of significant connections per ROI in the multimodal matrices and between-group differences in regions representing a lobule or functional unit (e.g., cortical motor) were calculated using Student’s *t* test or Mann-Whitney U test and considered significant when *p* < 0.05 after Bonferroni-Holm correction. In an exploratory analysis, we conducted first correlation analyses between subject-level fPET metabolic connectivity or nodal degree, defined as the number of significant connections per ROI on subject level within the identified motor network, and disease severity, quantified by UPDRS-III off, using Spearman correlation.

### Statistical analysis

Statistical analyses were performed on demographic, behavioural and clinical data using R (RRID: SCR_001905) [32]. Group comparisons were conducted using either Student’s *t* test or Mann-Whitney U test based on the results of the Shapiro-Wilk test of normality. The R codes created for the analysis of the data set can be accessed through a Github repository (https://github.com/ruppertm/RSfPET-fMRI.git).

## Results

### Cohort specification

A total of 12 mild to moderately affected PD patients (62.92 ± 9.48 years, three females) and 13 healthy controls (59.54 ± 5.13 years, six females) provided multimodal data for this study (refer to Table 1 for clinical and demographic information). Cognitive screening tests did not reveal differences in cognitive function between the groups (PANDA: *W* = 83, *P* = 0.81; MoCA: *W* = 92.5, *P* = 0.60). Furthermore, both groups exhibited similar levels of depressive mood (BDI-II, *t* = 0.11, *P* = 0.91). The average MDS-UPDRS-III score for PD patients without medication was 32.42 ± 10.46 indicating mild to moderate severity of overall motor symptoms. All patients, except for one, received dopaminergic medication, with an average levodopa equivalent daily dose (LEDD) of 583.15 ± 300.23 mg.

**Table 1:** Demographic, clinical and behavioural data

Variable	Healthy controls (*n* = 13)	PD patients (*n* = 12)	statistics	*P*-value
Sex male / female (n)	7 / 6	9 / 3	*OR* = 0.40	0.41
Age	59.54 ± 5.13	62.92 ± 9.48	*T* = −1.10	0.29
*Behavioural characteristics*				
PANDA	24.58 ± 4.56	23.67 ± 6.44	*W* = 83.00	0.81
BDI-II	7.62 ± 6.97	7.33 ± 6.08	*T* = 0.11	0.91
BIS-11	55.67 ± 6.64	60.50 ± 10.86	*W* = 58.00	0.29
*Clinical characteristics*				
Disease duration (y)	–	6.18 ± 3.82	–	–
MDS-UPDRS-III	–	32.42 ± 10.46	–	–
LEDD (mg)	–	583.15 ± 300.23	–	–
Side of onset L / R / None (n)	–	5 / 3 / 4	–	–

*Abbreviations* BDI-II = Beck’s Depression Inventory II, BIS-11 = Barret Impulsiveness Scale-11, HC = Healthy control, LEDD = Levodopa equivalent daily dose, MDS-UPDRS-III – Movement Disorder Society Unified Parkinson’s disease Rating scale, part III, OR = Odds ratio, PD = Parkinson’s disease, PANDA = Parkinson Neuropsychometric Dementia Assessment

### Intrinsic metabolic motor network with subcortical contribution is exclusively detectable in patients

**Fig. 1:**
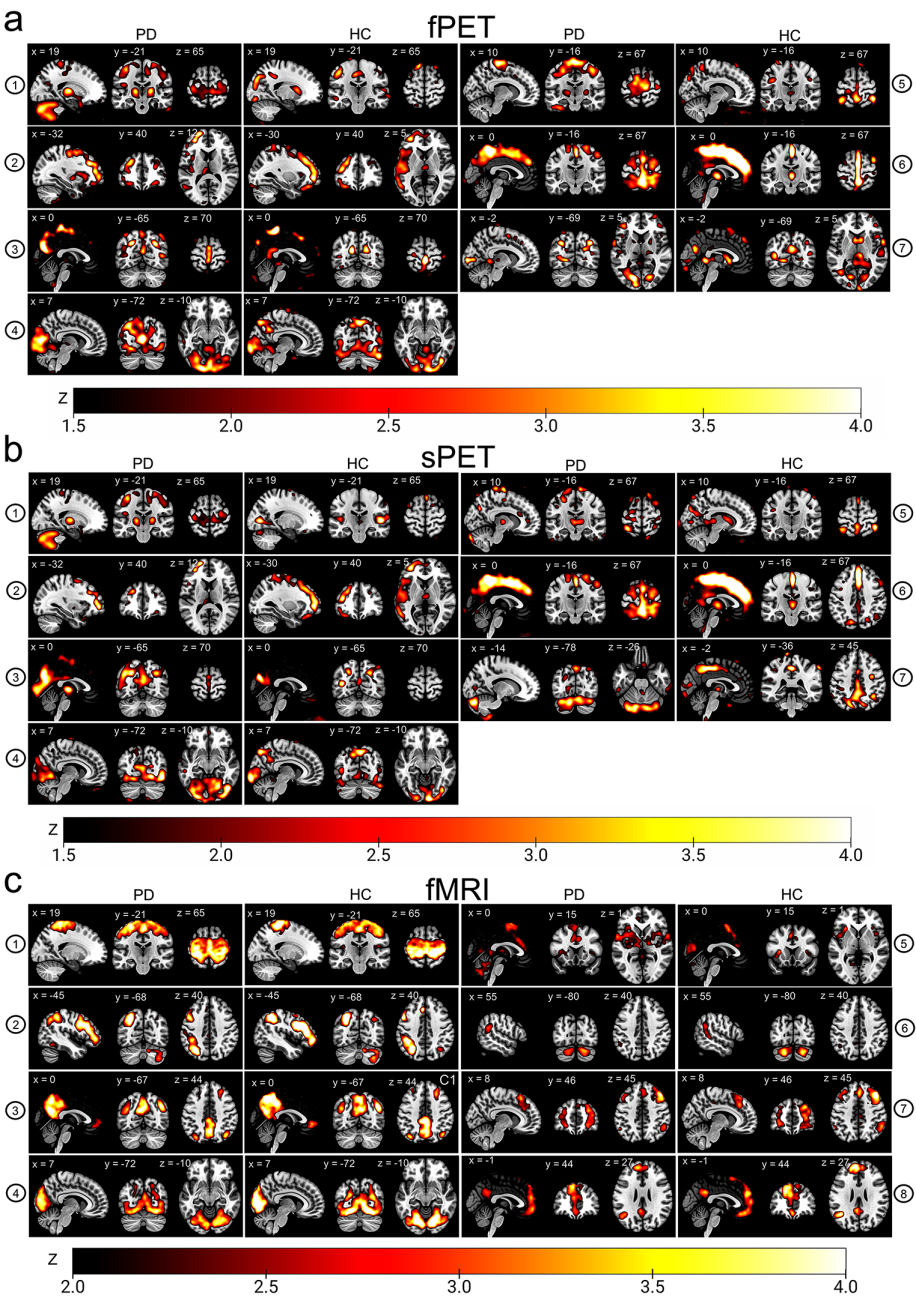
Group-level intrinsic resting-state networks derived by summed [^18^F]FDG-PET scans, dynamic [^18^F]FDG-PET time series and fMRI time series ICA-derived independent components per group derived by (**a**) fPET [^18^F]FDG uptake time series (dynamic constant infusion), (**b**) [^18^F]FDG uptake summed across the acquisition time (static), (**c**) fMRI time series. Components are thresholded at z = 1.5 or z = 2 respectively. Color bars indicate z-values

The data-driven decomposition through modality-specific ICAs revealed distinct intrinsic connectivity networks across all three modalities. Within the PET modality, one of the identified components encompassed the sensorimotor network, with clear representation of subcortical regions, including bilateral putamina, a bilateral thalamic cluster, left midbrain, the cerebellum, and clusters distinctly localised in the medial bilateral pre- and postcentral gyri in PD patients (Fig. 1a, component 1). A similar component was detected in healthy control subjects, but it was more confined to subcortical areas and lacked cerebellar clusters, and a complete covering of the sensorimotor cortex (Fig. 1a, component 1). In contrast, a component including only the motor cortex was traceable in fMRI in both groups (Fig. 1c, component 1). The PET-based sensorimotor components were comparable in patients and showed only a slightly wider distribution in the sPET technique (Fig. 1a-b, component 1). Interestingly, an exploratory analysis of hyper-/hypometabolism at the whole-brain level for the individual patient showed pronounced hypermetabolism in the motor cortex in many patients, which overlapped with the identified motor network (extended data).

Other identifiable networks are outlined below. Using the PET techniques, components spanning the left mid temporal gyrus, left inferior parietal gyrus, angular gyrus, and the caudate nucleus were present in both groups (Fig. 1a, component 2). Similar network structures were identified in a combination of fMRI components (Fig. 1c, component 5 and 7). Another PET component included the precuneus and bilateral inferior parietal clusters and the bilateral caudate nuclei, medial thalamus (Fig. 1a, component 3). In the fMRI modality, a comparable component included the precuneus and the prefrontal cortex in both groups, outlining a typical default-mode-network-like structure (Fig. 1c, component 3). A primary visual component extending into the extrastriate visual cortex and spanning the mid occipital gyrus and bilateral calcarine gyri was detected in all three modalities (Fig. 1a–c, component 4). An additional motor component was exclusively found in fPET in PD patients, spanning the motor cortex and the bilateral supplementary motor area (Fig. 1a, component 5). Unique fMRI components were a fronto-temporal salience network (Fig. 1c, component 5), a fronto-temporal language network and a cerebellar network (Fig. 1c, component 2 and 6). Three unique components were detected in the PET techniques, consisting of an occipital component in the fPET (Fig. 1a, component 7), a cerebellar component (Fig. 1b, component 7), and a cingular component per group in the sPET and fPET (Fig. 1a-b, component 6).

### Multimodal connectomes imply exaggerated sensorimotor coupling in PD

**Fig. 2:**
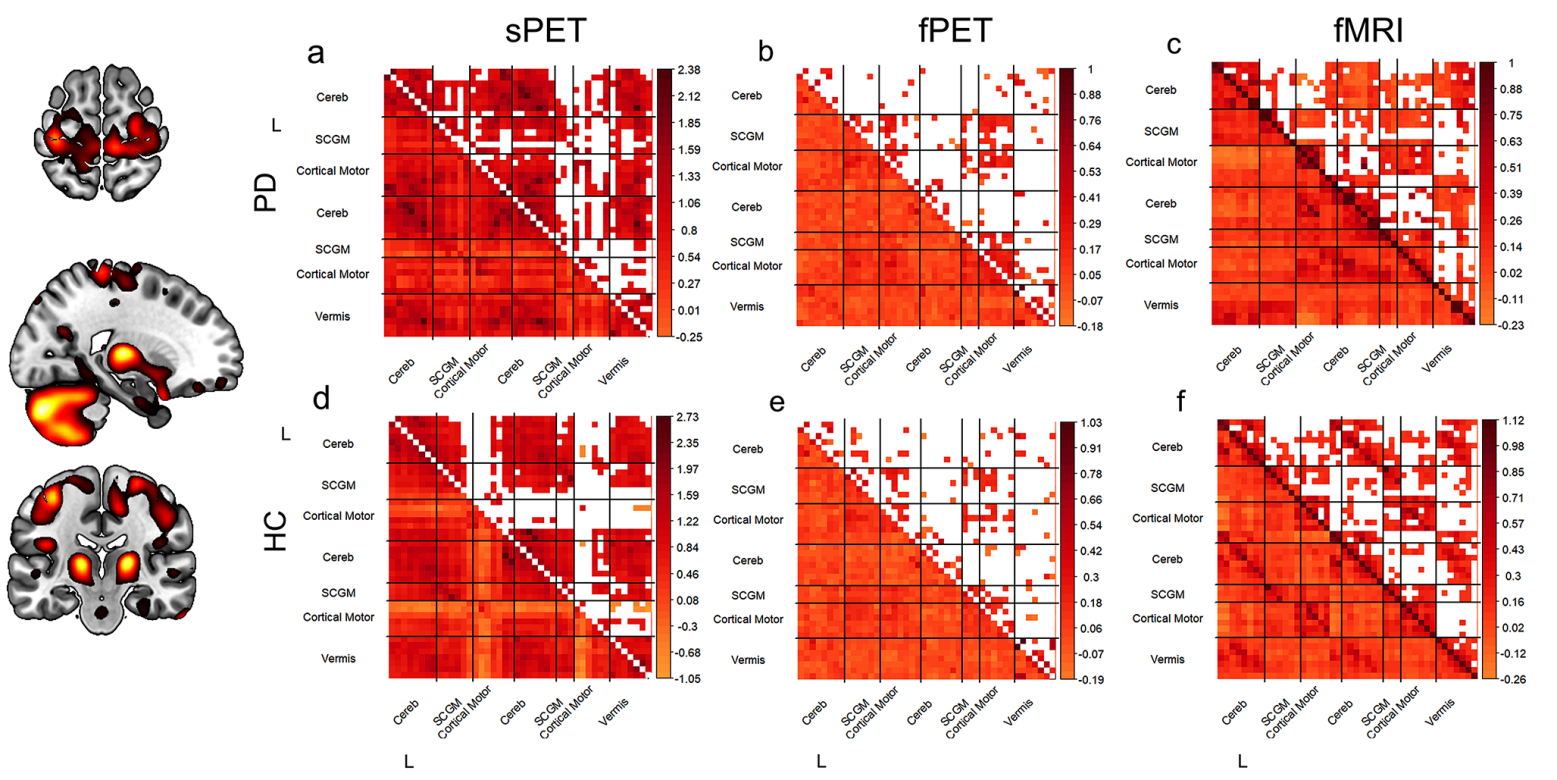
Group-level sensorimotor network connectivity based on summed [^18^F]FDG-PET scans, dynamic [^18^F]FDG-PET time series and fMRI time series Sensorimotor network mean Pearson’s r correlation coefficient matrices calculated per group derived by (**a**) [^18^F]FDG uptake summed across the acquisition time (static), (**b**) [^18^F]FDG uptake time series (dynamic constant infusion), (**c**) fMRI time series from data-driven ROIs obtained by parcellating fPET PD sensorimotor network with AALv3 atlas regions. For static data, connectivity was calculated on group level across subjects. For time series data, connectivity measures were calculated on subject level, standardised and group-mean was visualised. Correlation coefficients with p values < 0.05 are blank in upper diagonal. sPET/fPET (PD: *n* = 14, HC: *n* = 13), fMRI (PD: *n* = 13, HC: *n* = 13). Color bars indicate Fisher-transformed correlation coefficients (z)

To examine connectivity within the identified sensorimotor network, a multimodal connectome approach was used to assess differences in connectivity between the participating regions across all modalities. In line with the network identification findings, PD patients displayed robust connectivity between bilateral paracentral lobules, postcentral lobules, and the supplementary motor area with the rest of the network, whereas healthy controls showed little to no connectivity with the other cortical network regions (Fig. 2a, d). These between-group differences were more or less evident in all modalities (Fig. 2a-f).

**Fig. 3:**
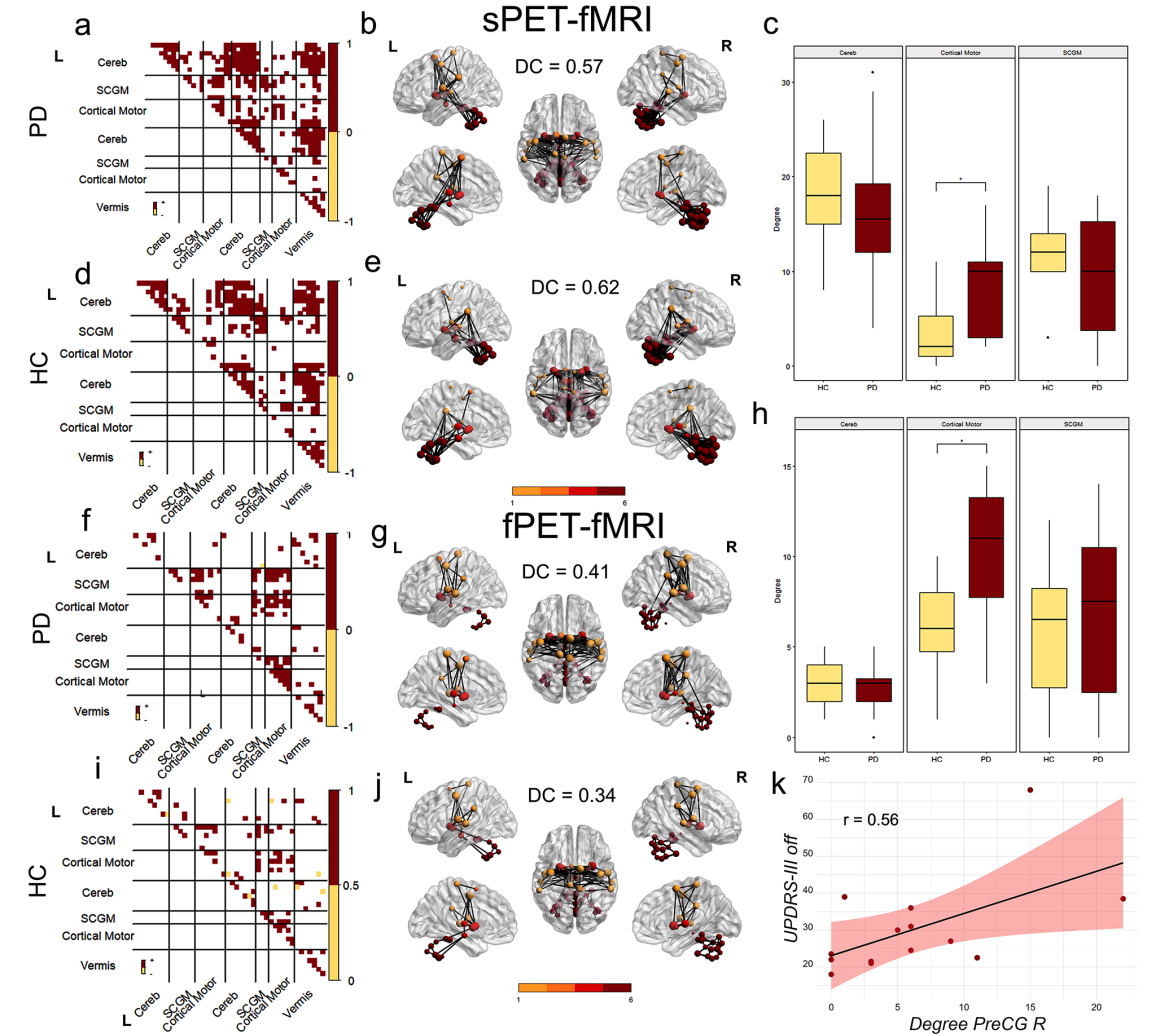
Sensorimotor group-level multimodal connectomes and nodal degree based on sPET-fMRI and fPET-fMRI A, (**d**) Tertiary group-mean matrices of sPET-fMRI- and (**f**, **i**) fPET-fMRI-based significant common connections within the identified sensorimotor network. Red color indicates positive connections; yellow color indicates negative connections, (**b**, **e**, **g**, **j**) dice coefficients refer to multimodal connectivity matrices per group of multimodal connectomes with reference to positive connections. Nodal size is proportional to nodal degree. Group comparison of nodal degree per lobule/functional unit derived by multimodal connectomes based on two-sample t-tests (**c**, **h**). Positive correlation between motor severity and fPET-based subject-level nodal degree in the precentral gyrus of the identified network (**k**). * *p* < 0.05, **p < 0.01, ***p < 0.001 after Bonferroni-Holm correction

When comparing nodal degree in multimodal connectomes derived from either two modalities, an increased degree was observed for cortical motor regions in PD patients compared to healthy controls in both the sPET-fMRI (*t* = −2.89, p-value after Bonferroni-Holm (BH) correction pBH = 0.027) and fPET-fMRI connectomes (*t* = − 3.26, pBH = 0.012). Importantly, subject-level metabolic fPET connectivity (ρ = 0.63, *p* = 0.019, extended data Fig. 1) and nodal degree (ρ = 0.56, *p* = 0.036, Fig. 3k) were significantly associated with motor severity, as quantified by UPDRS-III off, in the right precentral gyrus of the identified motor network. A trend for a similar positive association was observed for functional connectivity along the same connection, but not significant (ρ = 0.36, *p* = 0.230). Similarly, no significant association was found for nodal degree based on functional connectivity with UPDRS-III in the regions of the motor network.

Both the fMRI and sPET-based connectomes exhibited high within-network connectivity between ipsi- and contralateral cerebellar regions in both groups, while this was not observed in the fPET modality (Fig. 2a-f). The connectivity pattern of subcortical motor regions demonstrated a higher level of agreement between fPET and fMRI (Fig. 3f, i), whereas the vermis’ and cerebellar connections corresponded more closely between sPET and fMRI in both groups (Fig. 3a, d). The quantification of the correspondence of the connectomes indicated higher values in both groups for sPET vs. fMRI compared to the fPET vs. fMRI (DC PD: 0.57, DC HC: 0.62) (Fig. 3b, e, g and j) and fPET vs. sPET connectomes (extended data Fig. 2).

### PD-related changes on whole-brain level

**Fig. 4:**
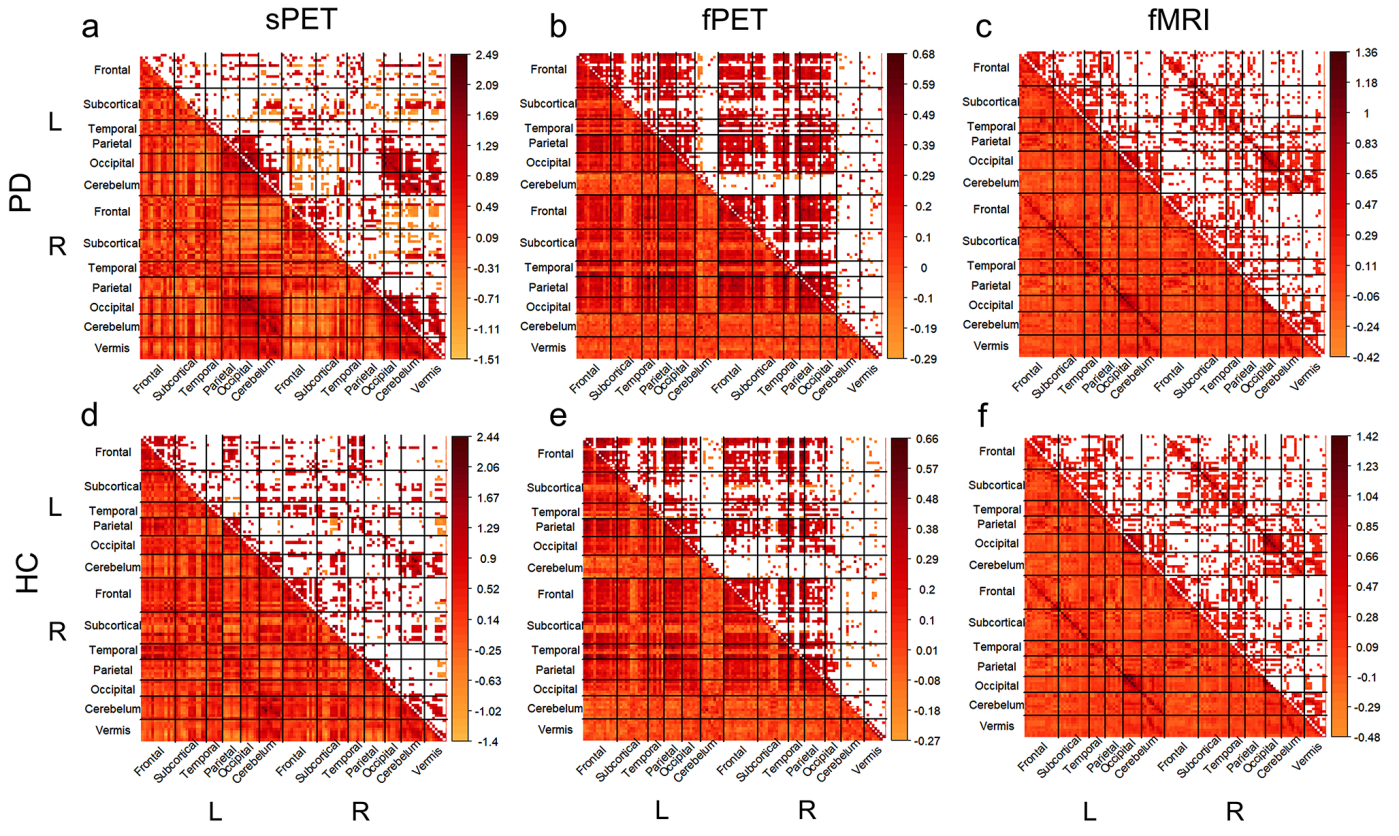
Whole brain group-level connectivity based on summed [^18^F]FDG-PET scans, dynamic [^18^F]FDG-PET time series and fMRI time series Whole-brain mean Pearson’s r correlation coefficient matrices calculated per group derived by (**a**) [^18^F]FDG uptake summed across the acquisition time (static), (**b**) [^18^F]FDG uptake time series (dynamic constant infusion), (**c**) fMRI time series from automated anatomical labeling (AAL) atlas ROIs. For static data, connectivity was calculated on group level across subjects. For time series data, connectivity measures were calculated on subject level, standardized and group-mean was visualised. Correlation coefficients with p values < 0.05 are blank in upper diagonal. sPET/fPET (PD: *n* = 14, HC: *n* = 13), fMRI (PD: *n* = 13, HC: *n* = 13). Color bars indicate Fisher-transformed correlation coefficients (z)

The whole brain connectomes showed significant common edges between ipsilateral frontal, parietal, and occipital regions in both groups across all modalities (Fig. 4a-f). In both sPET and fMRI connectomes, both groups exhibited significant connections between ipsi- and contralateral cerebellar regions. However, the fPET connectome generally displayed few connections in the cerebellum and was more shaped by interhemispheric connections in both groups (Fig. 4b and e).

**Fig. 5:**
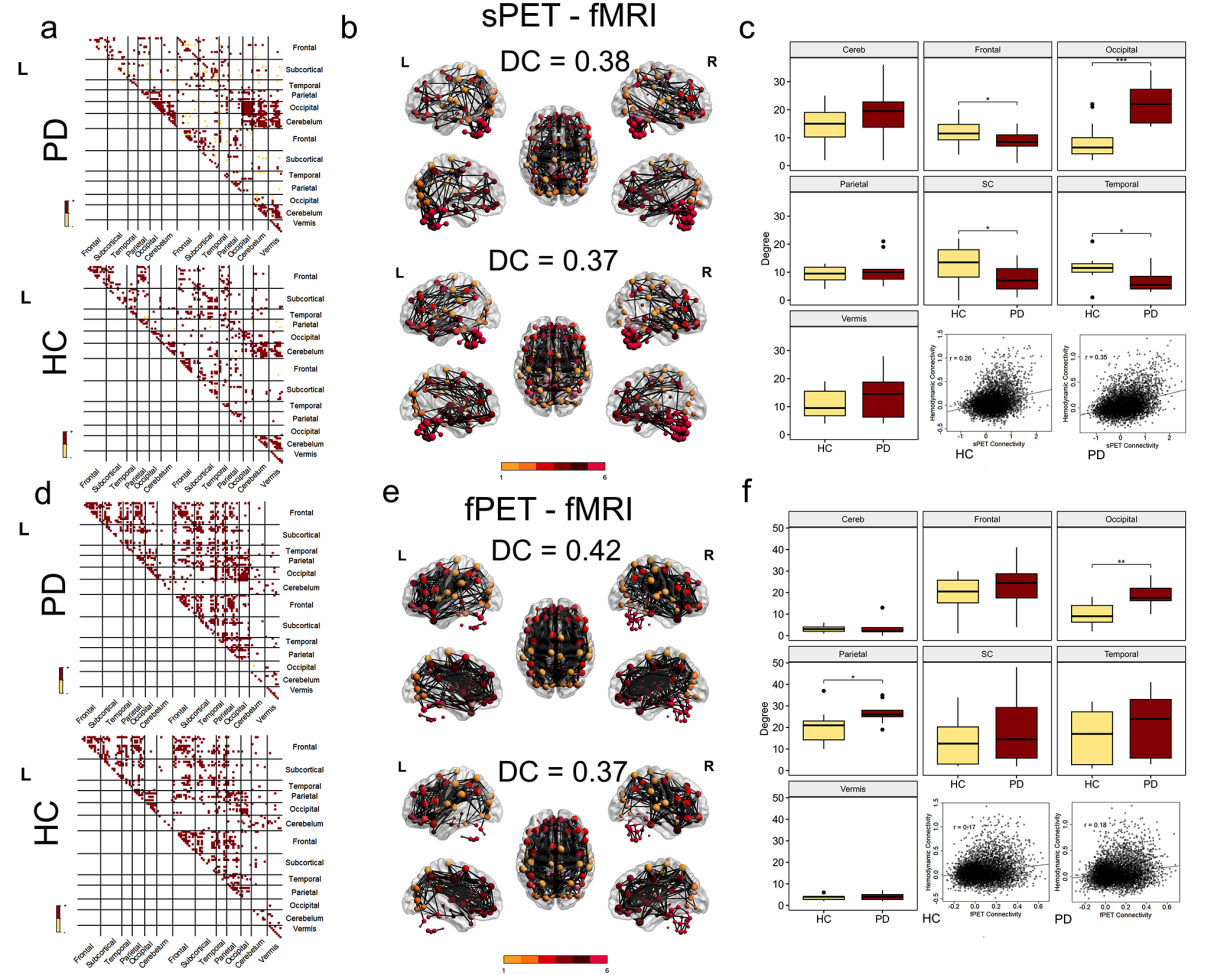
Whole brain group-level multimodal connectomes and nodal degree based on sPET-fMRI and fPET-fMRI connectomes. (**a**, **d**) Tertiary group-mean matrices of sPET-fMRI and fPET-fMRI of significant common connections. Red color indicates positive connections; yellow color indicates negative connections, (**b**, **e**) dice coefficients refer to multimodal connectivity matrices per group. Nodal size is proportional to nodal degree. Nodal degree per lobule/functional unit per group (**c**, **f**). * *p* < 0.05, **p < 0.01, ***p < 0.001 after Bonferroni-Holm correction

The comparison of adjacency matrices from either two modalities revealed common edges primarily in ipsilateral frontal, cerebellar, and occipital regions in both groups. The fPET vs. fMRI connectomes indicated a higher number of connections to homotopic contralateral regions, such as frontal, subcortical, temporal, parietal and occipital regions in comparison to sPET vs. fMRT connectomes (Fig. 5a, d).

However, across modalities, occipital regions showed a higher number of connections in the patients’ group. The between-group comparison of nodal degree derived from the sPET-fMRI connectome revealed a significantly higher degree for occipital regions (*t* = −5.23, p_BH)_ < 0.001) and significantly reduced degree for subcortical regions (*t* = 2.77, p_BH_ = 0.021, refer to Fig. 5c), frontal regions (*t* = 2.79, p_BH_ = 0.021), and temporal regions in PD (*t* = 2.63, p_BH_ = 0.027). The nodal degree calculated from the fPET-fMRI multimodal connectome also revealed a significantly increased nodal degree for occipital areas in PD patients (*t* = −4.40, p_BH_ = 0.001, Fig. 5f) and an increase in parietal connections (*t* = −2.89, p_BH_ = 0.03, Fig. 5f). The increased nodal degree of occipital regions was also observed in the corresponding sPET-fPET connectomes (*t* = −9.51, p_BH_ < 0.0001, see extended data Fig. 3).

In terms of inter-modality correspondence, the fPET vs. fMRI connectomes indicated the highest similarity between-modalities in both groups (PD: 0.42, healthy controls: 0.37, Fig. 5e). The DC values for the comparison of sPET vs. fMRI connectomes were in a similar range (Fig. 5b, e). The association in connectivity strength between the modalities was higher in the comparison of sPET vs. fMRI than in fPET vs. fMRI and moderate for both groups (Fig. 5c, f). The DC for comparing sPET and fPET were found to be fair for both groups and association in connectivity strength was fair for the patients and moderate for the healthy subjects (extended data Fig. 3).

## Discussion

Investigating the metabolic basis of resting-state neural networks using a combination of [^18^F]FDG-PET and fMRI is an area of research that has received limited attention. Some studies have combined both techniques to explore their complementary strengths [9]. However, the applicability and interpretability of these studies, particularly in the context of network biomarkers for neurodegeneration, have been constrained by the inability to derive metabolic connectivity at the individual level. The development of a time-resolved [^18^F]FDG-PET (fPET) protocol opens new possibilities by enabling a direct comparison of connectivity based on hemodynamics and metabolic dynamics. In this study, we applied the fPET protocol in a clinical context to identify subjects’ PD-related changes in metabolic connectivity based on dynamic [^18^F]FDG-fPET. We also investigated the relationship of these changes to group-level sPET and hemodynamic network properties, allowing for an integrative analysis of multimodal connectomes, as recently requested [12]. Our results can be summarized as follows: First, dynamic PET acquisitions with a constant infusion provide more precise metabolic network structures, including deeper brain structures and a subcortical-cortical motor network in PD. Secondly, sPET was able to approximate similar circuit changes in the sensorimotor network, that were detectable at the individual level with fPET and - most importantly - correlated with motor severity. Finally, multimodal connectomes integrating neurobiological information from static or dynamic PET and fMRI identified whole-brain level reconfigurations in PD, as well as specific changes in the identified motor network. These findings suggest an exaggerated coupling of the motor cortex in PD from a metabolic perspective and raises the possibility of using this measure of a metabolic network signature as a potential biomarker.

Our study’s components provide a comprehensive understanding, particularly in the context of motor networks, compared to larger cohorts that use concatenated static scans [10]. This implies that the constant infusion protocol itself may enable more precise identification of networks and higher subcortical resolution, which are essential prerequisites for identifying non-motor symptoms of the disease, for example. Generally, the PET-based components exhibited a greater involvement of subcortical areas compared to the fMRI components and displayed more differences between groups, particularly in motor regions. Although the number of participants was limited due to regulatory restrictions, our data demonstrate that analyses of smaller groups using sPET based on mean images of the fPET acquisition, yield valid information on network expansion in the group. This is supported by the significant overlap between components based on static and dynamic PET data and similar findings regarding hypermetabolism in PD. The fact that subcortical regions were generally more recognisable in the PET techniques can be attributed to the effects of attenuation correction, which ensures uniform resolution of structures throughout the entire brain^11^. Although there was some correspondence between established resting-state networks and fMRI, as observed in previous sPET studies (e.g., visual component) [9, 10], other components displayed unique expression in PET. The fPET modality detected equivalents of all metabolic patterns identified by sPET, but the components appeared less noisy and with fewer small clusters. This is plausible given the increased number of frames included in the fPET analysis. Notably, a fPET-based sensorimotor component showed a remarkable spatial extent with clearly defined functional boundaries and subcortical contribution. The observed motor network demonstrates a high spatial similarity to a stereotypic PD metabolic pattern described by Eidelberg et al. based on sPET which is typically characterised by increased activity in pallido-thalamic and primary motor areas and decreased activity in parieto-occipital areas [33]. This is further supported by the fact that this network was more distinct in patients compared to controls. Cerebellar components were also previously described, but were distinguishable from other motor areas in fMRI and sPET [10]. The cingular components correspond well with a similar component described in Di et al. [9] and could reflect venous trapping of [^18^F]FDG. Additionally, this component was not found in previous fMRI studies [10].

Our multimodal whole brain connectome analyses revealed a moderate association between metabolic connectivity using sPET and fPET, and functional connectivity using fMRI. It is important to acknowledge that, despite recent optimisations, fPET still has a lower temporal resolution compared to fMRI [34]. However, it is unlikely that the slower dynamics of neurometabolic coupling can be more accurately captured within seconds. Our findings suggest that sPET connectivity is primarily driven by connections between homotopic regions, while fPET demonstrates more connections between different brain lobes and a stronger correspondence with fMRI at the whole brain level [34]. Interestingly, we did not find any evidence of cerebellar fPET connectivity, which has not been previously analysed [34]. We have demonstrated that the networks derived from both techniques bear a closer resemblance to fMRI connectomes than to other PET-based connectomes. Consistent with the findings of Sala et al., we observed a reconfiguration in the multimodal sPET-fMRI connectome, characterised by a decrease in frontal connections in PD [6], accompanied by a decline in subcortical and temporal connectivity, and an increase in occipital connectivity.

In the sensorimotor network, both group-level sPET-based connectivity and fPET-based connectivity suggest an increased coupling of cortical motor regions in PD. Previous studies have demonstrated increased glucose consumption and exaggerated coherence in cortical motor areas in PD, particularly at the beta frequency (13–30 Hz). This aberrant synchronisation is mitigated by dopaminergic therapy and Deep Brain Stimulation [35]. The pronounced connectivity detected by both sPET and fPET may indicate a metabolic correlate of increased oscillatory activity in cortical motor areas. Supporting this idea and highlighting the clinical significance of our results, heightened activity was found to be associated with motor severity, as demonstrated by regression analyses between subject-level metabolic connectivity and UPDRS part III scores in the OFF state. Importantly, our group observed a component indicative of subcortical basal ganglia hypersynchronisation in an independent sPET sample of 51 PD patients vs. 16 control subjects using a seed-based approach and ICA-based decomposition in a previous study (extended data). The question of whether increased oscillations in basal ganglia cortical networks drive the pathophysiology or compensate for basal ganglia dysfunction is still a topic of debate [36]. Longitudinal analyses of the presented approach, which could also be applicable in the prodromal stage of PD [37], may address this unresolved question. The focus on a mild to moderate disease stage off medication in the current pilot study is a limitation, that allows our results to be attributed only to this type of patients. However, we focused on the mild to moderate stage of the disease to investigate the profound metabolic changes in the full-blown disease, before completing our observations with the changes in the early stages without initial levodopa medication. The inclusion criteria, limiting our sample to mildly and moderately affected patients, were chosen to maintain a degree of variability in motor severity enabling clinical correlations.

Recent research has suggested that measures of the chemical component of synaptic transmission, as reflected in molecular imaging, are equally meaningful and underexplored indicators of neural communication. These measures may offer untapped potential due to the prevalence of chemical synapses in the human brain [12]. Our results indicate that both PET measures are meaningful connectivity measures [11] and each provide distinct information that is not captured by fMRI [12]. The fact that our data reveal characteristic network-level changes in PD, as demonstrated by three different techniques that capture different aspects of brain connectomics, underscores how connectome studies integrating different techniques may enhance our understanding of the fundamental principles of brain connectomes. Another important insight is that multimodal connectomes (sPET-fMRI and fPET-fMRI) can identify changes in network topology in PD that are likely to be more robust, as they are supported by two independent measures [11] of brain connectivity. These findings are of interest for future simultaneous applications on hybrid MR-PET systems [38] and could also open new perspectives for other diseases of the nervous system, as the technique enables investigation of the relationship between individual disease severity and subject-level metabolic network signature, including connectivity and topological network measures.

In this study, we systematically evaluated a new technique that aims at comprehensively assessing metabolic dynamics between different regions at the individual level. Our method reliably measured network activity specific to PD. In conclusion, our approach allows us to identify a sensorimotor network at a high subcortical resolution but with broad spatial coverage in PD, as well as measure interregional coupling within the cortical motor network for each individual. Our results highlight the disease-relevant coupling of the motor cortex in PD from a metabolic perspective, using direct measurement of neural dynamics. These findings provide intriguing insights into intrinsic neural networks and their potential applications in MR-PET hybrid systems, as well as advancements in biological staging models.

## Electronic supplementary material

Below is the link to the electronic supplementary material.

Supplementary Material 1

## Data Availability

The data sets generated during and/or analysed during current study are available from the corresponding author on reasonable request.

## Code availability

The code generated to analyse the data set is publicly available on our github repository https://github.com/ruppertm/RSfPET-fMRI.git.
