# Supplementary material for: Connectivity based on glucose dynamics reveals exaggerated sensorimotor network coupling on subject-level in Parkinson’s disease
Source: Eur J Nucl Med Mol Imaging. 2024 Jun 17;51(12):3630–42. doi: 10.1007/s00259-024-06796-6 (PMC11445336; doi:10.1007/s00259-024-06796-6)
Supplement: Supplementary file 1 — Supplementary Material 1 [file 259_2024_6796_MOESM1_ESM.docx]

**Connectivity based on glucose dynamics reveals exaggerated sensorimotor network coupling on subject-level in Parkinson’s disease**

Marina C. Ruppert-Junck*^1,2,3^, Vanessa Heinecke^1^, Damiano Librizzi^4^, Kenan Steidel^1,2^, Maya Beckersjürgen^1^, Frederik A. Verburg^4,5^, Tino Schurrat^4^, Markus Luster^4^, Hans-Helge Müller^6^, Lars Timmermann^1,2,3^, Carsten Eggers^1,2,7^, David Pedrosa^1,2,3^

**Author affiliations:**

1 Neurology Department at Medical Faculty Marburg, Philipps-University Marburg, Germany

2 Neurology Department, University Hospital of Marburg and Gießen GmbH, Germany

3 Center for Mind, Brain and Behavior - CMBB, Universities Marburg and Gießen, Germany

4 Nuclear Medicine Department, Philipps-University Marburg, Germany

5 Department of Radiology and Nuclear Medicine, Erasmus University Medical Center, Rotterdam, the Netherlands

6 Institute for Medical Bioinformatics and Biostatistics, Philipps-University Marburg, Germany

7 Knappschaftskrankenhaus Bottrop GmbH, Bottrop, Germany


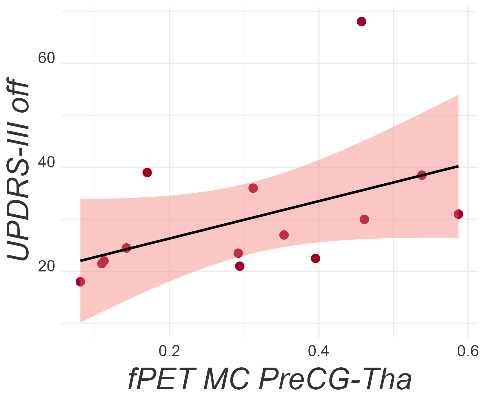


**Supplementary Fig. 1 Correlation between subject-level metabolic connectivity and disease severity in PD patients** The applied fPET technique enabled correlation analysis between subject-level metabolic connectivity and clinical measures. Motor severity measured by Unified PD rating scale III (UPDRS-III) correlated with fPET connectivity between the left thalamus and right precentral gyrus (ρ = 0.63, p = 0.019).

**
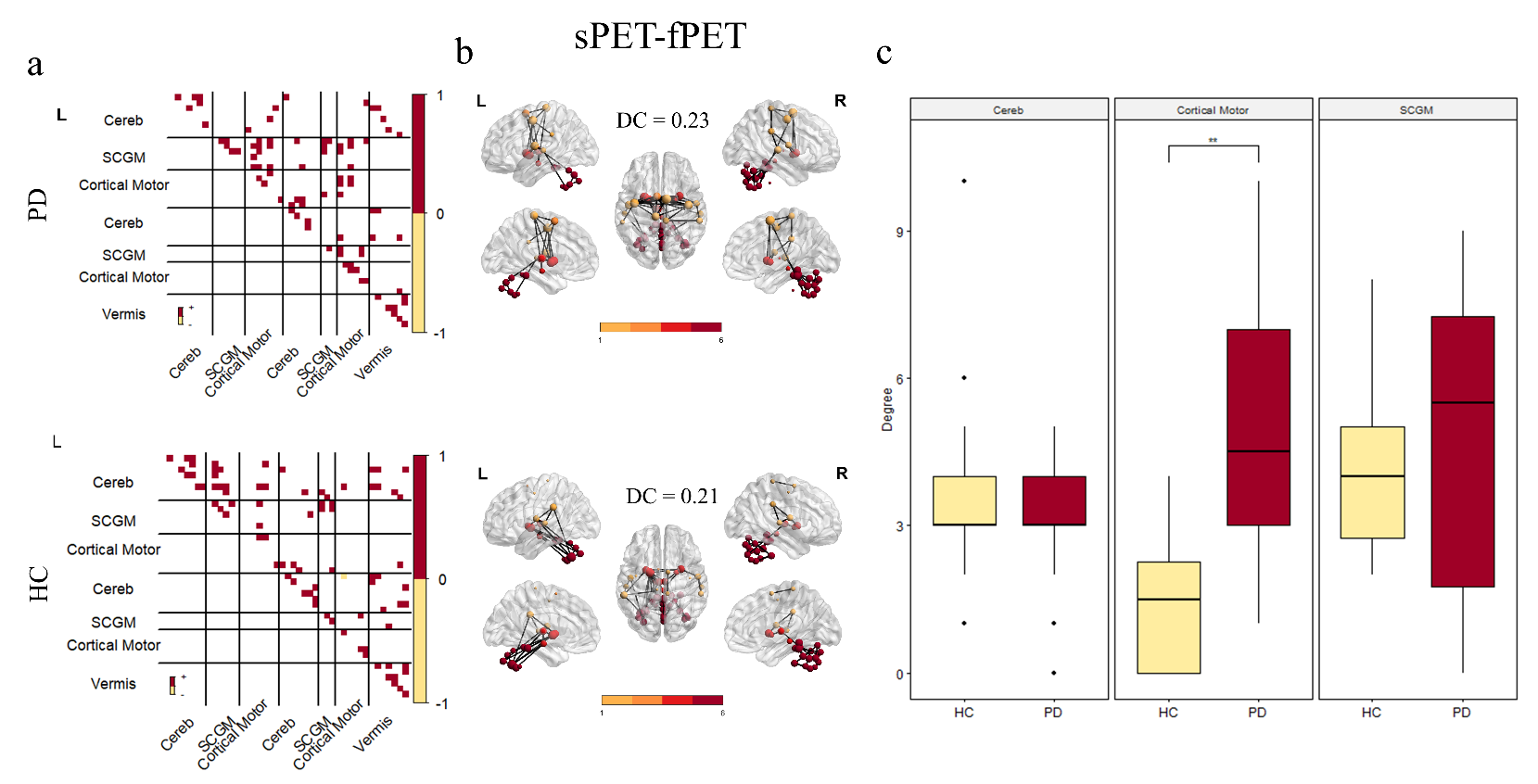
**

**Supplementary Fig. 2** **Sensorimotor group-level multimodal connectomes and nodal degree based on sPET-fPET connectomes** a) Tertiary group-mean matrices of sPET-fPET-based significant common connections within the identified sensorimotor network. Red color indicates positive connections; yellow color indicates negative connections, b) dice coefficients refer to multimodal connectivity matrices per group of multimodal connectomes with reference to positive connections. Nodal size is proportional to nodal degree. Nodal degree per lobule/functional unit per group (c). * p < 0.05, **p < 0.01 after Bonferroni-Holm correction.


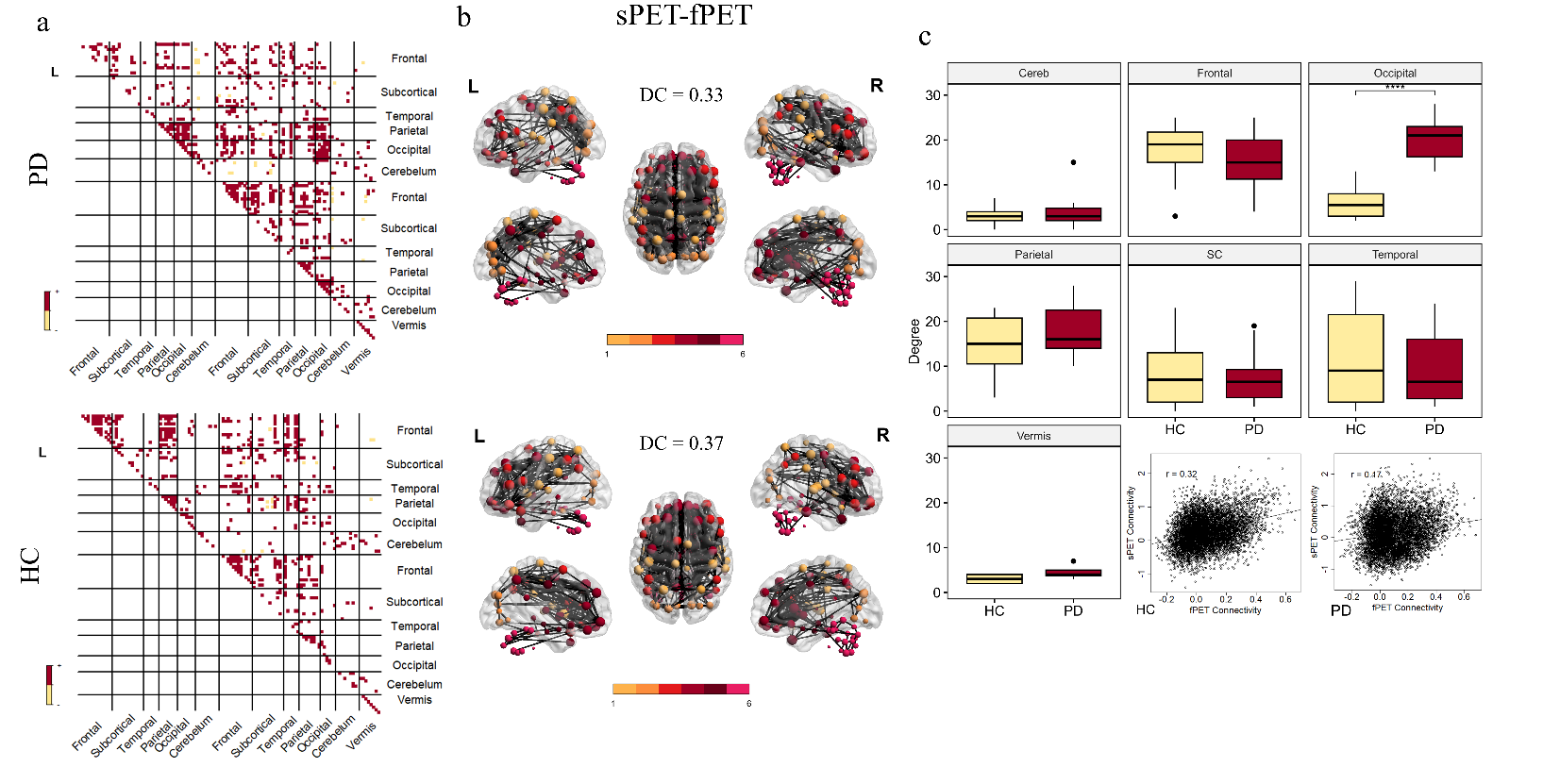


**Supplementary Fig. 3 Whole brain group-level multimodal connectomes and nodal degree based on sPET-fPET connectomes** a) Tertiary group-mean matrices of sPET-fPET with significant common connections. Red color indicates positive connections; yellow color indicates negative connections, **b)** dice coefficients refer to multimodal connectivity matrices per group. Nodal size is proportional to nodal degree. Nodal degree per lobule/functional unit per group (c). * p < 0.05, **p < 0.01, ***p < 0.001, ****p < 0.0001 after Bonferroni-Holm correction.


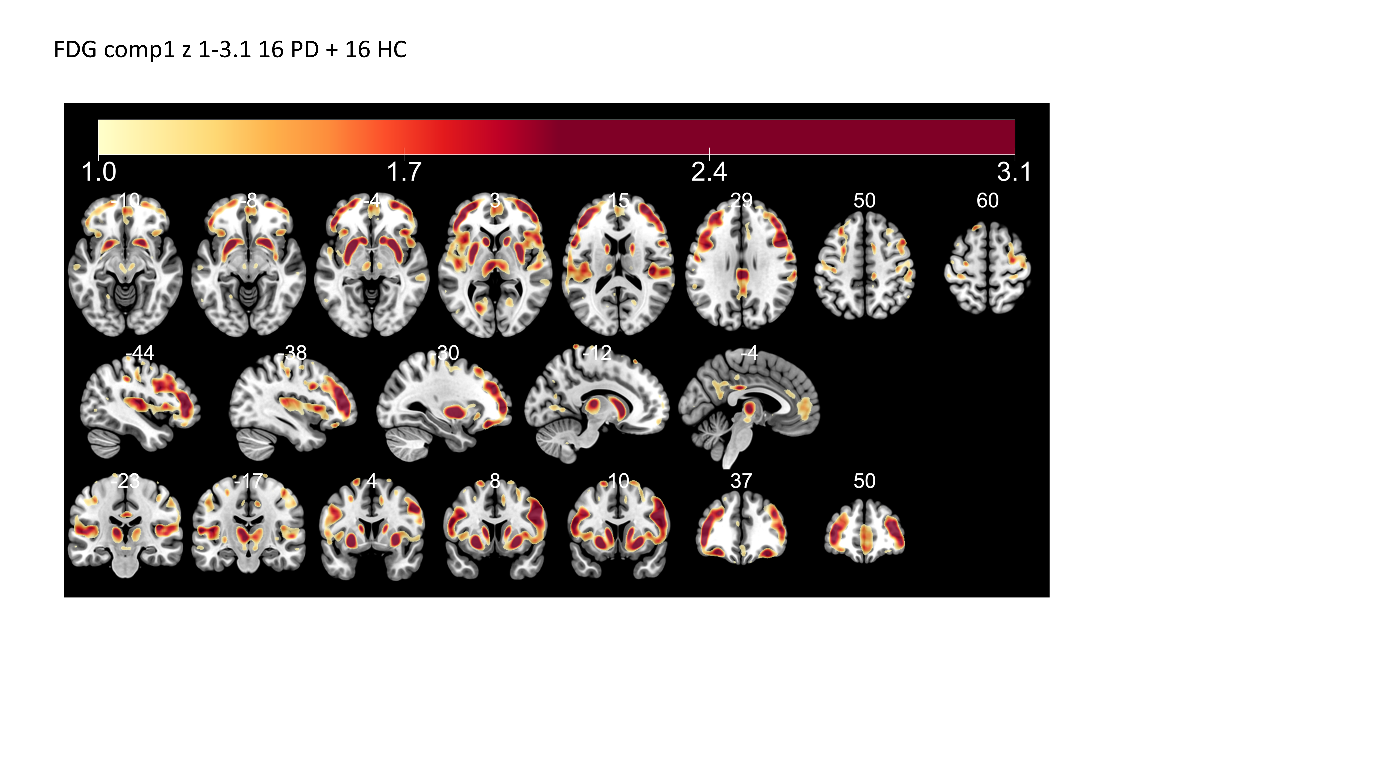


**Supplementary Fig. 4** **Sensorimotor component with subcortical contribution based on an independent sPET sample of PD patients (n=16) and healthy controls (n=16)** ICA-derived independent component per group derived by concatenated FDG sPET data (static). Component is thresholded at z = 1.

PD1 mean vs HC, PD>HC

A


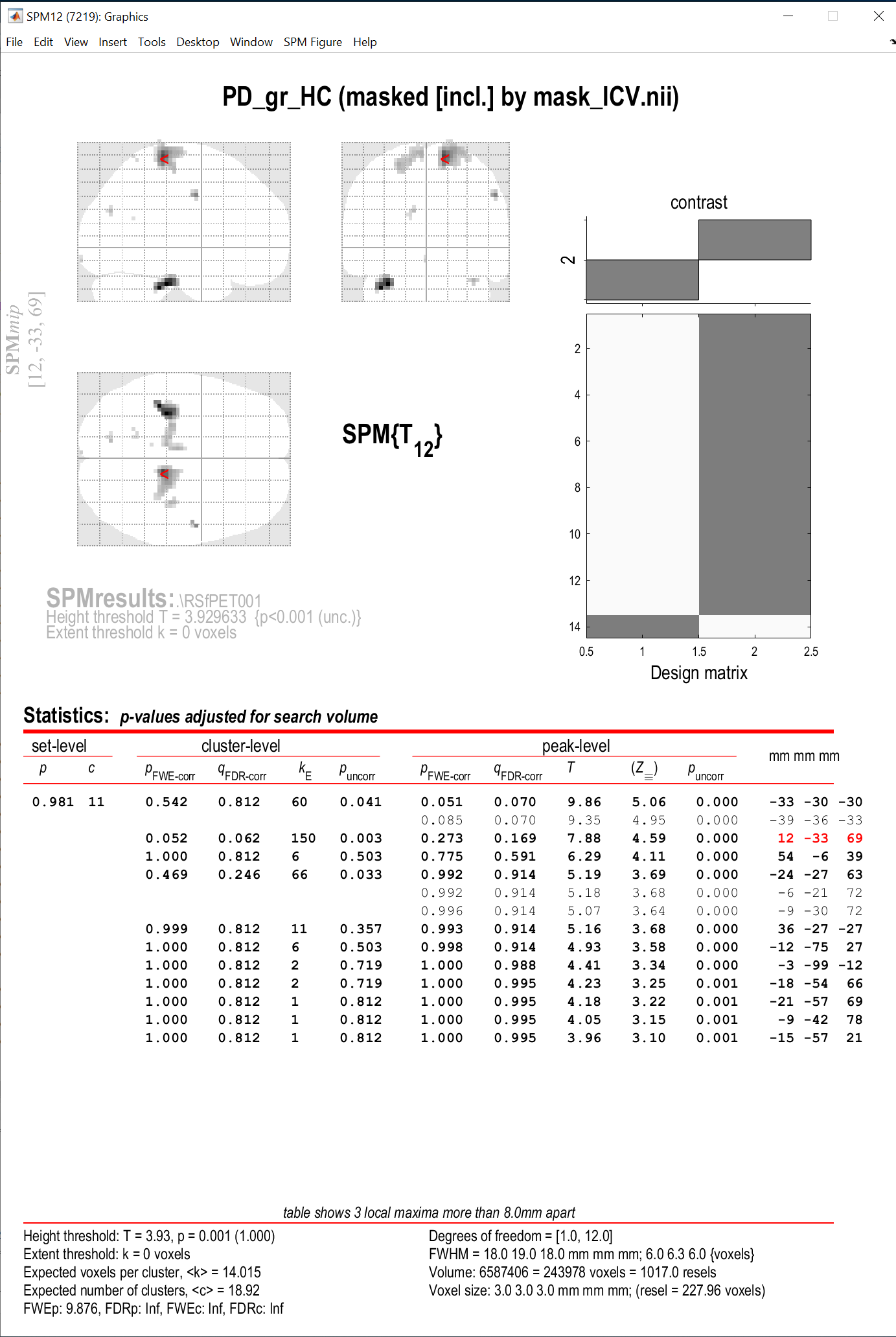


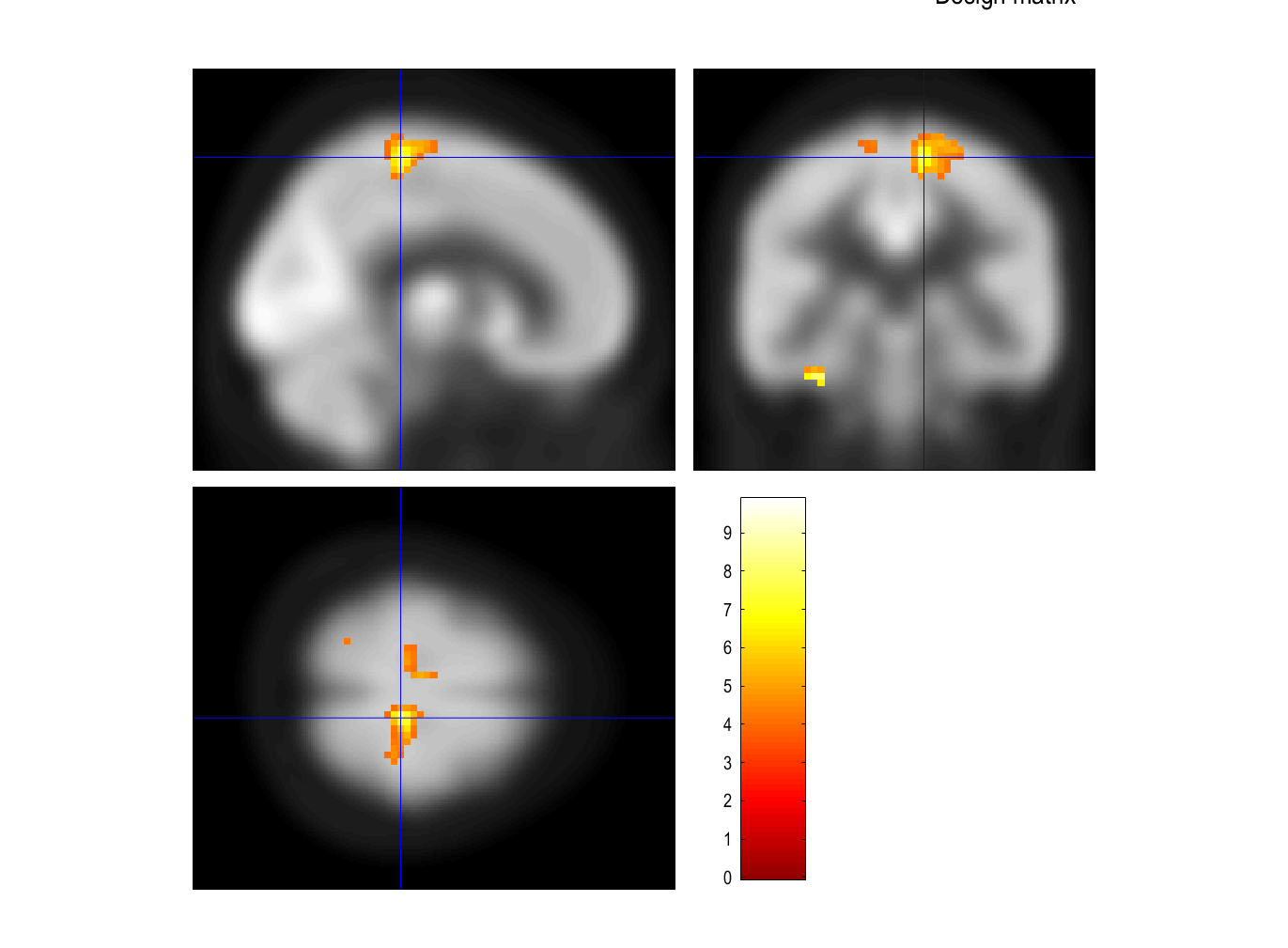


B

**Supplementary Fig. 5 Areas with relative hypermetabolism in patient 1 in comparison to healthy controls** Single subject SPM analysis of mean 18F-FDG-PET scan (min 61-90) from subject one, which was voxel-wise compared to corresponding mean scans from the healthy control sample (T = 3.93, p < 0.001 uncorrected).

PD2 mean vs HC, PD<HC


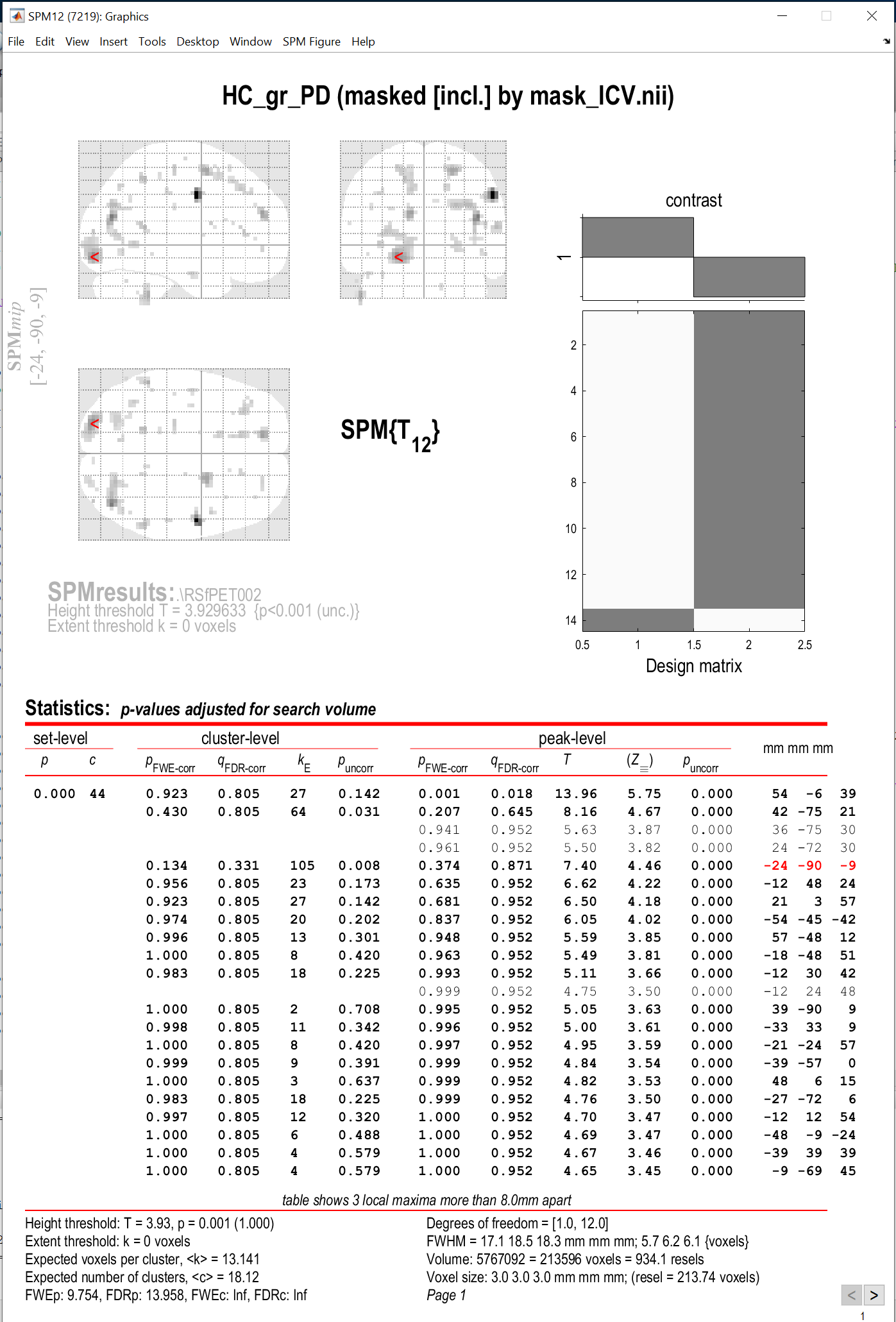


A


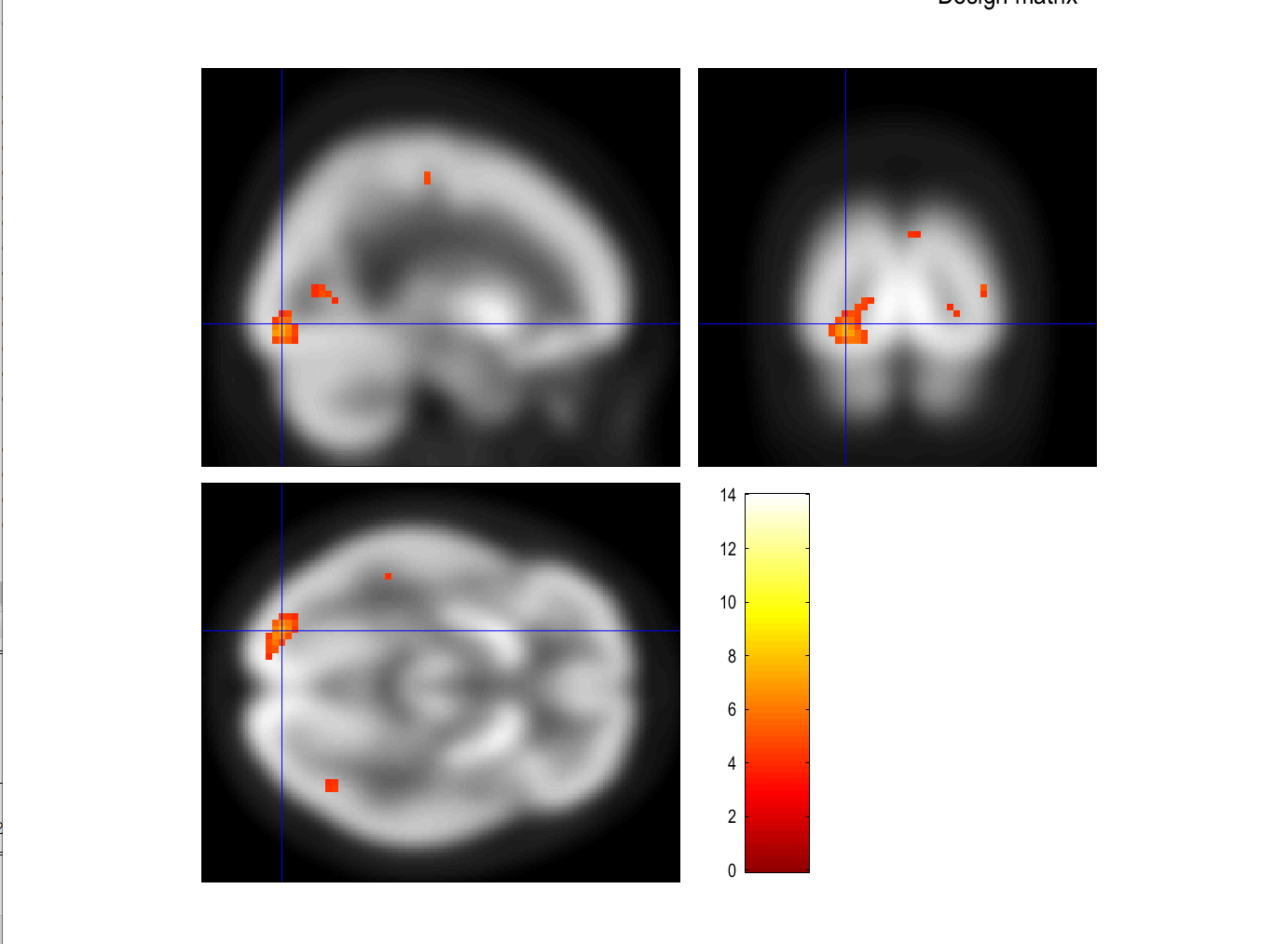


B

**Supplementary Fig. 6 Areas with relative hypometabolism in patient 1 in comparison to healthy controls** Single subject SPM analysis of mean 18F-FDG-PET scan (min 61-90) from subject one, which was voxel-wise compared to corresponding mean scans from the healthy control sample (T = 3.93, p < 0.001 uncorrected).

PD2 mean vs HC, PD>HC


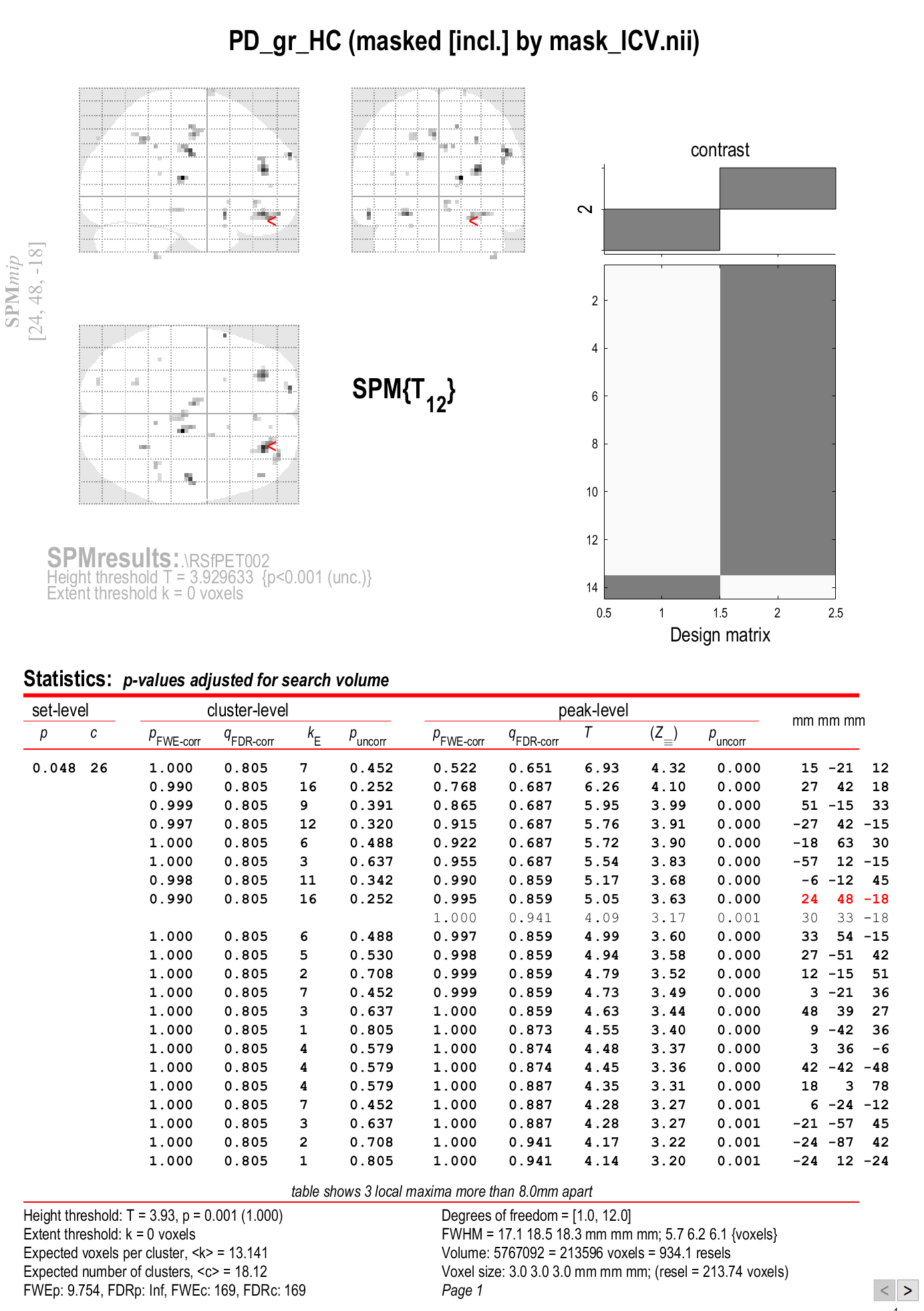


A


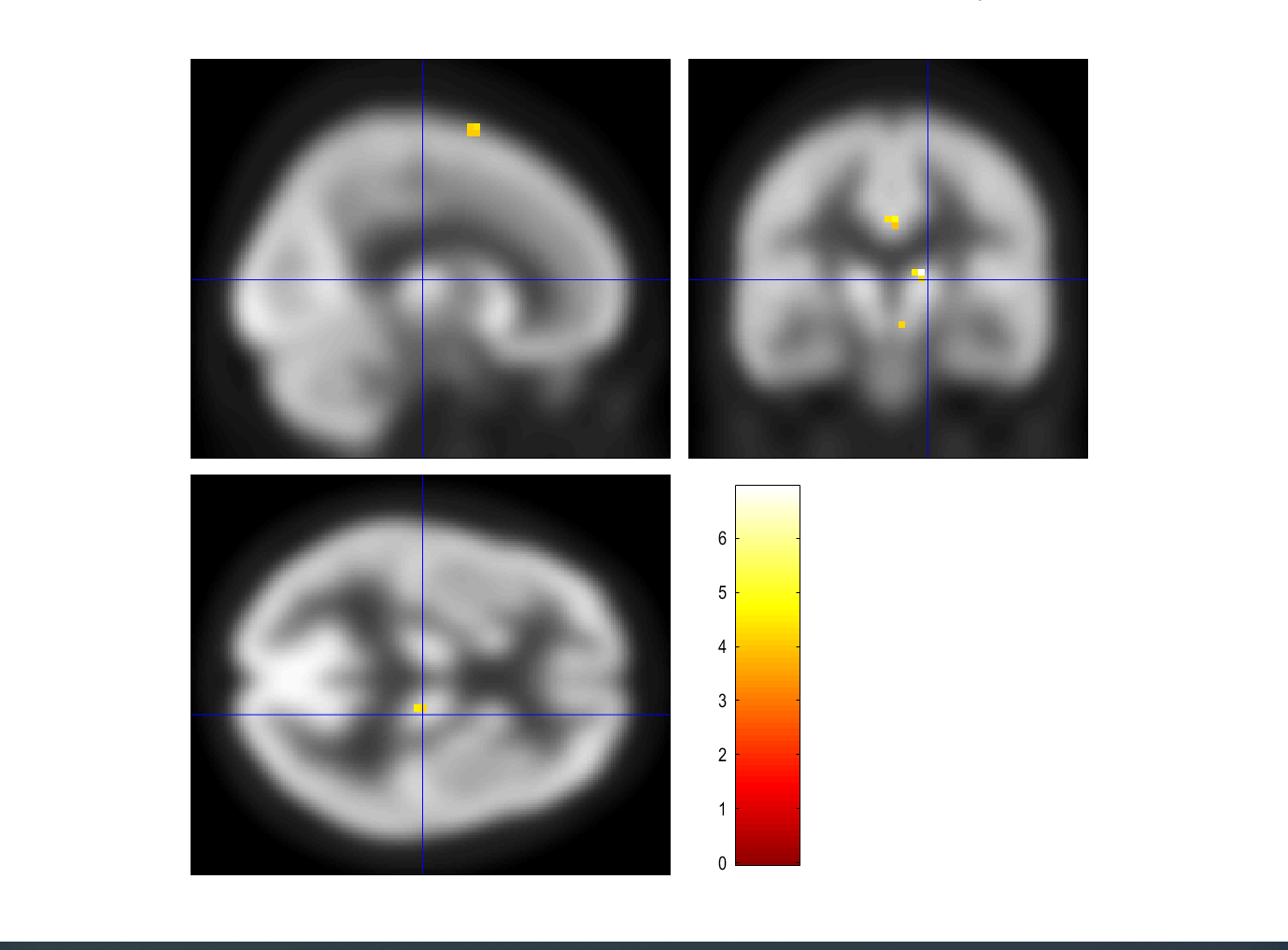


B

**Supplementary Fig. 7 Areas with relative hypermetabolism in patient 2 in comparison to healthy controls** Single subject SPM analysis of mean 18F-FDG-PET scan (min 61-90) from subject one, which was voxel-wise compared to corresponding mean scans from the healthy control sample (T = 3.93, p < 0.001 uncorrected).

PD3 mean vs HC, PD>HC


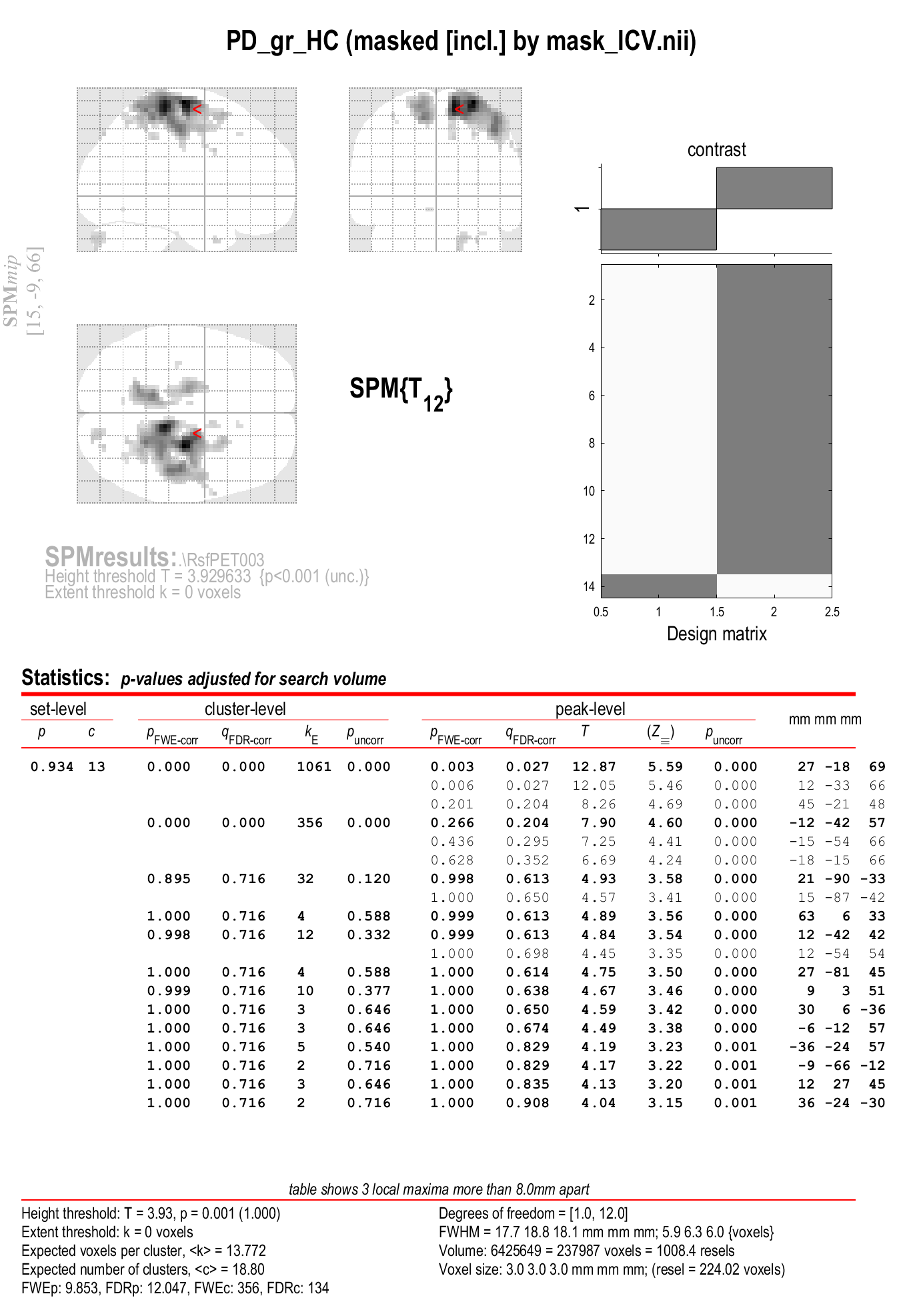


A


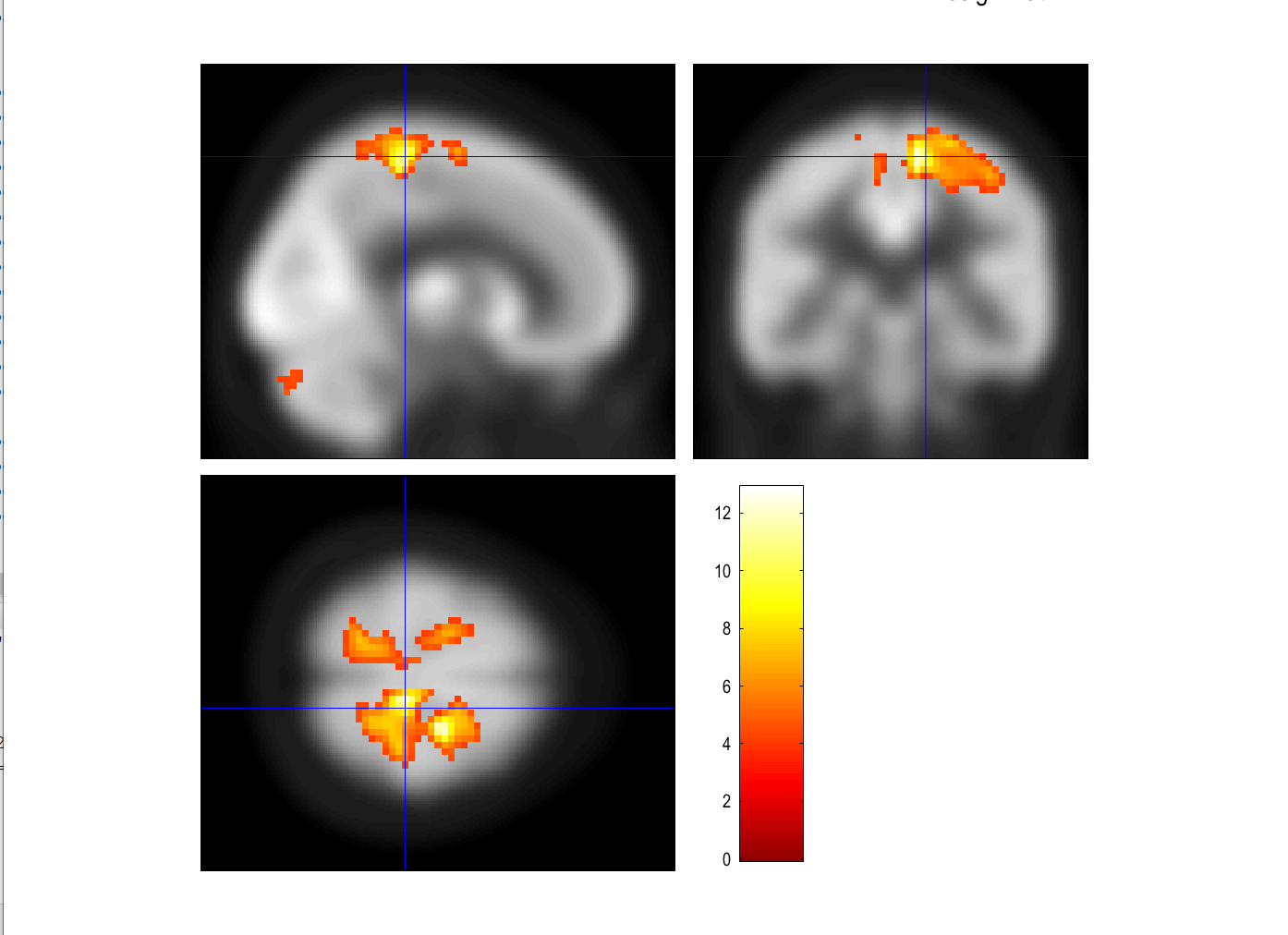


B

**Supplementary Fig. 8 Areas with relative hypermetabolism in patient 3 in comparison to healthy controls** Single subject SPM analysis of mean 18F-FDG-PET scan (min 61-90) from subject one, which was voxel-wise compared to corresponding mean scans from the healthy control sample (T = 3.93, p < 0.001 uncorrected).

PD3 mean vs HC, PD<HC


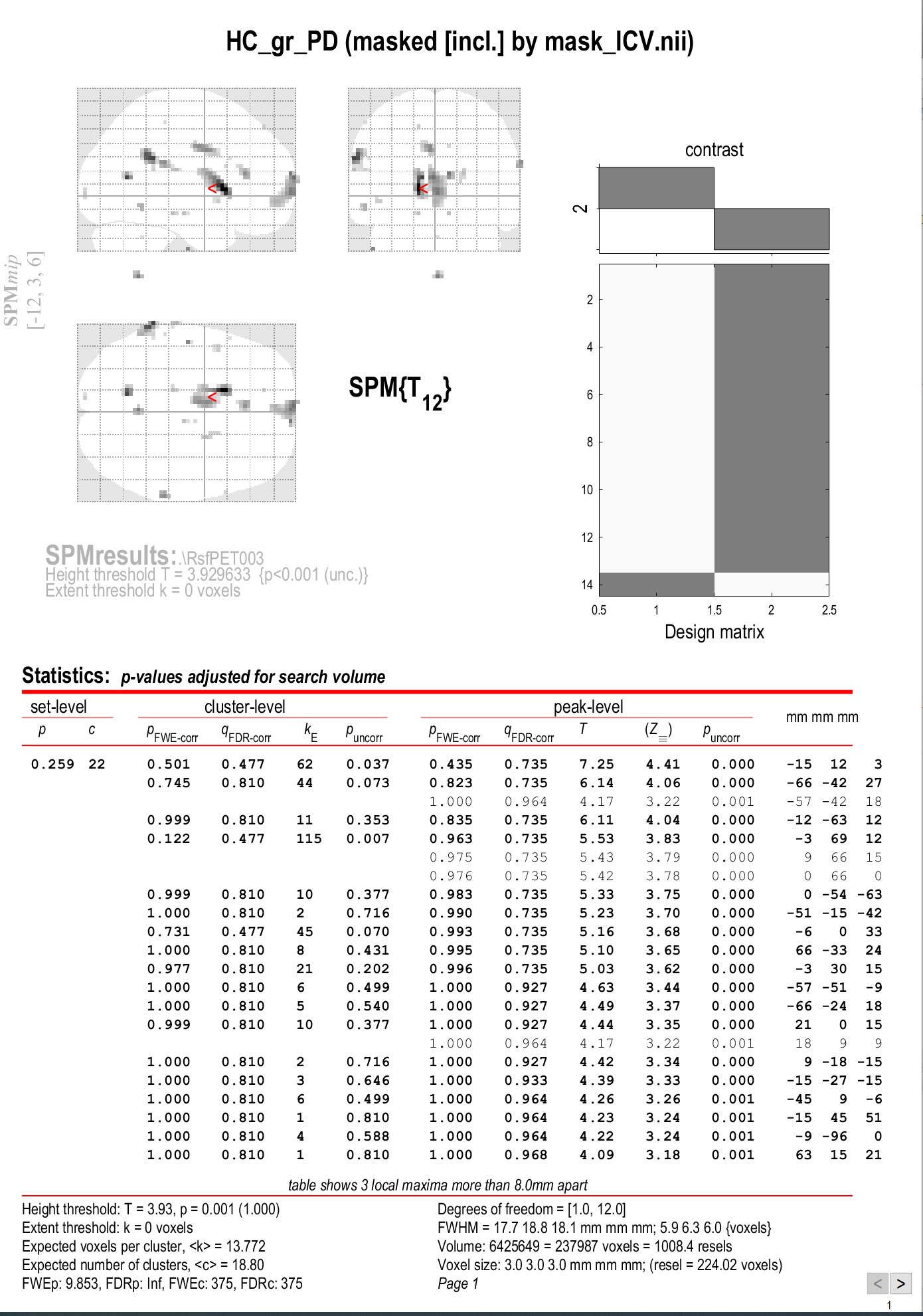


A


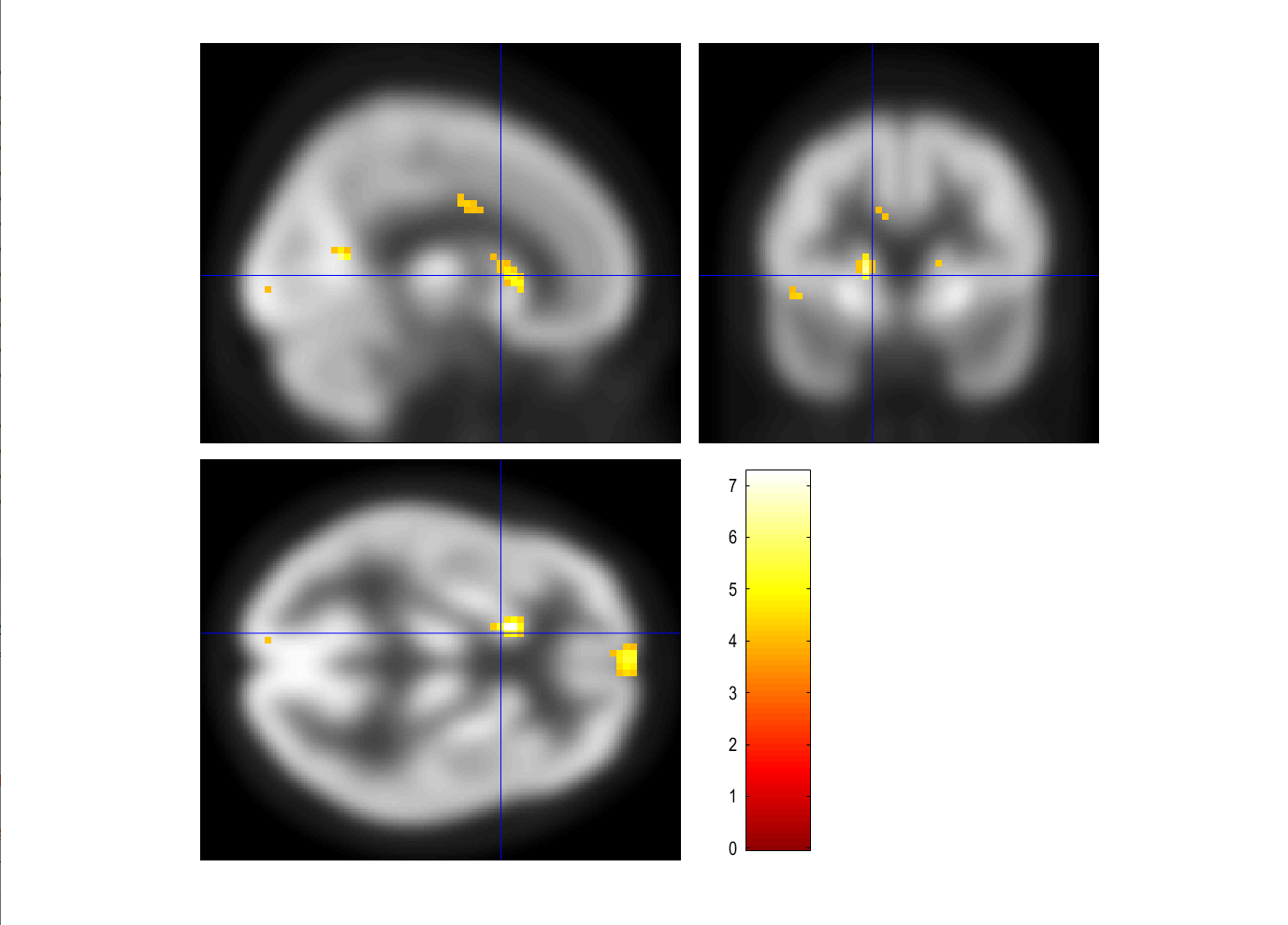


B

**Supplementary Fig. 9 Areas with relative hypometabolism in patient 3 in comparison to healthy controls** Single subject SPM analysis of mean 18F-FDG-PET scan (min 61-90) from subject one, which was voxel-wise compared to corresponding mean scans from the healthy control sample (T = 3.93, p < 0.001 uncorrected).

PD4 mean vs HC, PD>HC


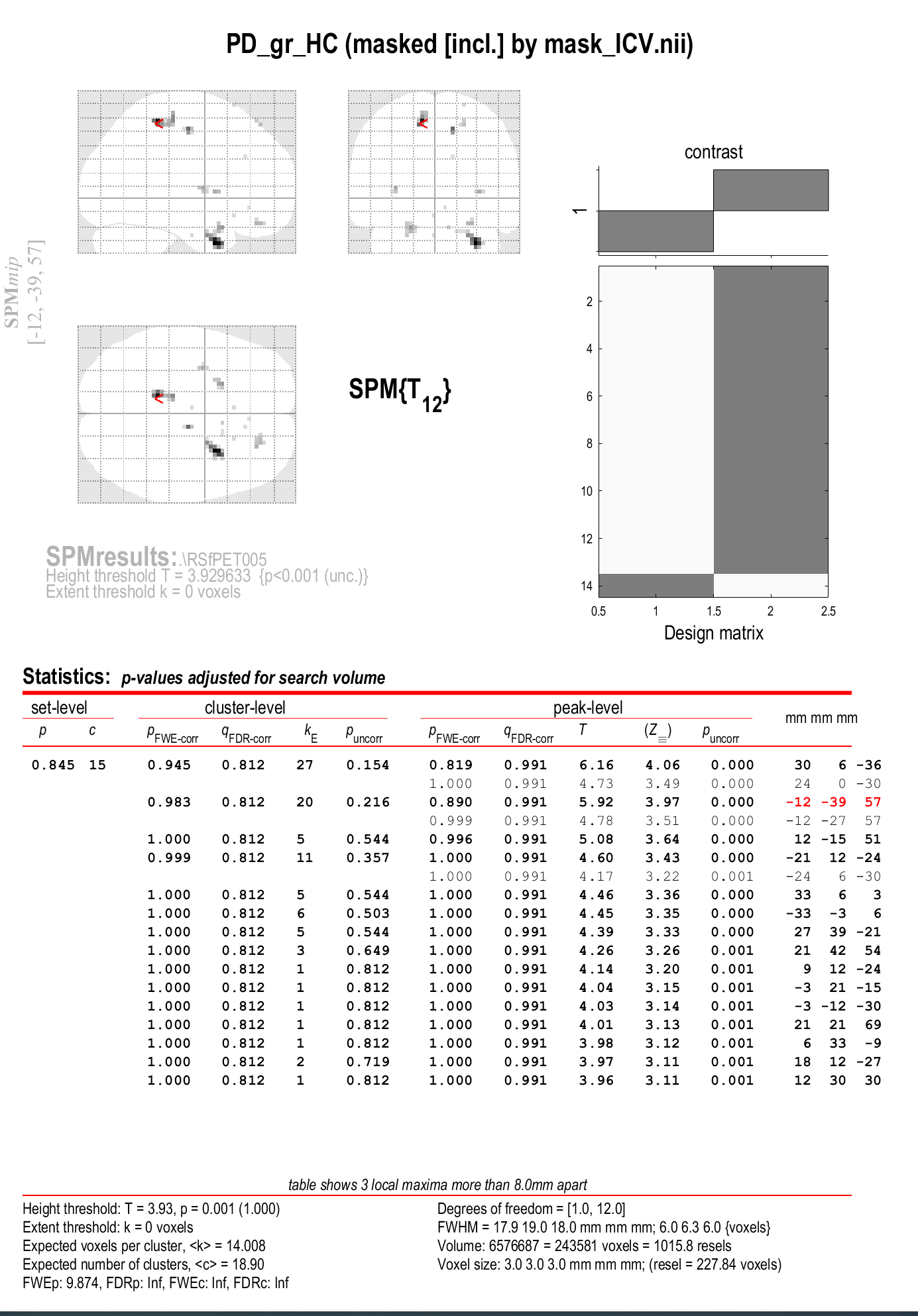


A


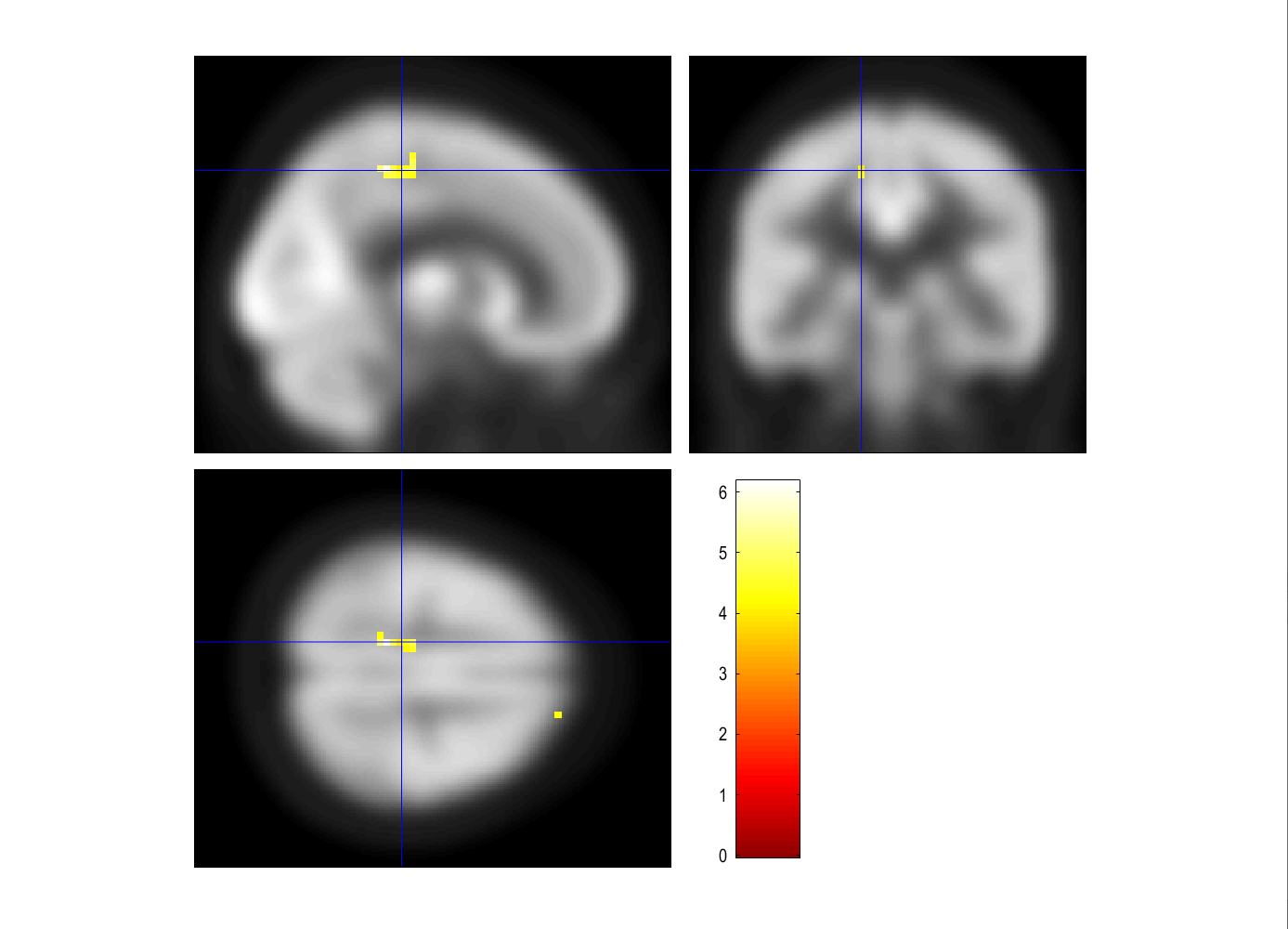


B

**Supplementary Fig. 10 Areas with relative hypermetabolism in patient 4 in comparison to healthy controls** Single subject SPM analysis of mean 18F-FDG-PET scan (min 61-90) from subject one, which was voxel-wise compared to corresponding mean scans from the healthy control sample (T = 3.93, p < 0.001 uncorrected).

PD4 mean vs HC, PD<HC


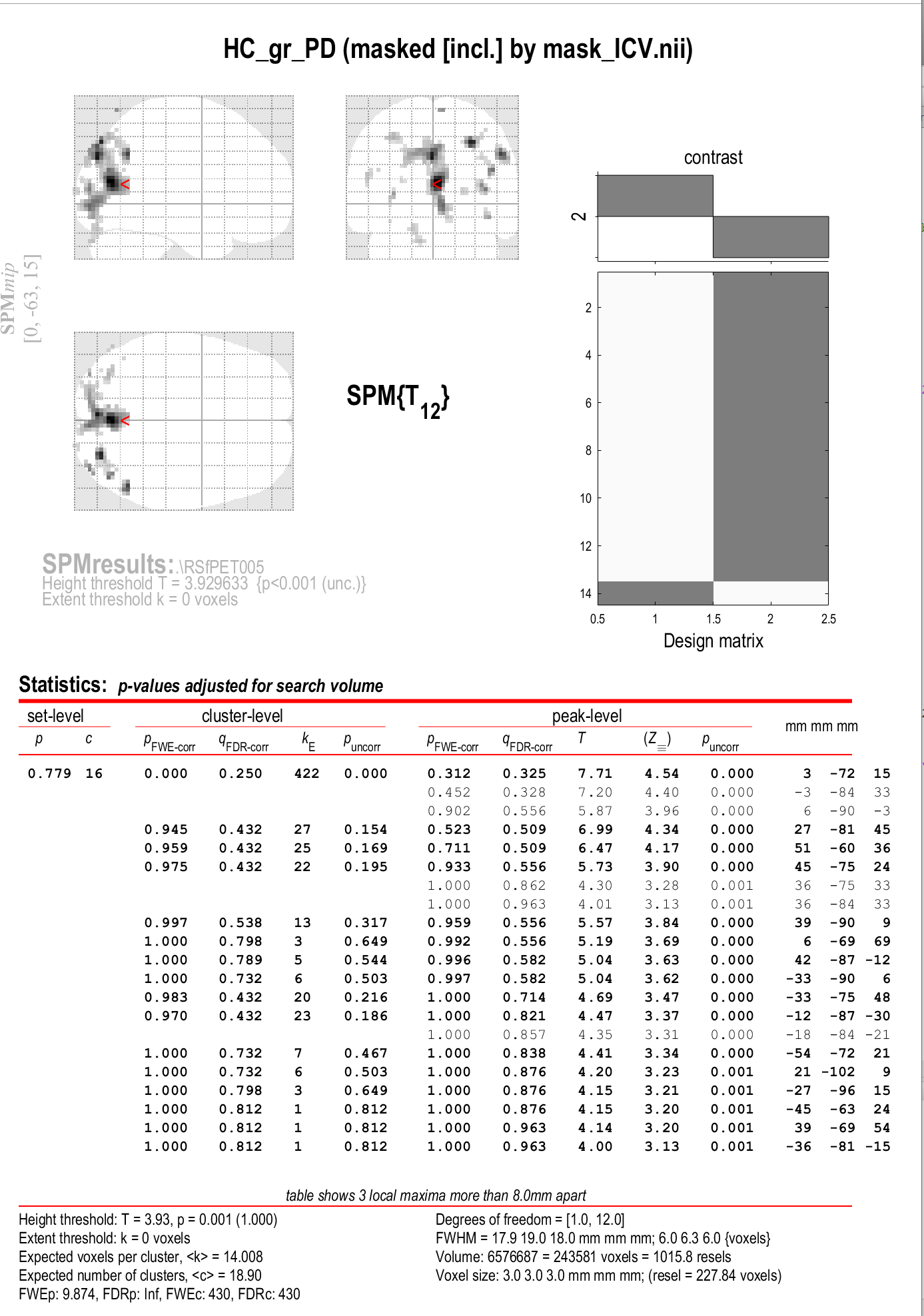


A


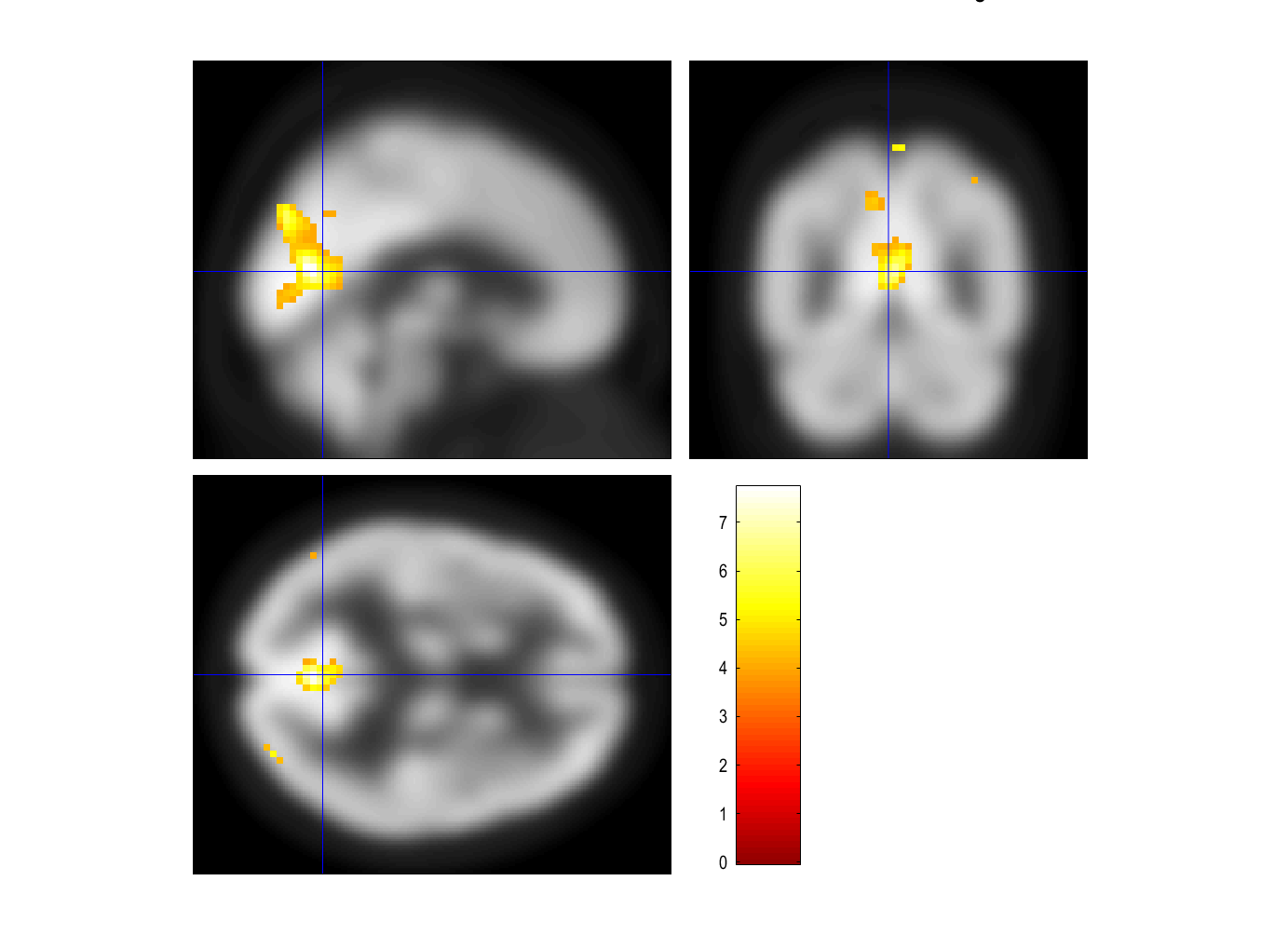


B

**Supplementary Fig. 11 Areas with relative hypometabolism in patient 4 in comparison to healthy controls** Single subject SPM analysis of mean 18F-FDG-PET scan (min 61-90) from subject one, which was voxel-wise compared to corresponding mean scans from the healthy control sample (T = 3.93, p < 0.001 uncorrected).

PD 6 mean vs HC, PD>HC


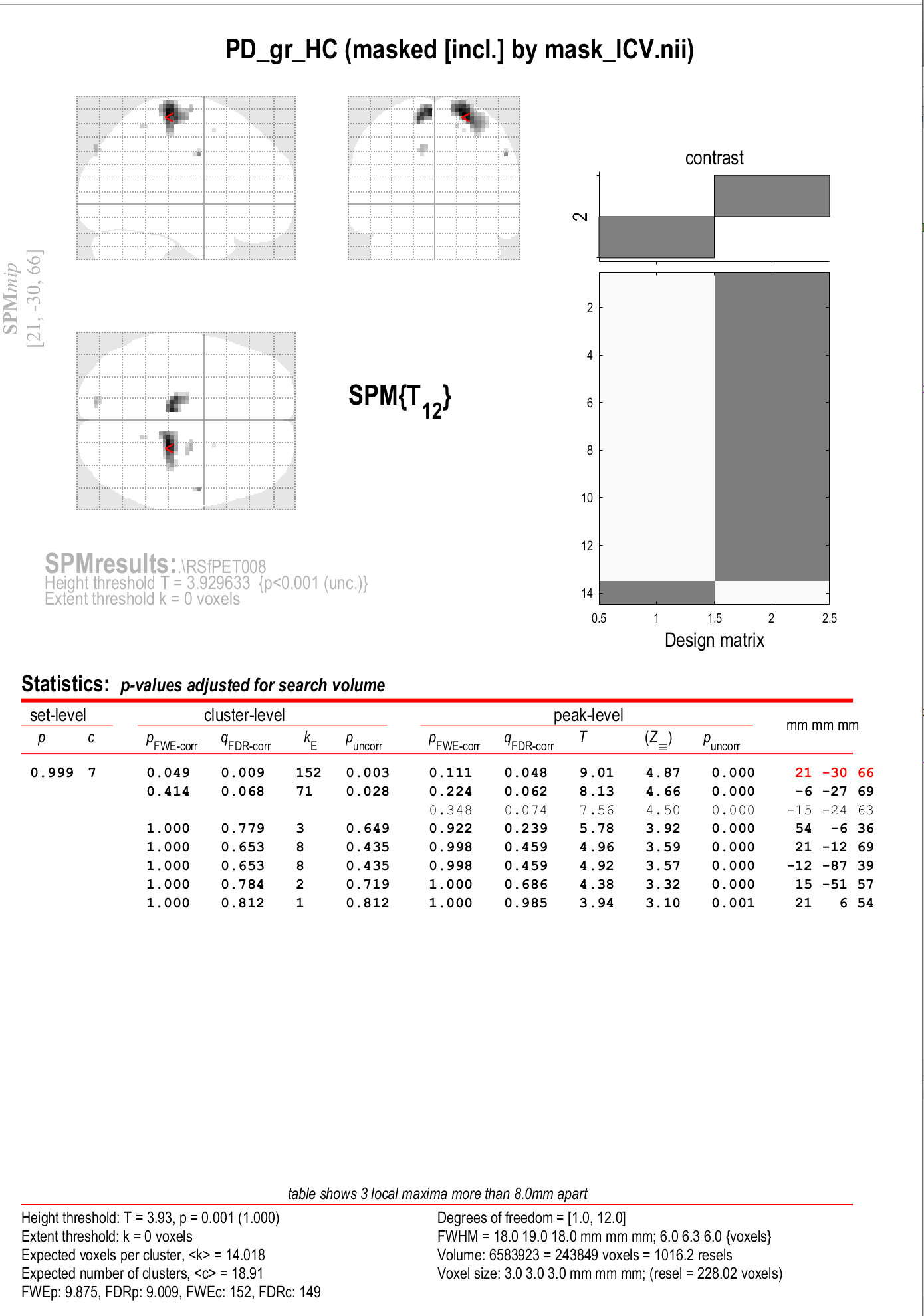


A


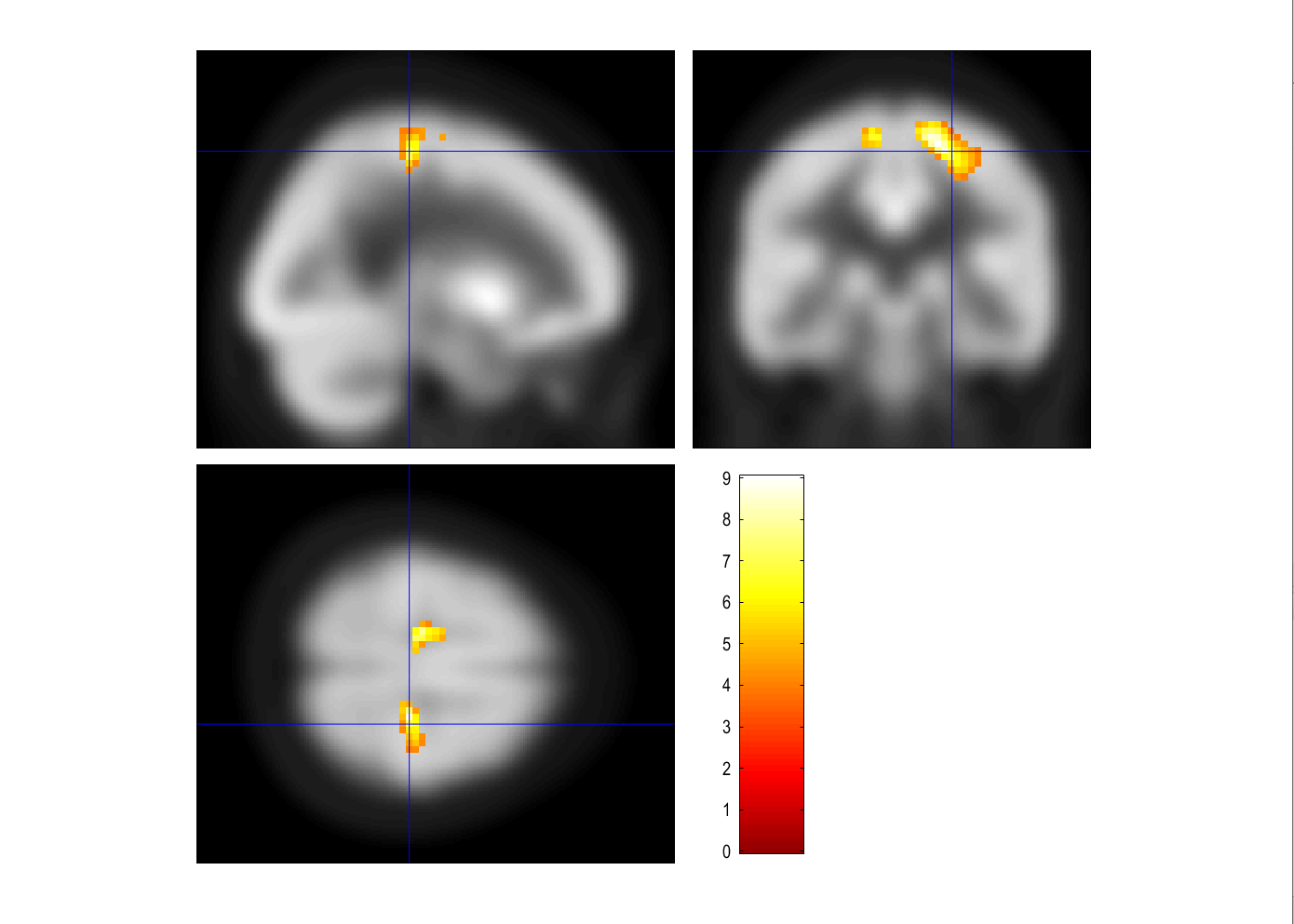


B

**Supplementary Fig. 12 Areas with relative hypermetabolism in patient 6 in comparison to healthy controls** Single subject SPM analysis of mean 18F-FDG-PET scan (min 61-90) from subject one, which was voxel-wise compared to corresponding mean scans from the healthy control sample (T = 3.93, p < 0.001 uncorrected).

PD7 mean vs HC, PD>HC


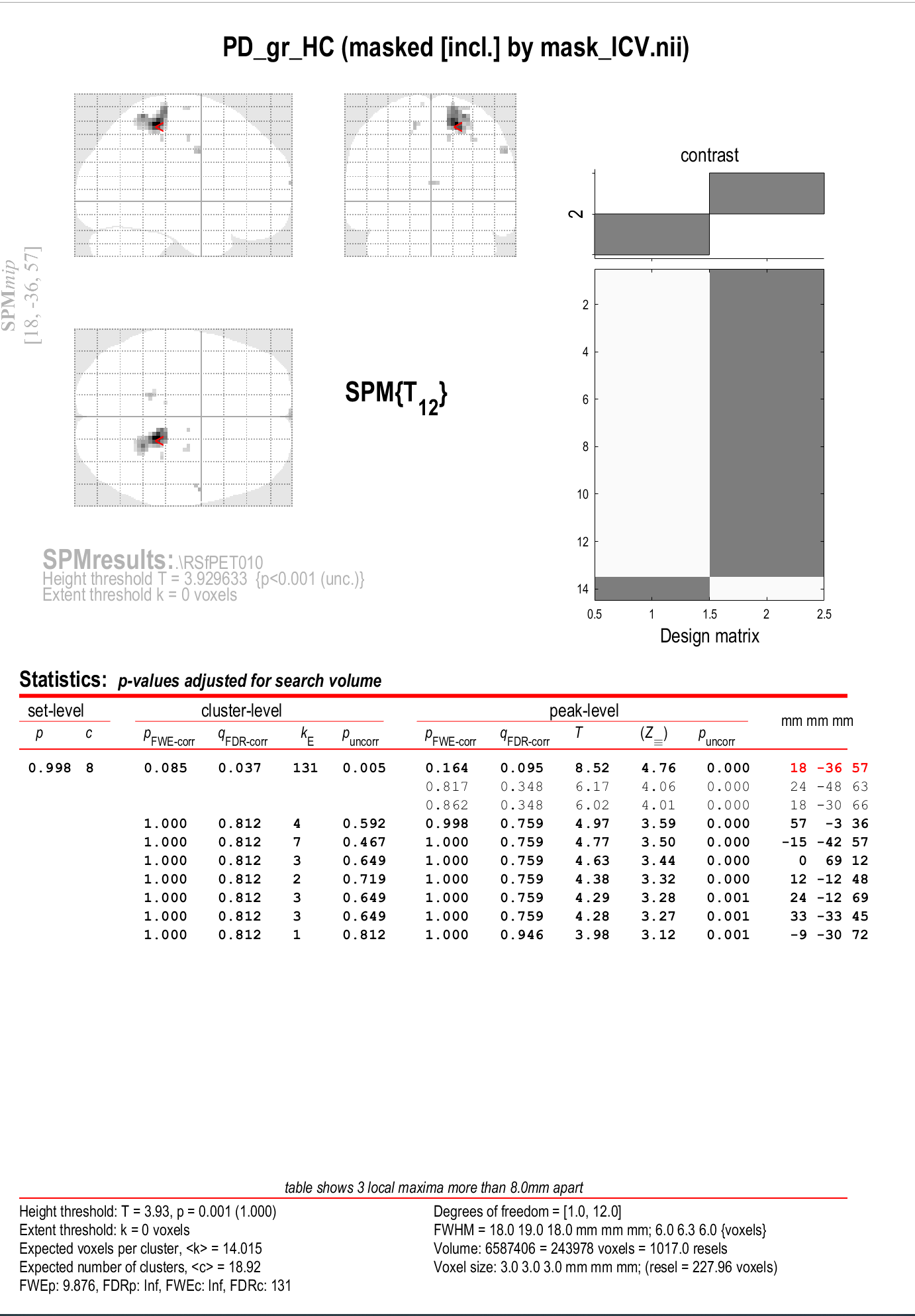


A

PD7 mean vs HC, PD>HC


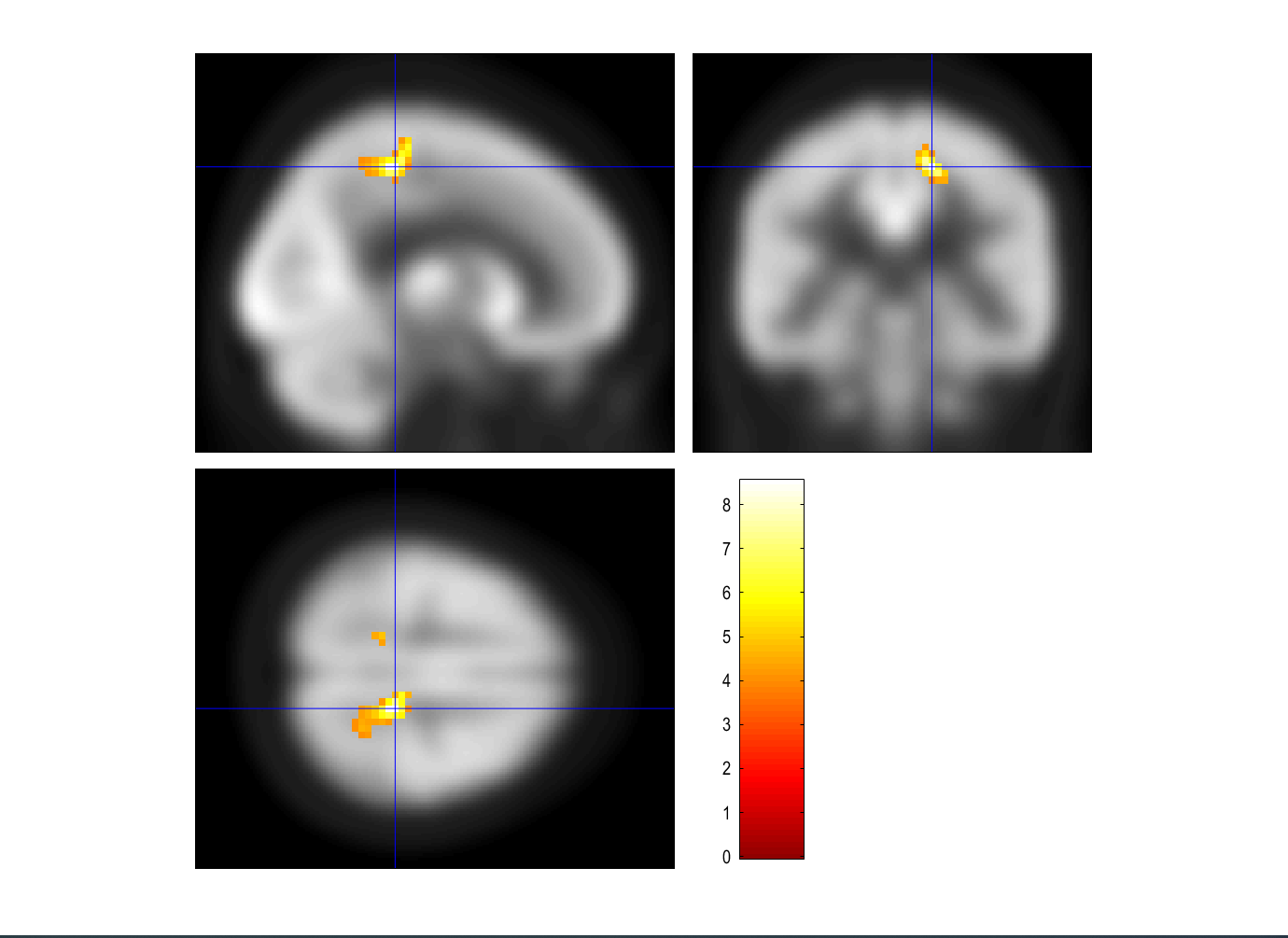


B

**Supplementary Fig. 13 Areas with relative hypermetabolism in patient 7 in comparison to healthy controls** Single subject SPM analysis of mean 18F-FDG-PET scan (min 61-90) from subject one, which was voxel-wise compared to corresponding mean scans from the healthy control sample (T = 3.93, p < 0.001 uncorrected).

PD8 mean vs HC, PD>HC


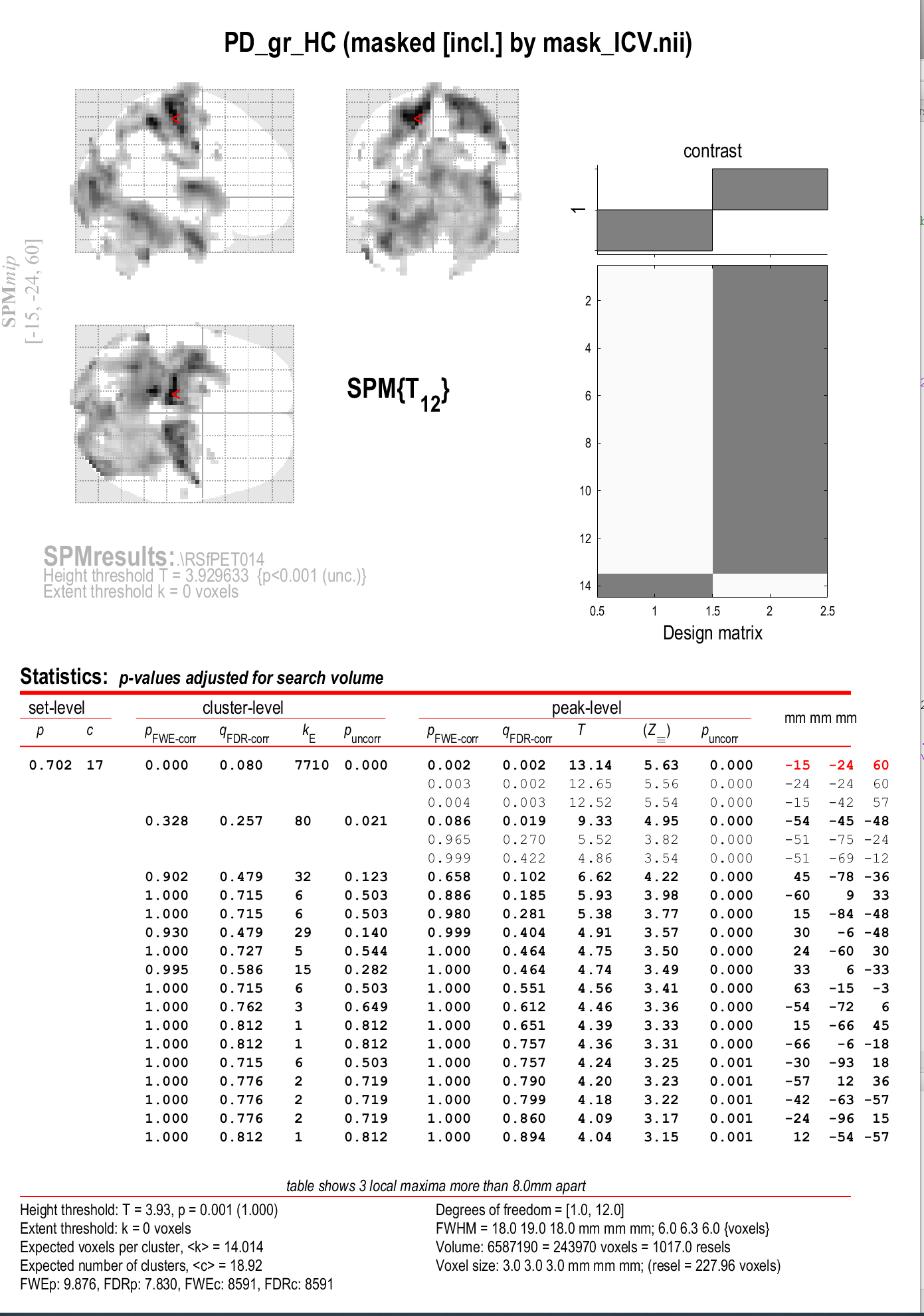


A


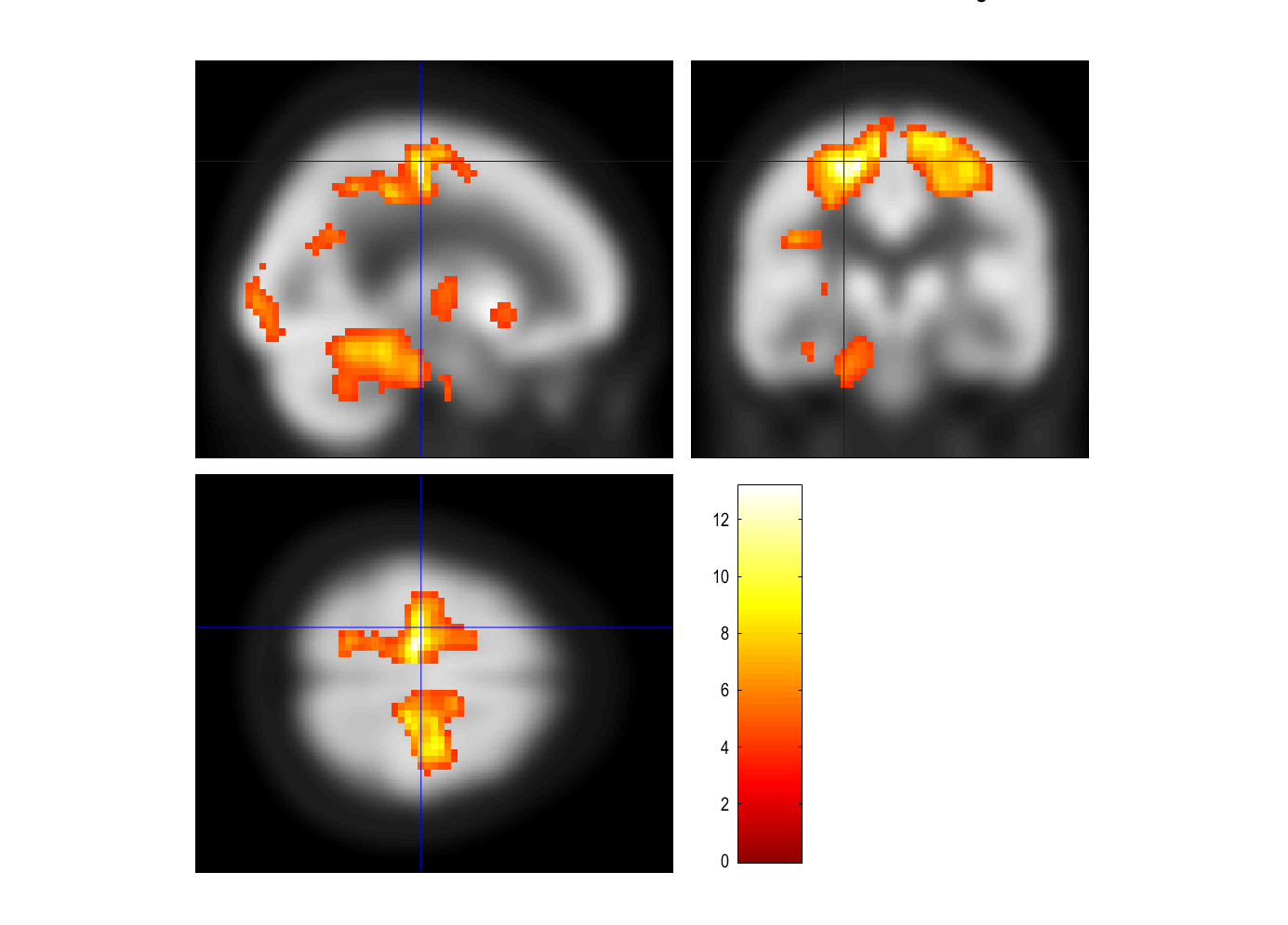


B

**Supplementary Fig. 14 Areas with relative hypermetabolism in patient 8 in comparison to healthy controls** Single subject SPM analysis of mean 18F-FDG-PET scan (min 61-90) from subject one, which was voxel-wise compared to corresponding mean scans from the healthy control sample (T = 3.93, p < 0.001 uncorrected).

PD8 mean vs HC, PD<HC


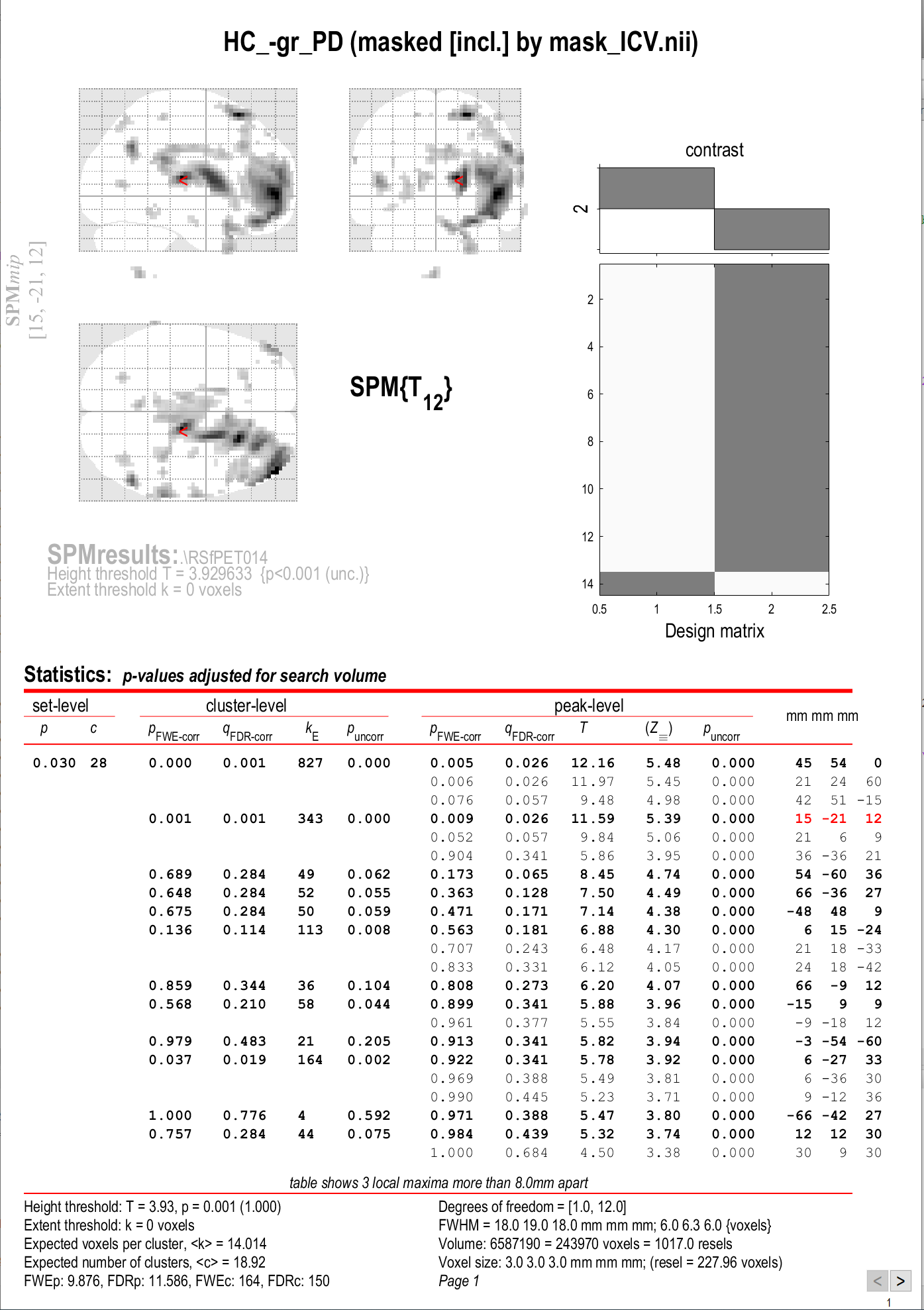


A


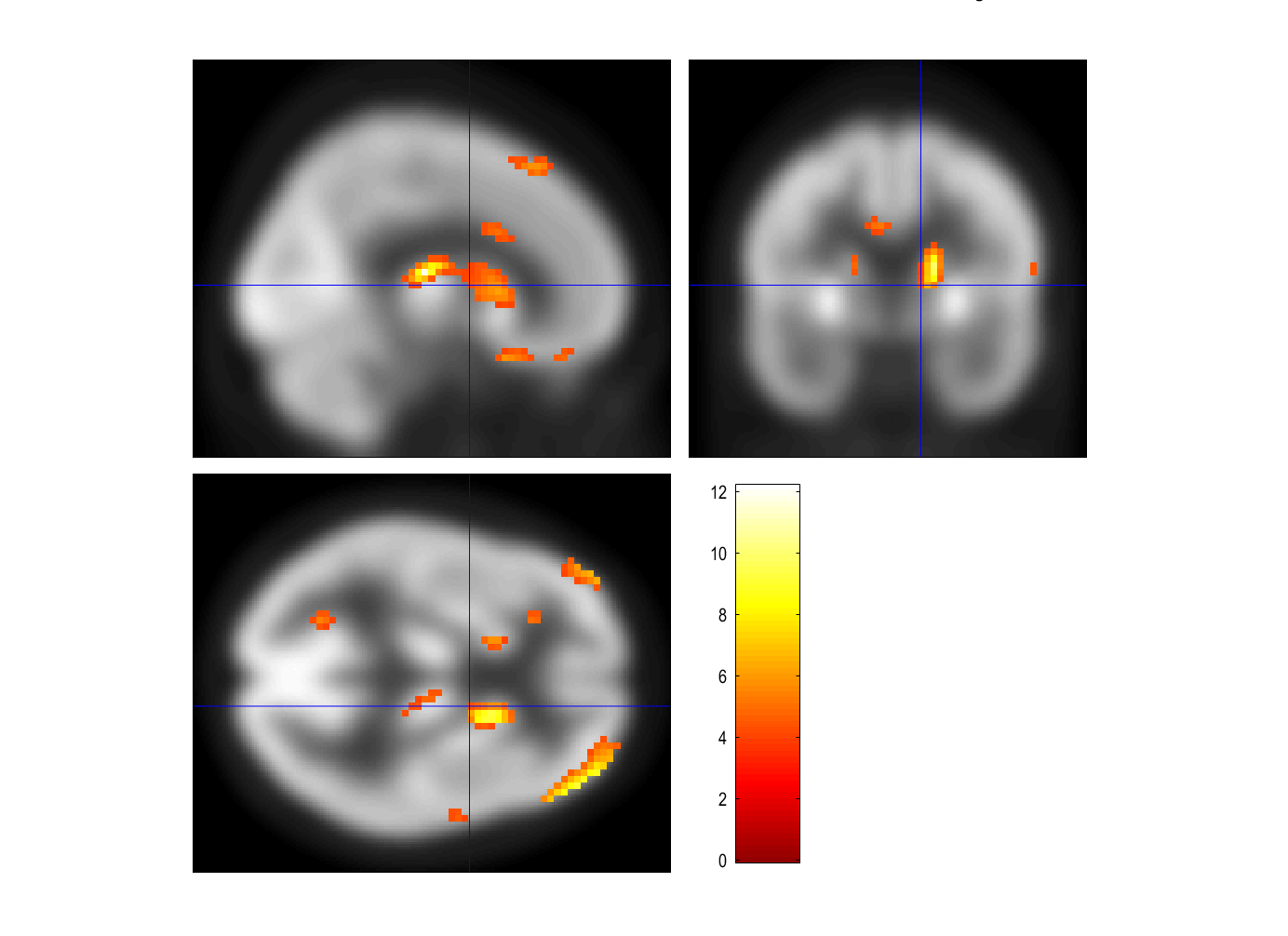


B

**Supplementary Fig. 15 Areas with relative hypometabolism in patient 8 in comparison to healthy controls** Single subject SPM analysis of mean 18F-FDG-PET scan (min 61-90) from subject one, which was voxel-wise compared to corresponding mean scans from the healthy control sample (T = 3.93, p < 0.001 uncorrected).

PD 9 mean vs HC, PD>HC


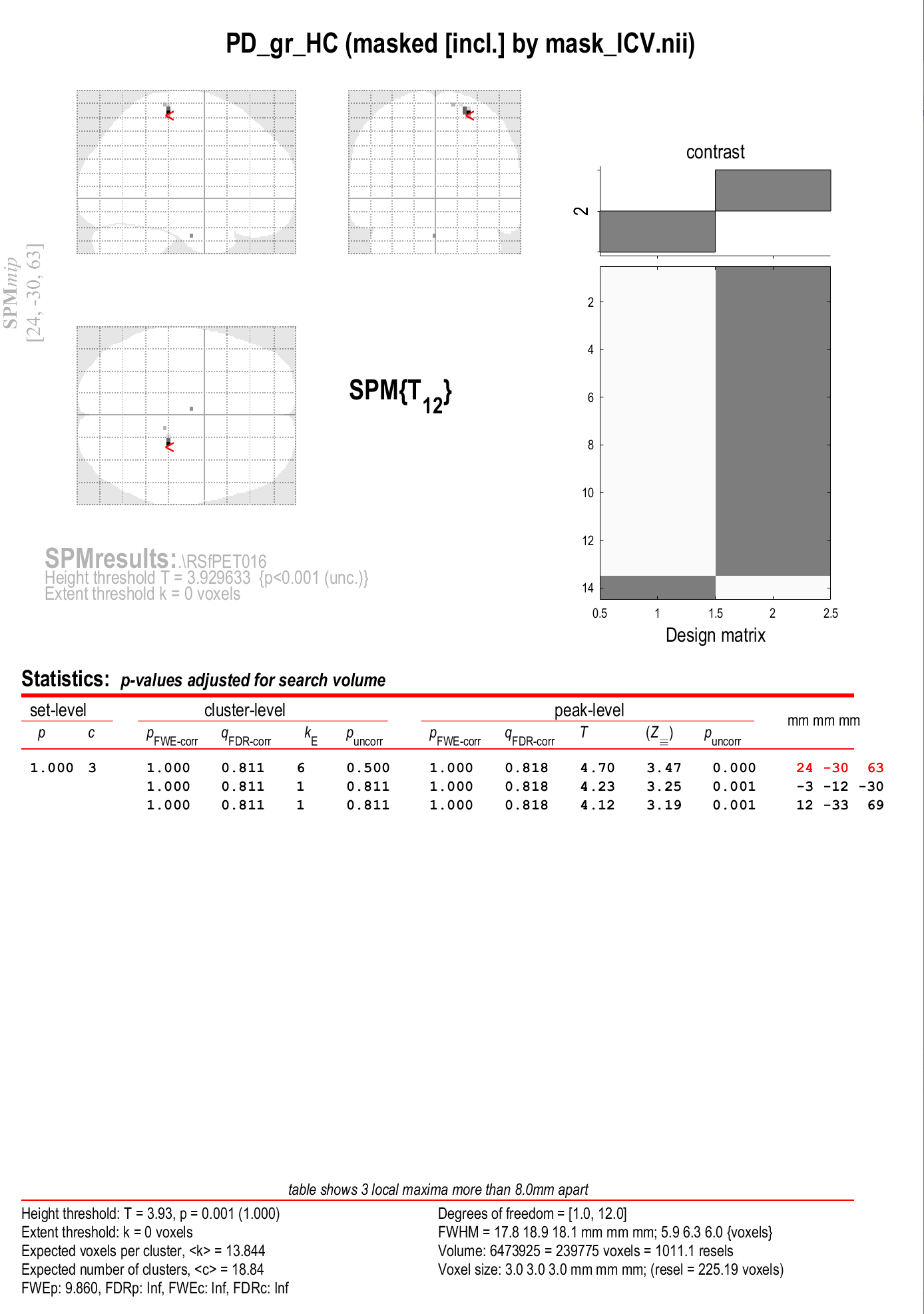


A


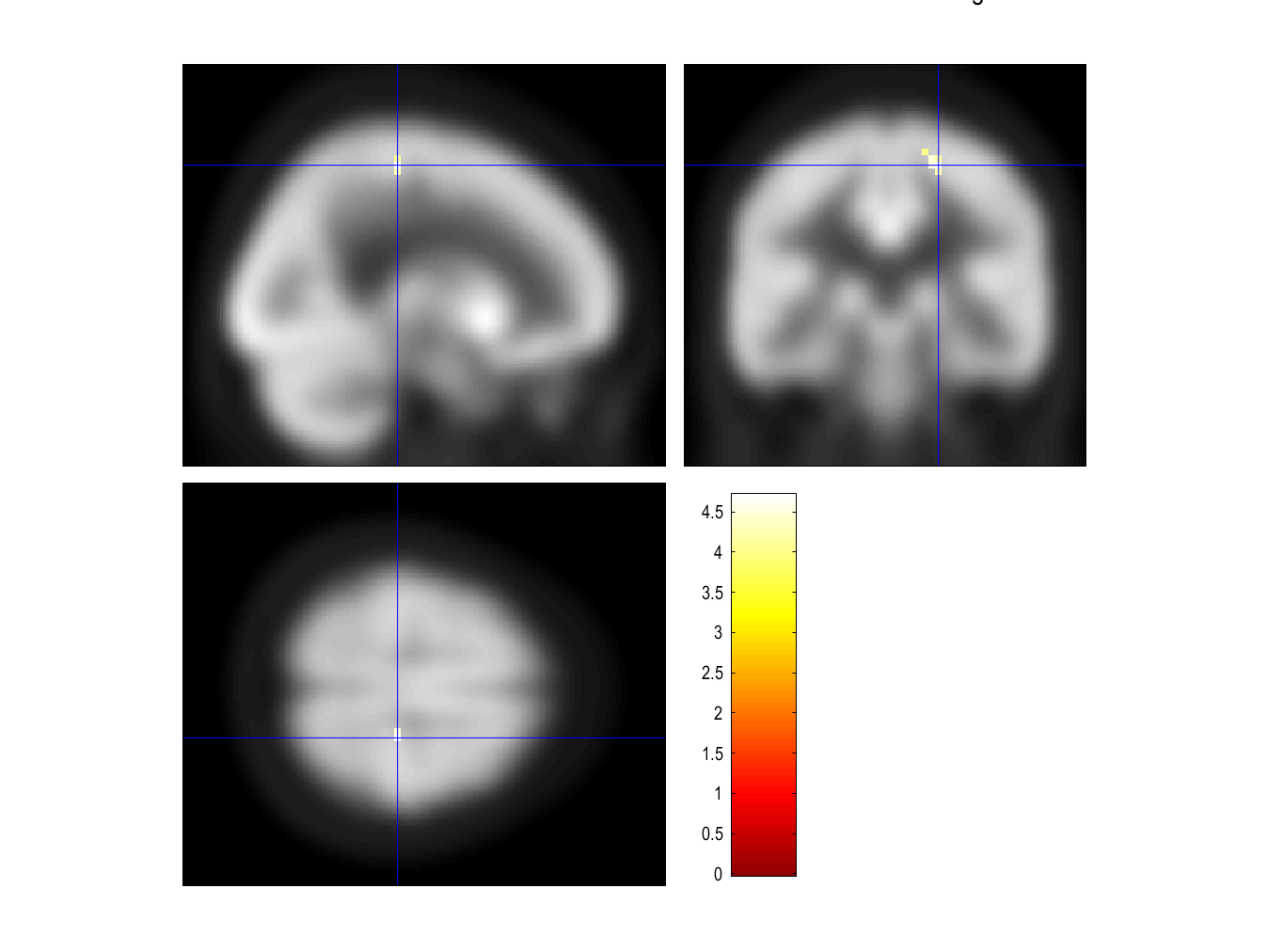


B

**Supplementary Fig. 16 Areas with relative hypermetabolism in patient 9 in comparison to healthy controls** Single subject SPM analysis of mean 18F-FDG-PET scan (min 61-90) from subject one, which was voxel-wise compared to corresponding mean scans from the healthy control sample (T = 3.93, p < 0.001 uncorrected).

PD9 mean vs HC, PD<HC


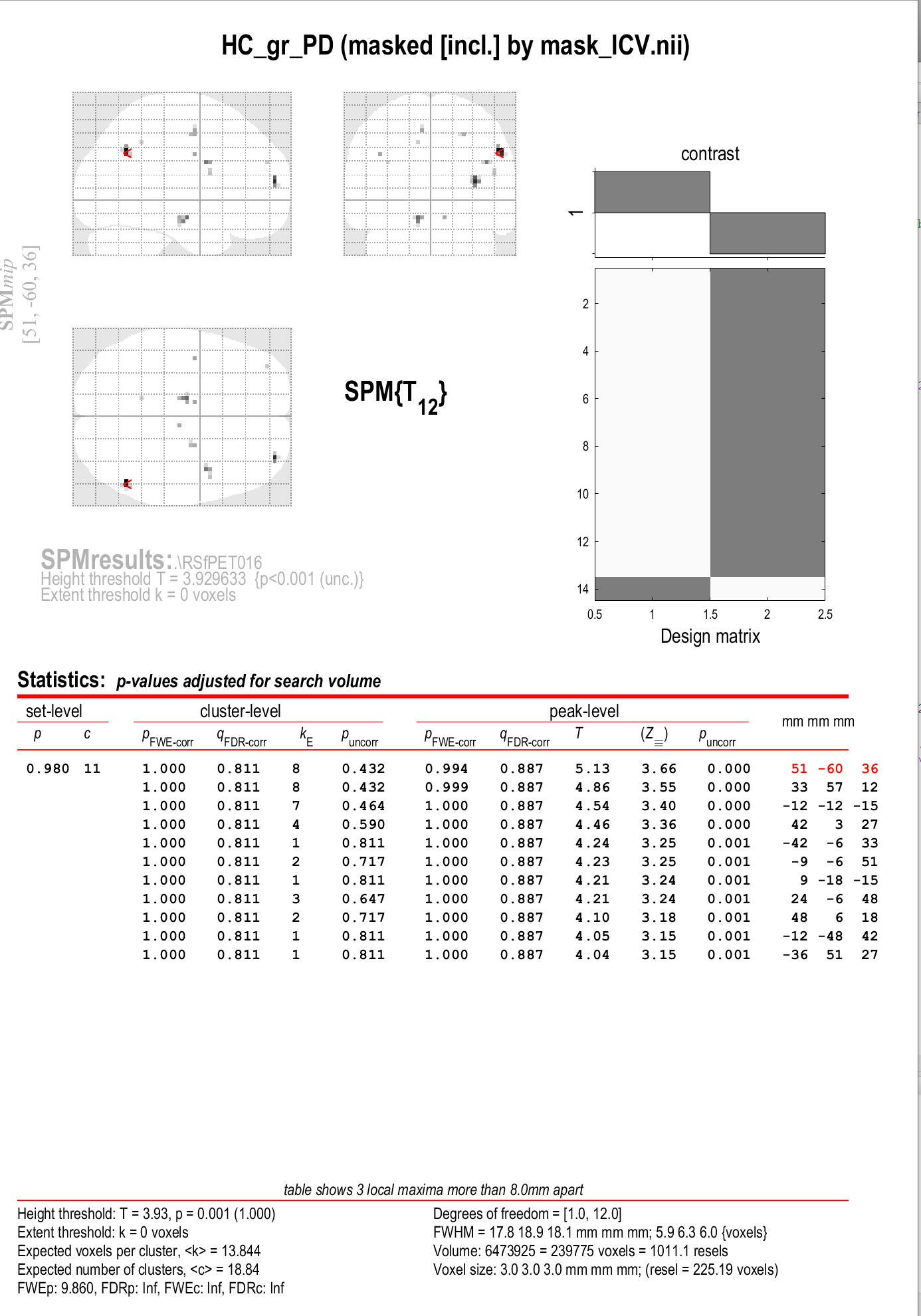


**Supplementary Fig. 17 Areas with relative hypometabolism in patient 9 in comparison to healthy controls** Single subject SPM analysis of mean 18F-FDG-PET scan (min 61-90) from subject one, which was voxel-wise compared to corresponding mean scans from the healthy control sample (T = 3.93, p < 0.001 uncorrected).

PD10 mean vs HC, PD>HC


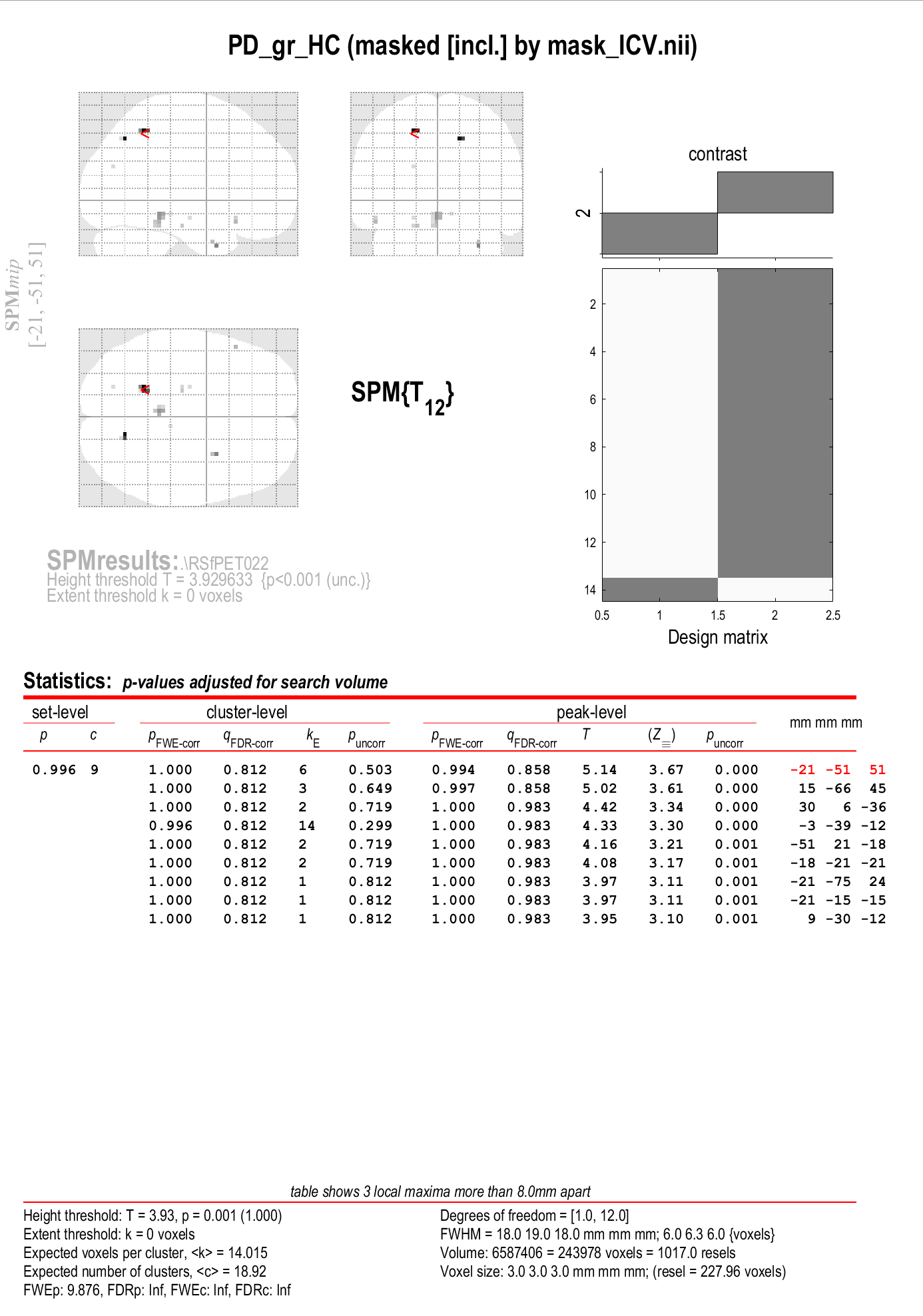


A


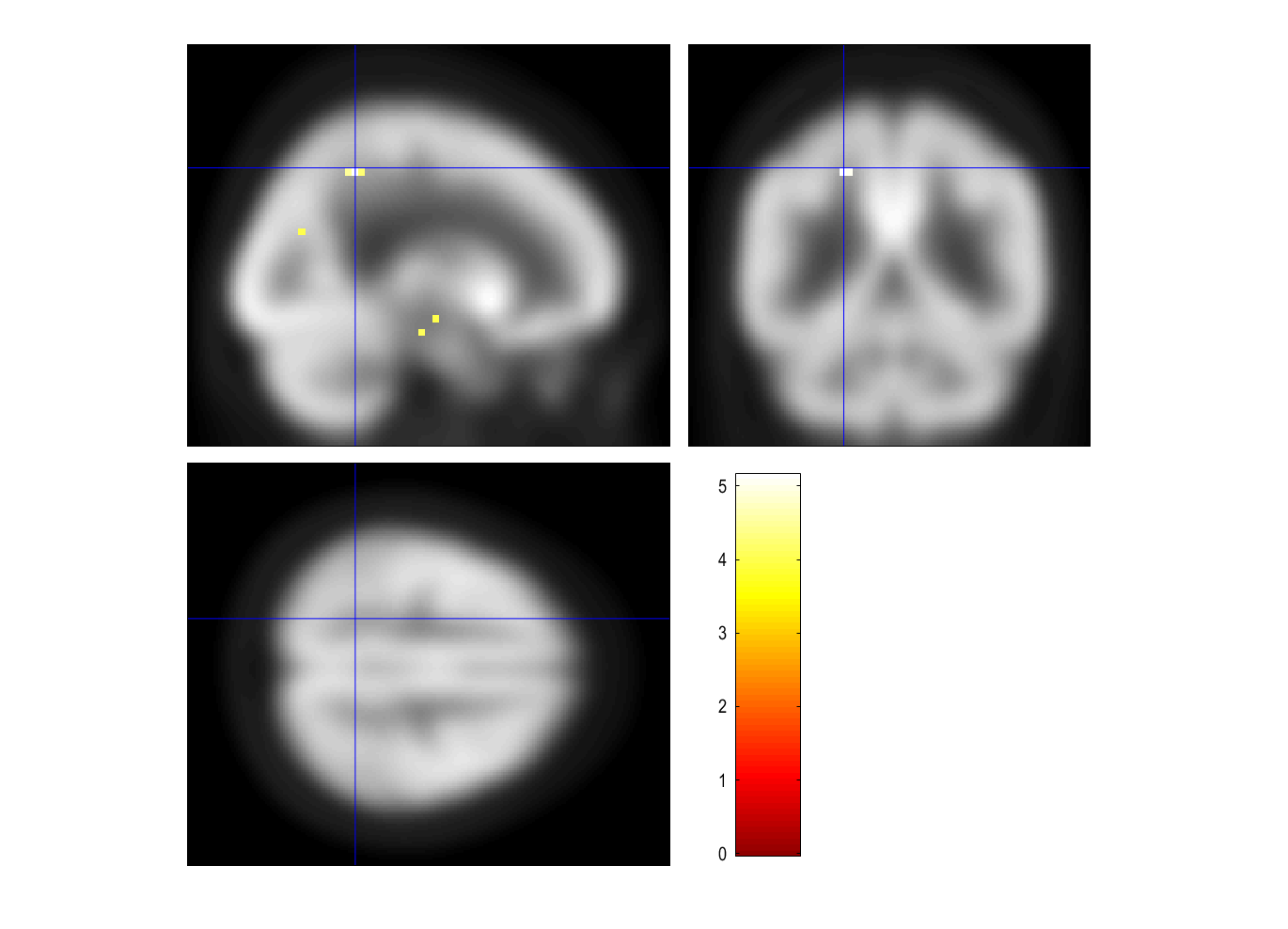


B

**Supplementary Fig. 18 Areas with relative hypermetabolism in patient 10 in comparison to healthy controls** Single subject SPM analysis of mean 18F-FDG-PET scan (min 61-90) from subject one, which was voxel-wise compared to corresponding mean scans from the healthy control sample (T = 3.93, p < 0.001 uncorrected).

PD11 mean vs HC, PD>HC


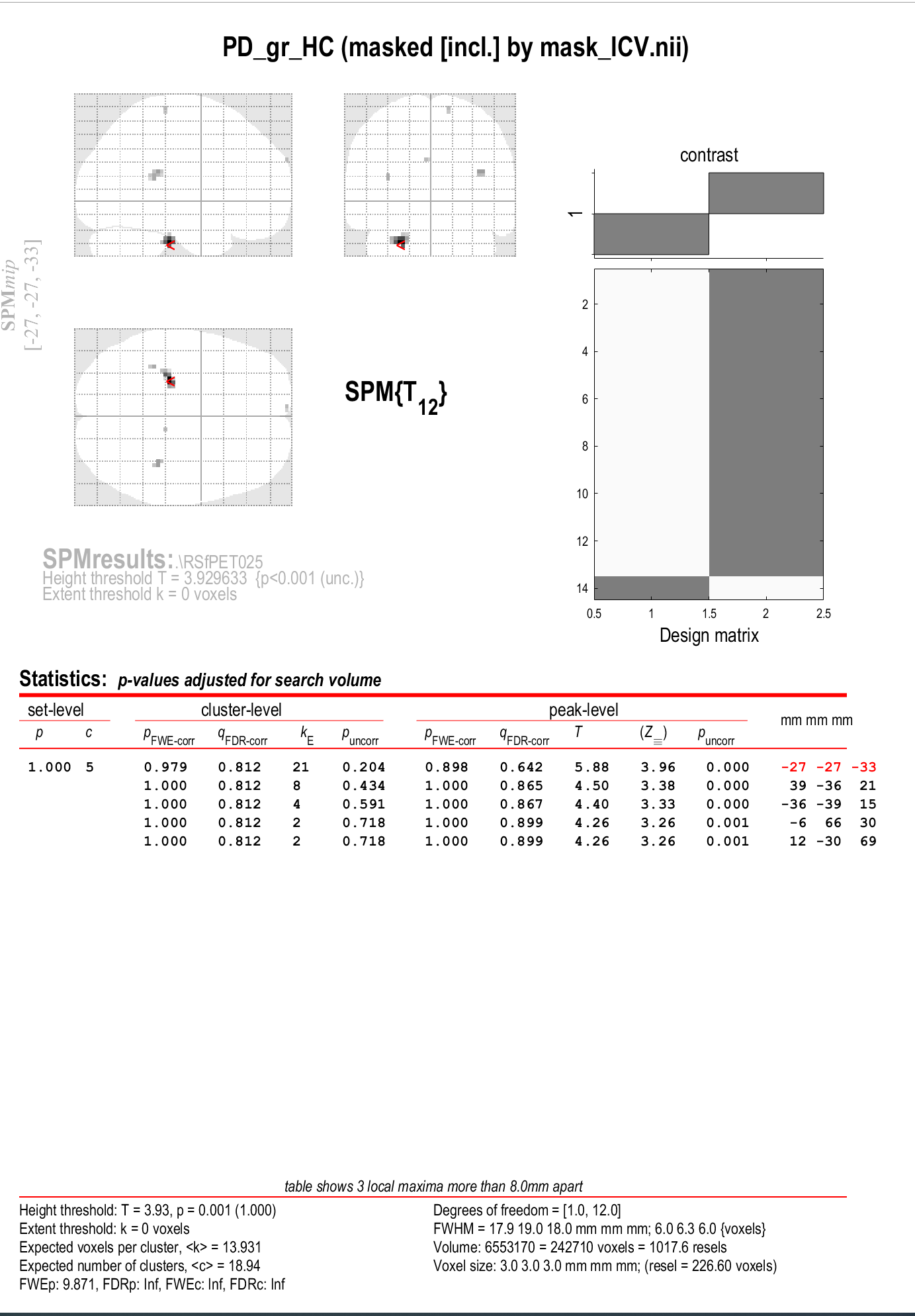


A


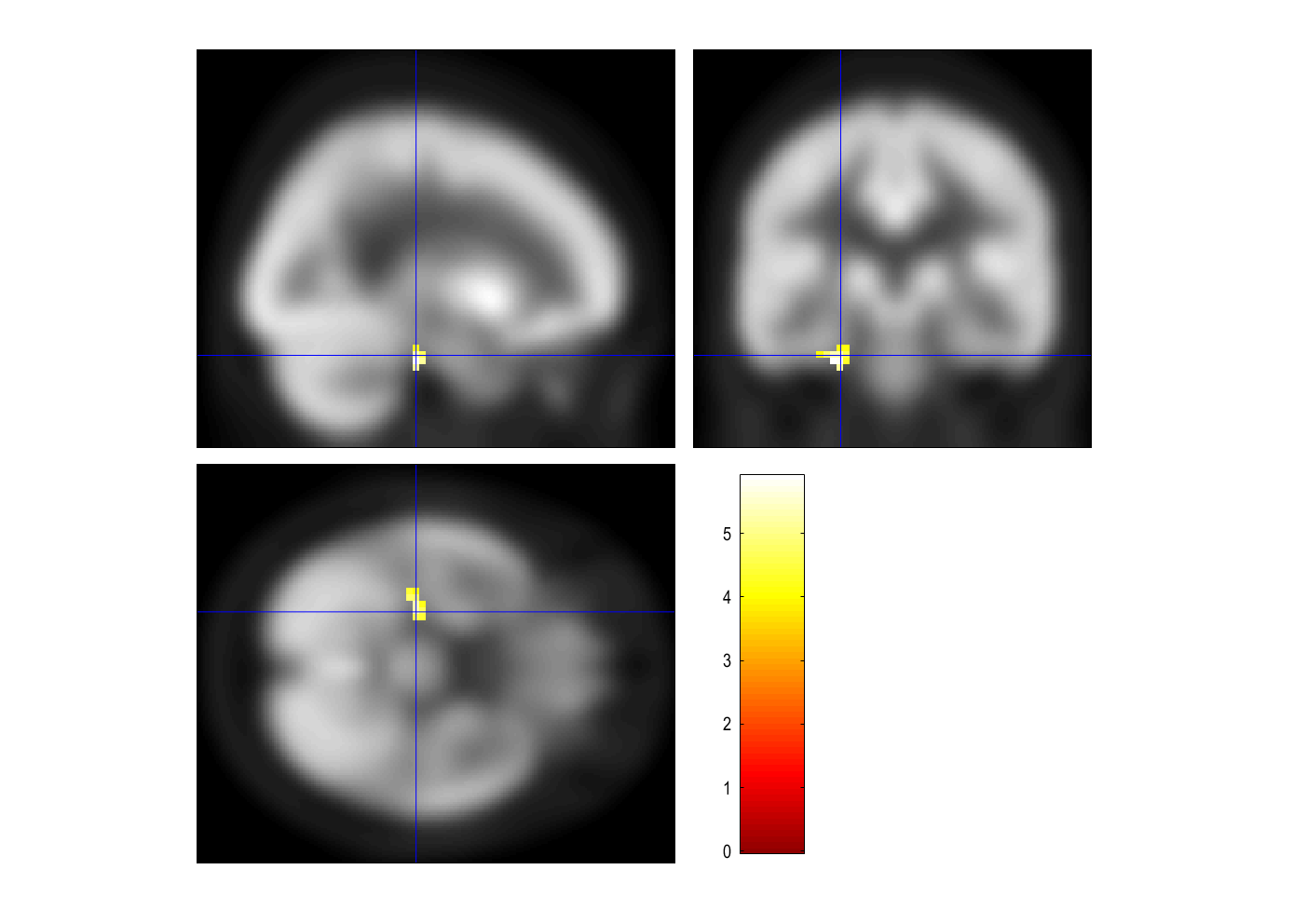


B

**Supplementary Fig. 19 Areas with relative hypermetabolism in patient 11 in comparison to healthy controls** Single subject SPM analysis of mean 18F-FDG-PET scan (min 61-90) from subject one, which was voxel-wise compared to corresponding mean scans from the healthy control sample (T = 3.93, p < 0.001 uncorrected).

PD11 mean vs HC, PD<HC


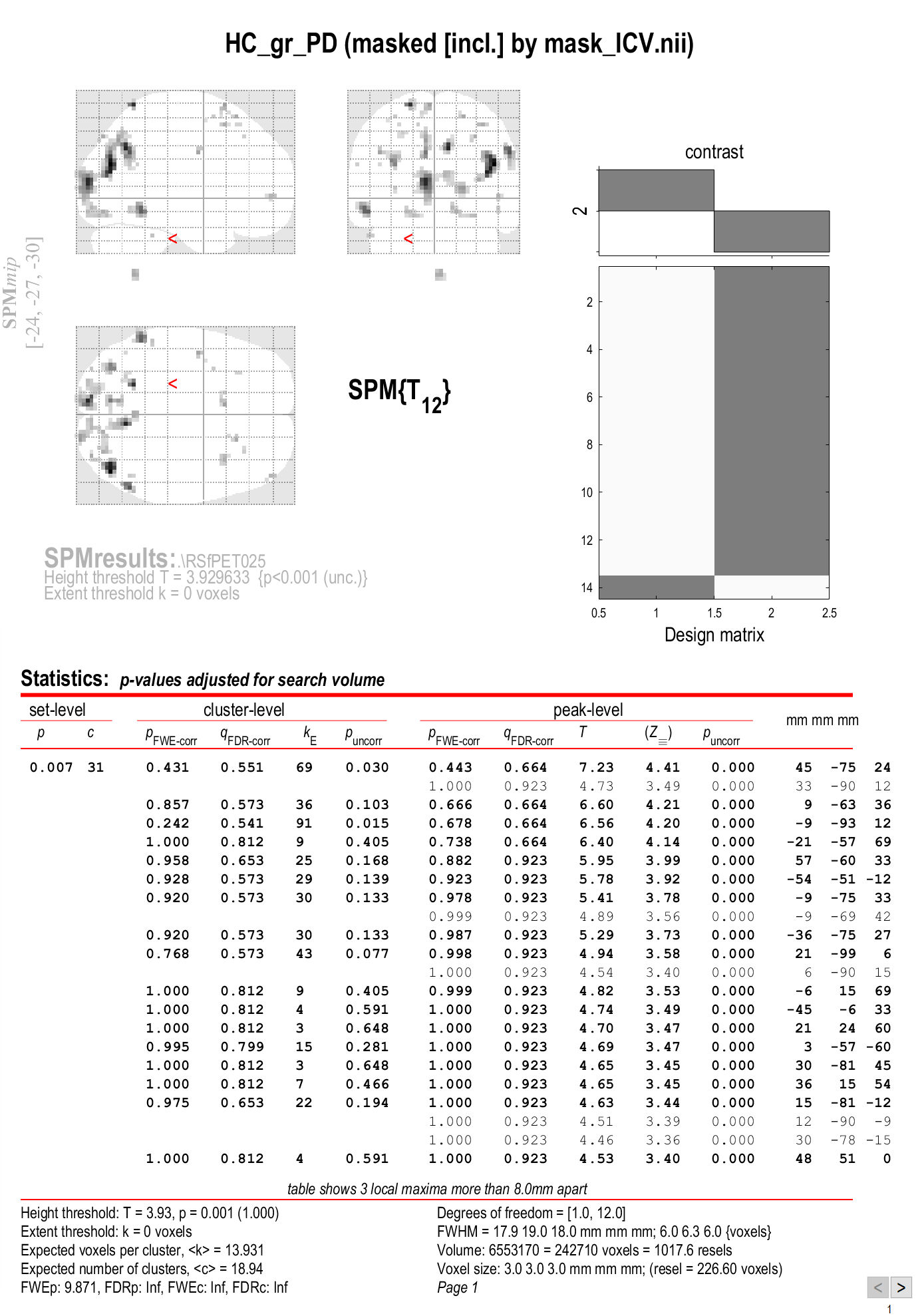


A


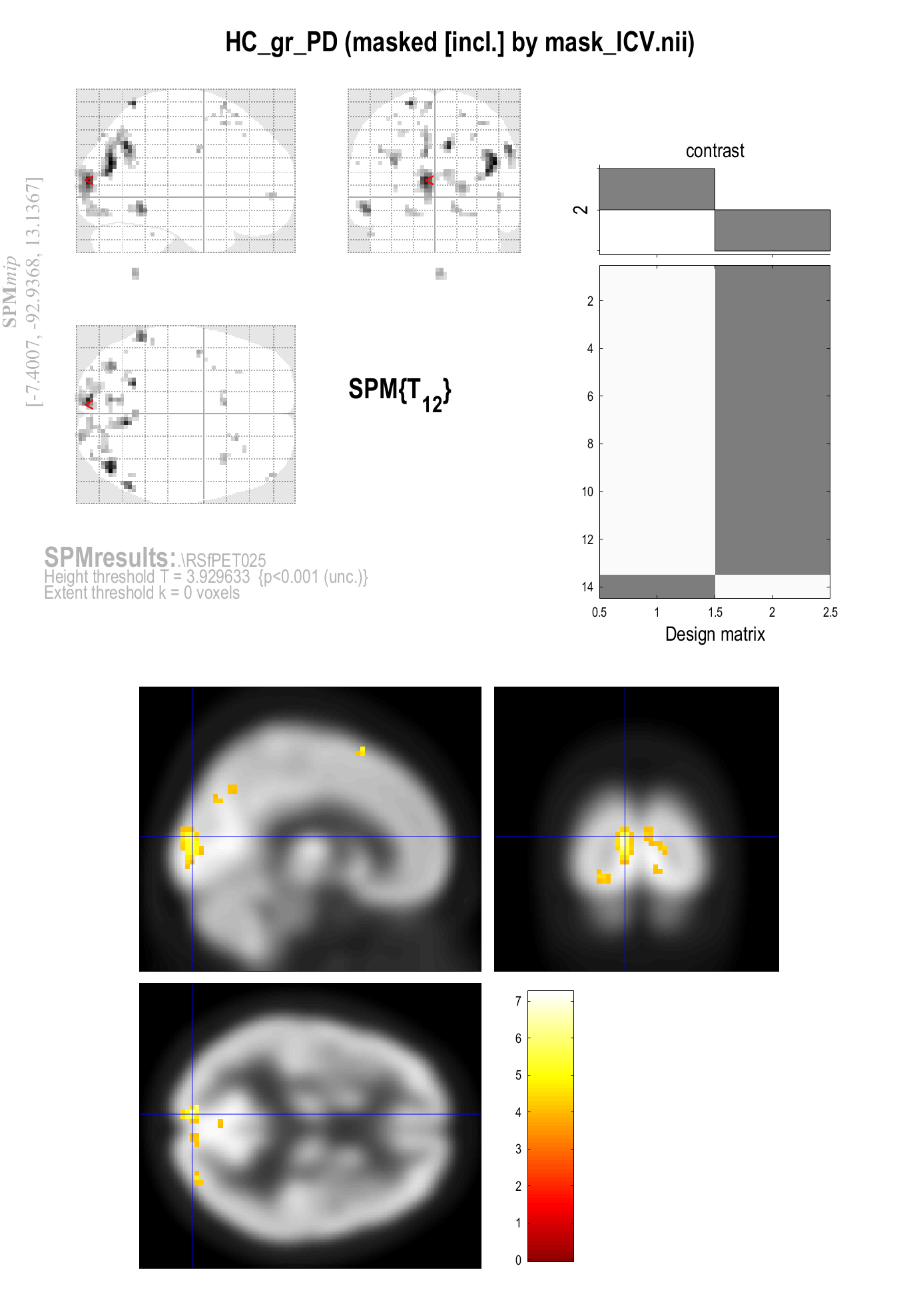


B

**Supplementary Fig. 20 Areas with relative hypometabolism in patient 11 in comparison to healthy controls** Single subject SPM analysis of mean 18F-FDG-PET scan (min 61-90) from subject one, which was voxel-wise compared to corresponding mean scans from the healthy control sample (T = 3.93, p < 0.001 uncorrected).

PD 12 mean vs HC, PD>HC


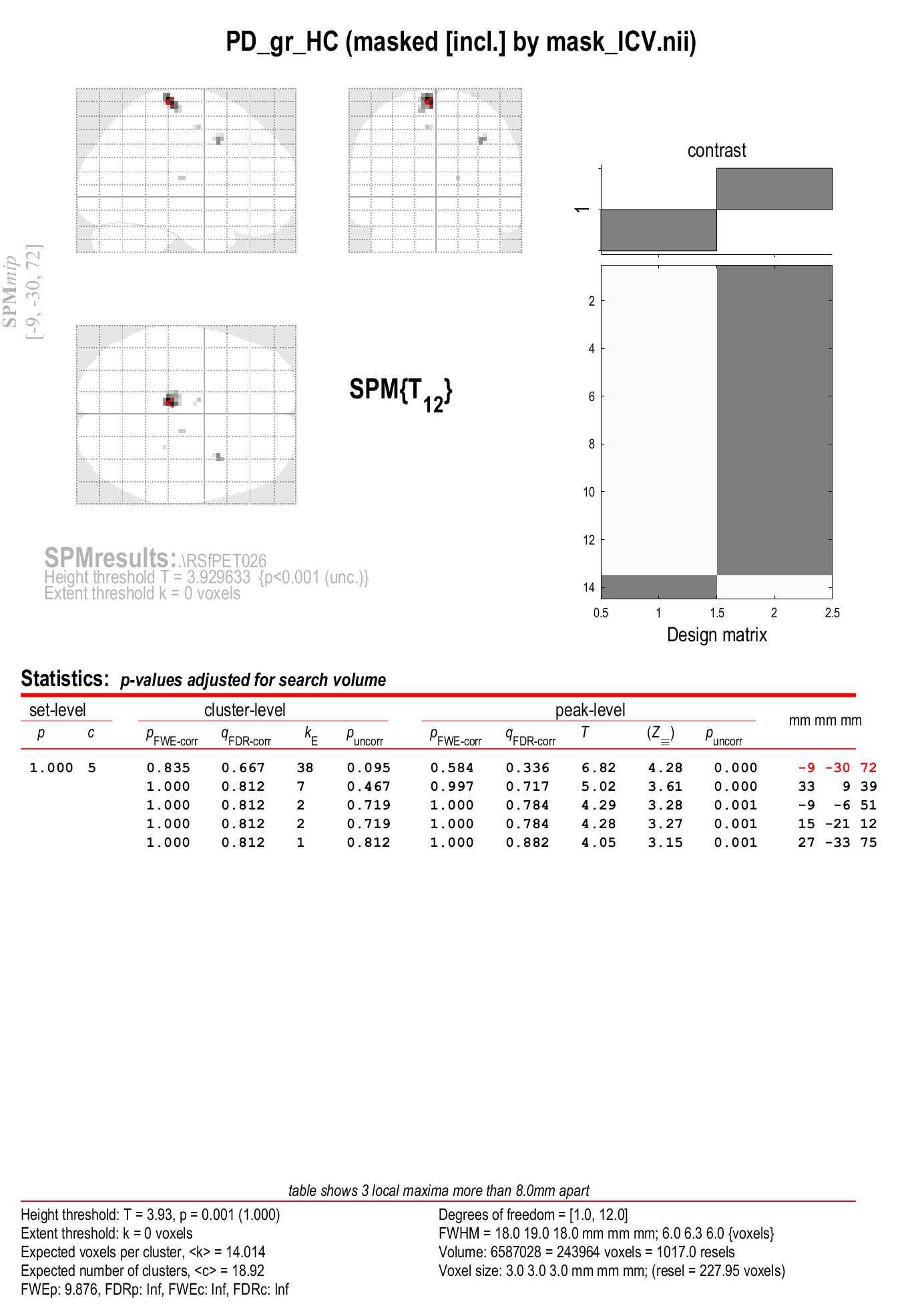


A


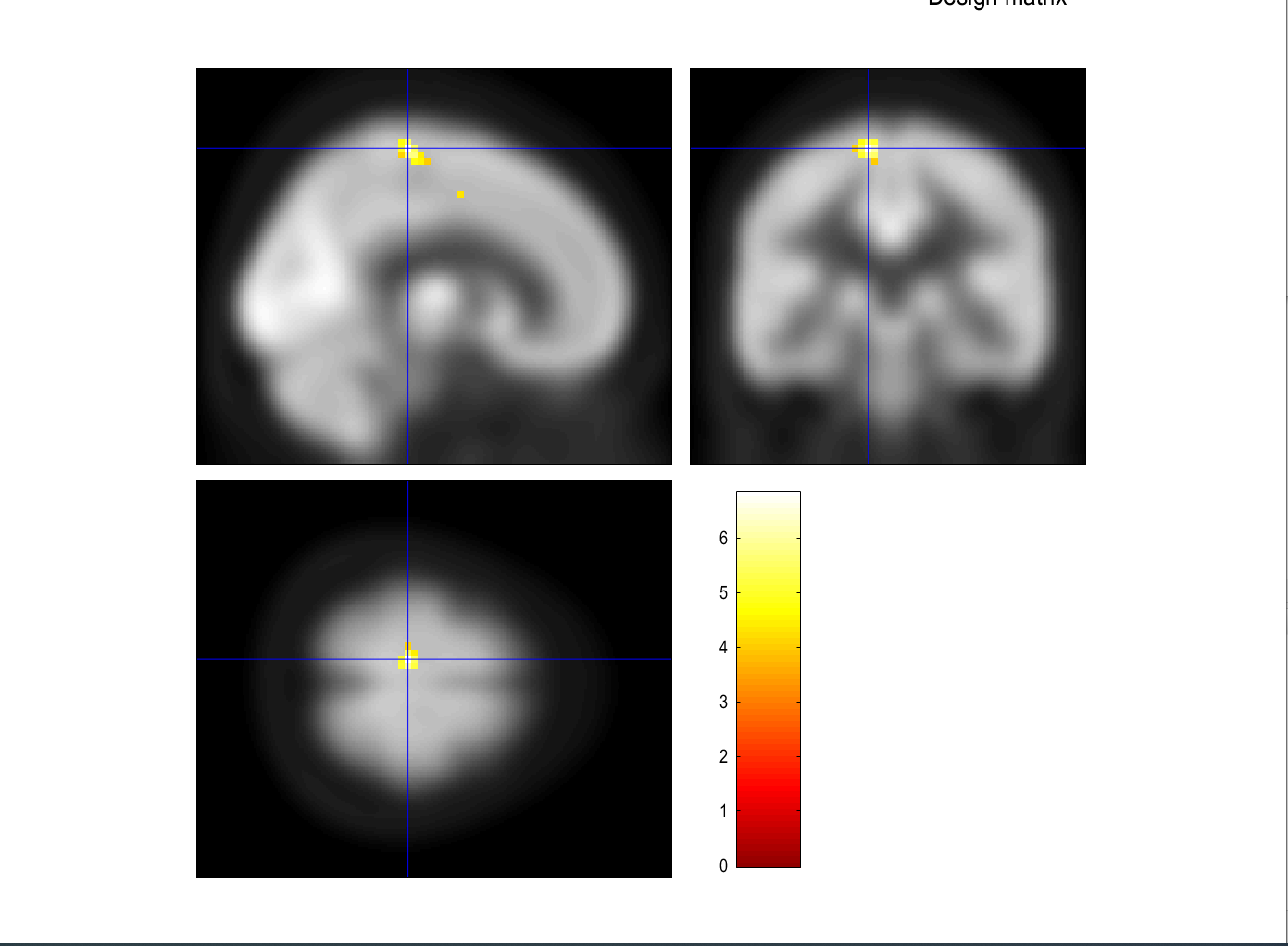


B

**Supplementary Fig. 21 Areas with relative hypermetabolism in patient 12 in comparison to healthy controls** Single subject SPM analysis of mean 18F-FDG-PET scan (min 61-90) from subject one, which was voxel-wise compared to corresponding mean scans from the healthy control sample (T = 3.93, p < 0.001 uncorrected).

PD 13 mean vs HC, PD>HC


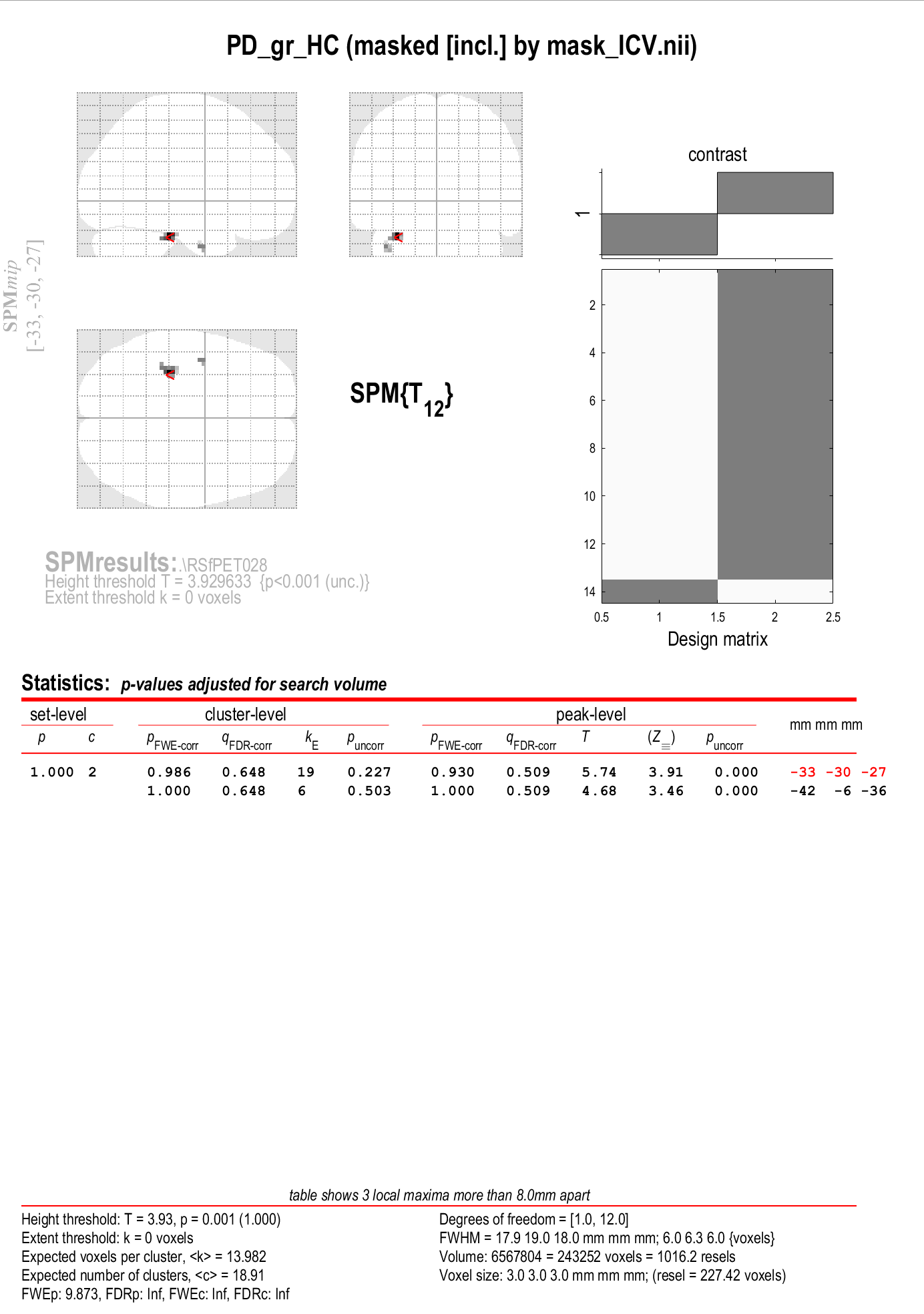


A


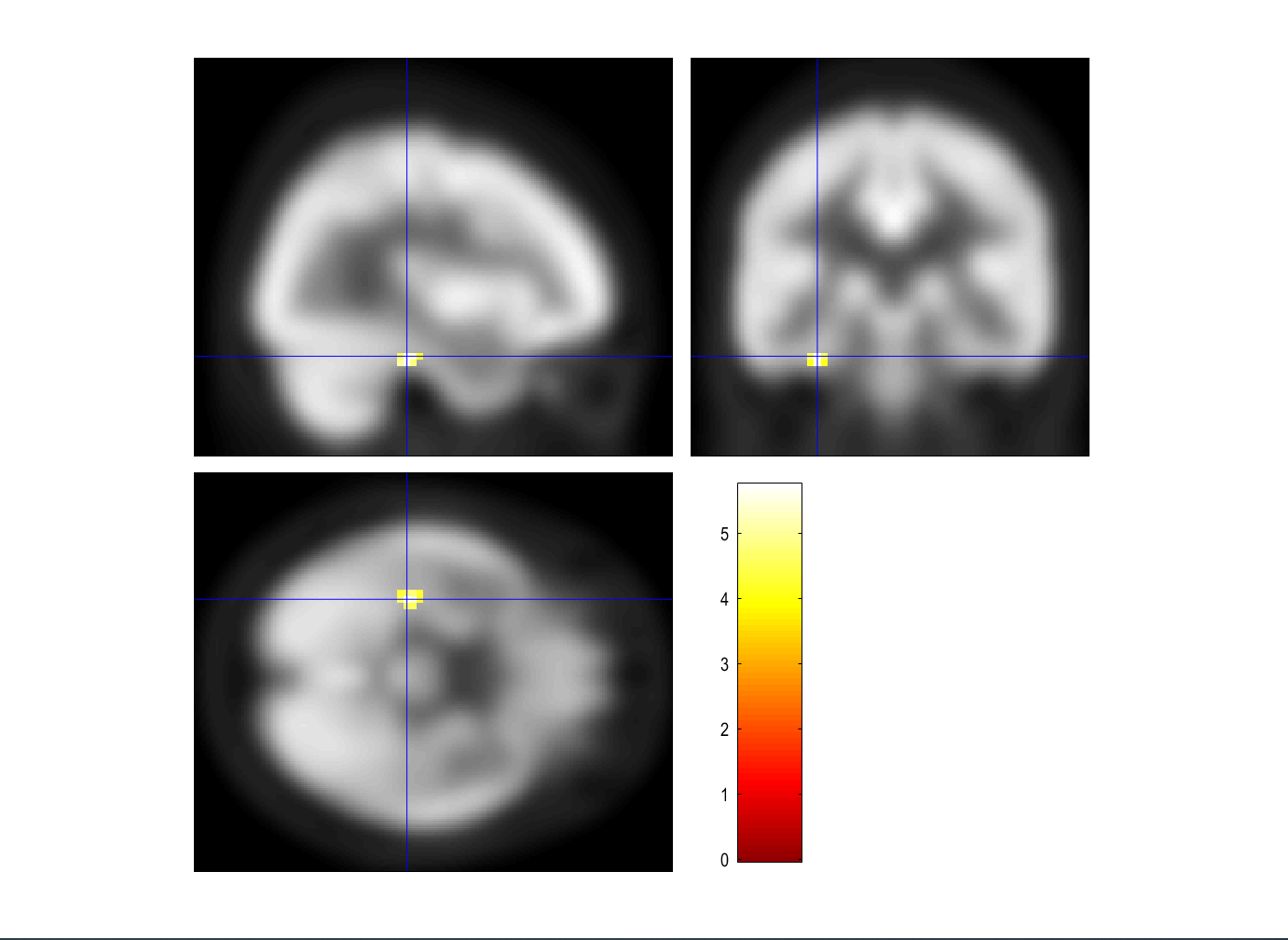


B

**Supplementary Fig. 22 Areas with relative hypermetabolism in patient 13 in comparison to healthy controls** Single subject SPM analysis of mean 18F-FDG-PET scan (min 61-90) from subject one, which was voxel-wise compared to corresponding mean scans from the healthy control sample (T = 3.93, p < 0.001 uncorrected).

PD13 mean vs HC, PD<HC


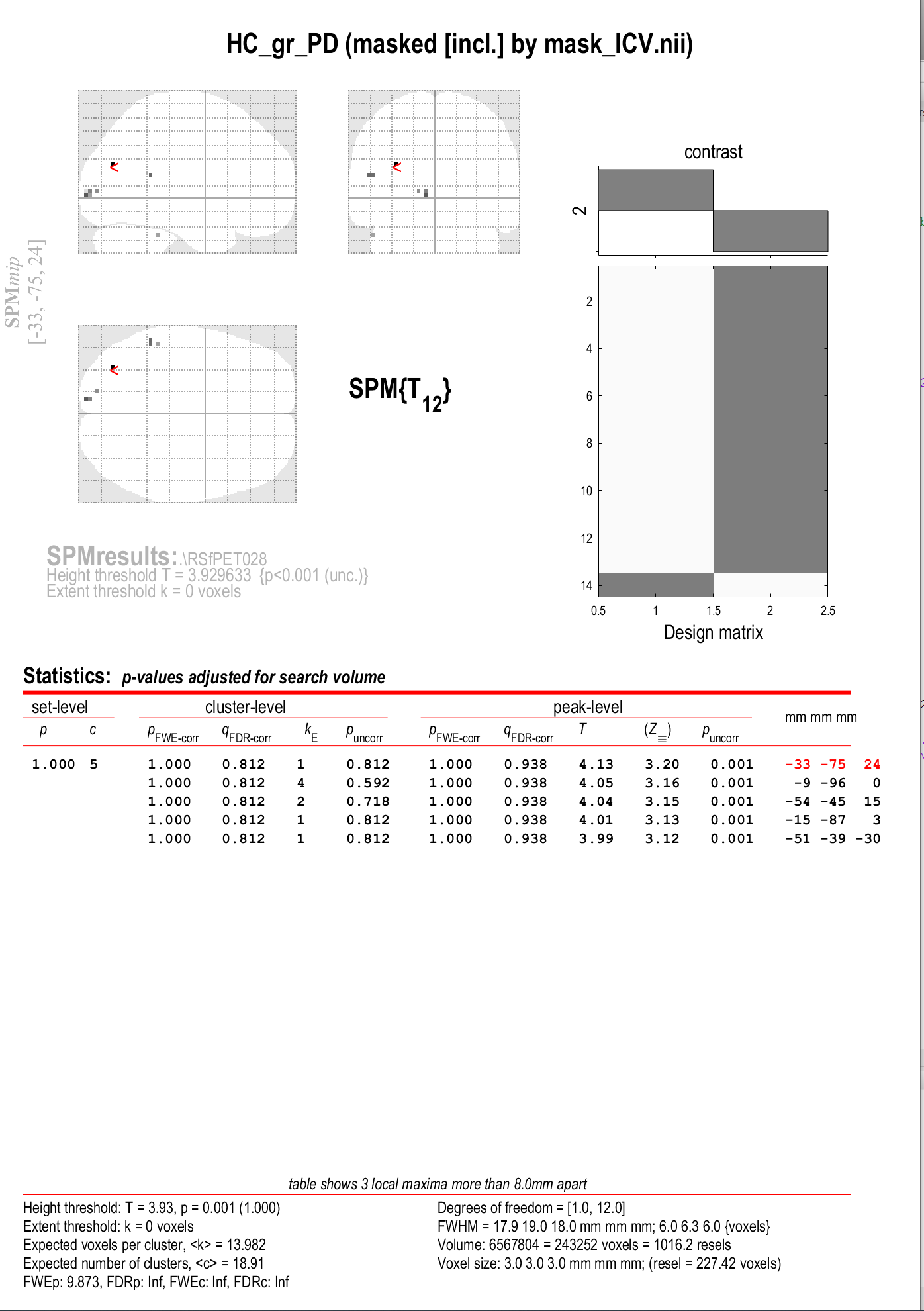


A


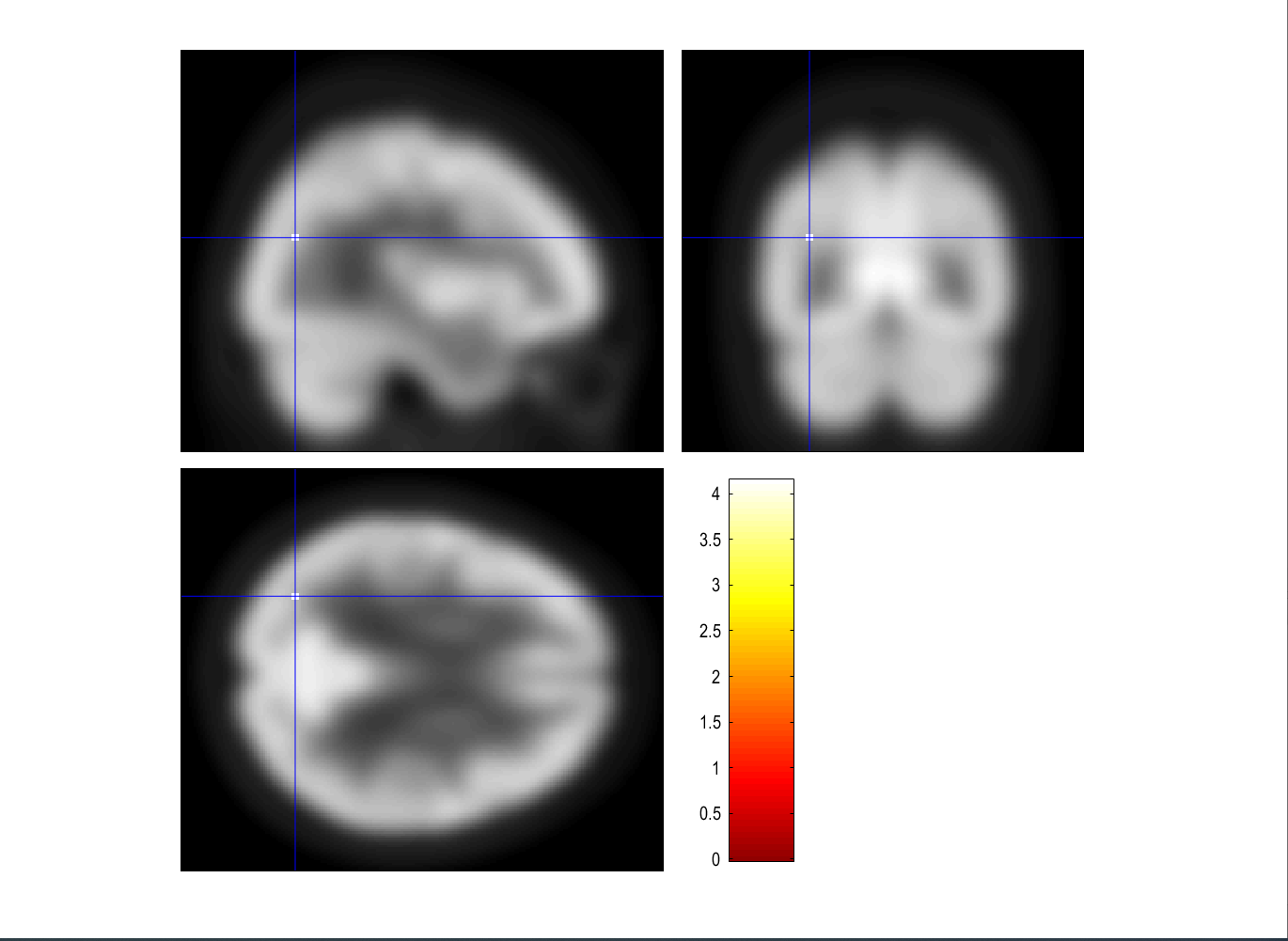


B

**Supplementary Fig. 23 Areas with relative hypometabolism in patient 13 in comparison to healthy controls** Single subject SPM analysis of mean 18F-FDG-PET scan (min 61-90) from subject one, which was voxel-wise compared to corresponding mean scans from the healthy control sample (T = 3.93, p < 0.001 uncorrected).

PD14 mean vs HC, PD>HC


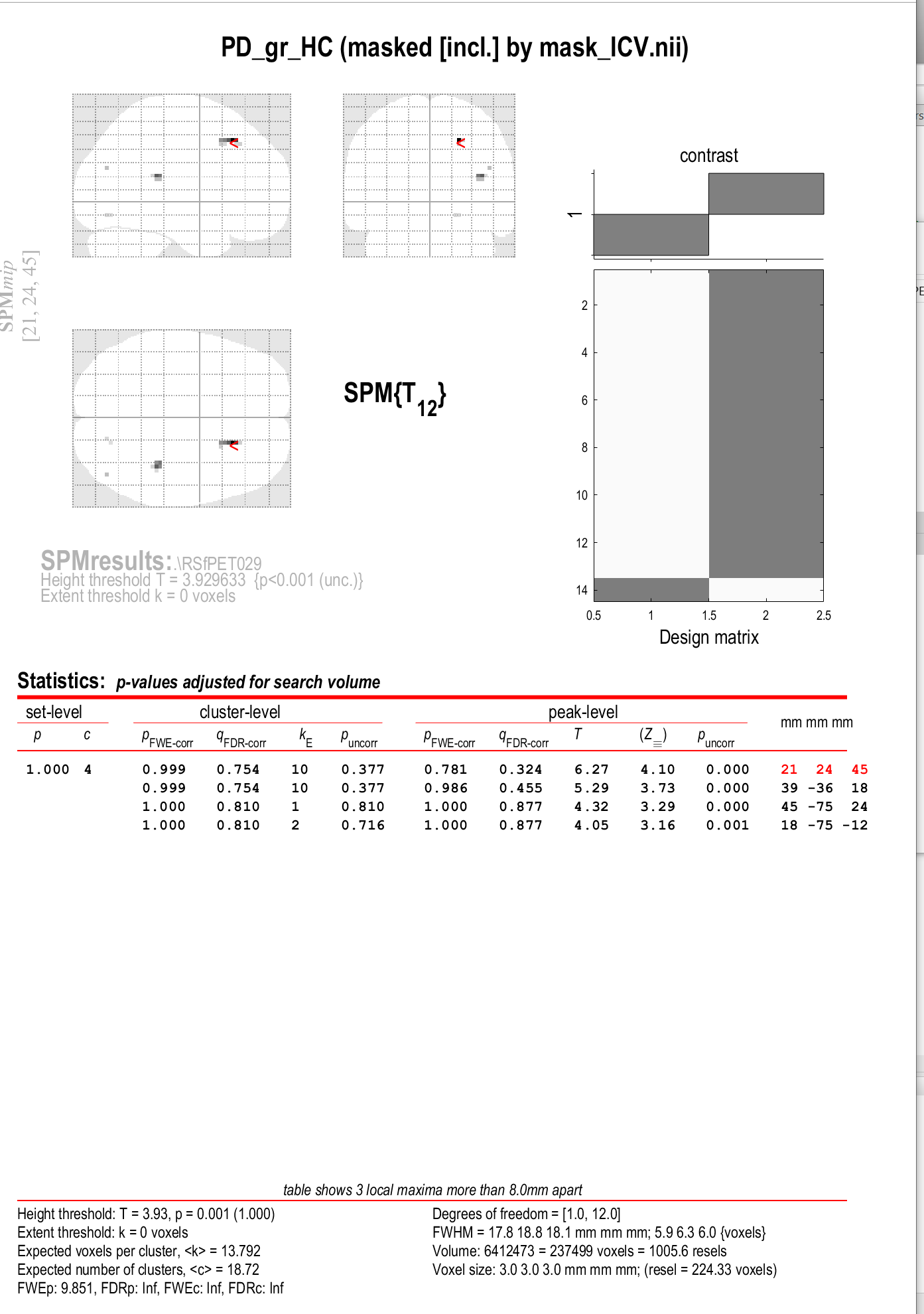


A


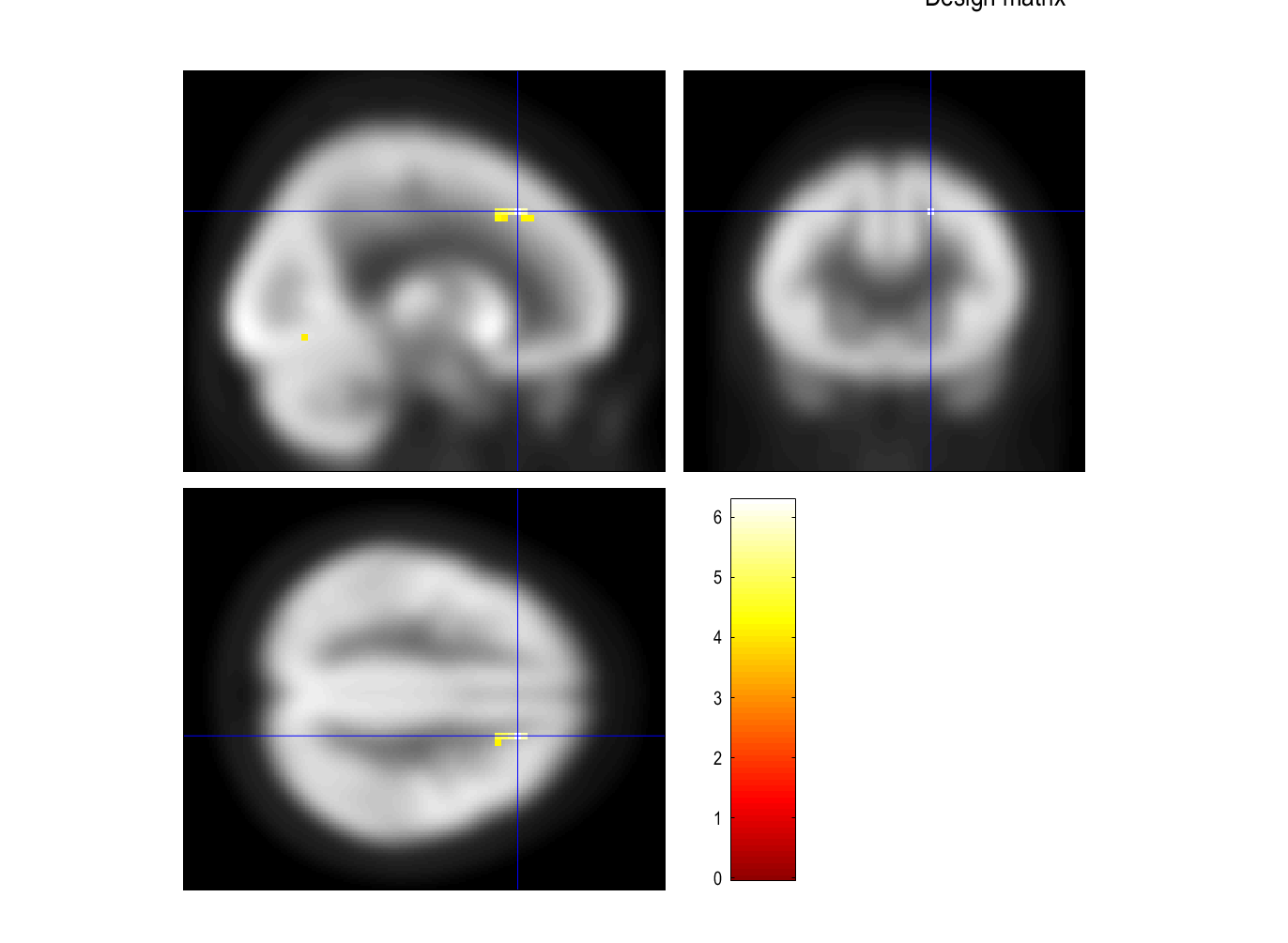


B

**Supplementary Fig. 24 Areas with relative hypermetabolism in patient 14 in comparison to healthy controls** Single subject SPM analysis of mean 18F-FDG-PET scan (min 61-90) from subject one, which was voxel-wise compared to corresponding mean scans from the healthy control sample (T = 3.93, p < 0.001 uncorrected).

PD 14 mean vs HC, PD<HC


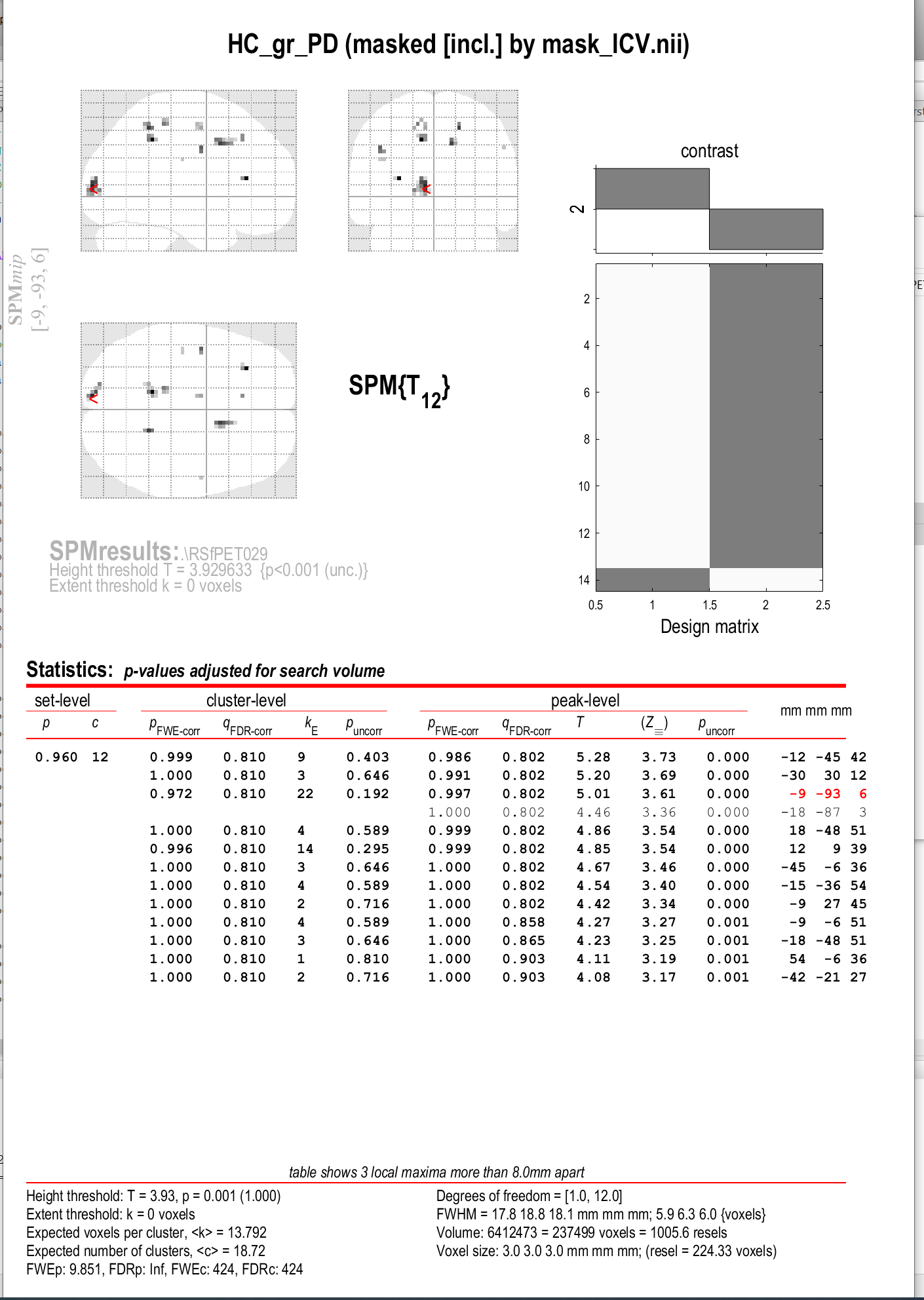


A


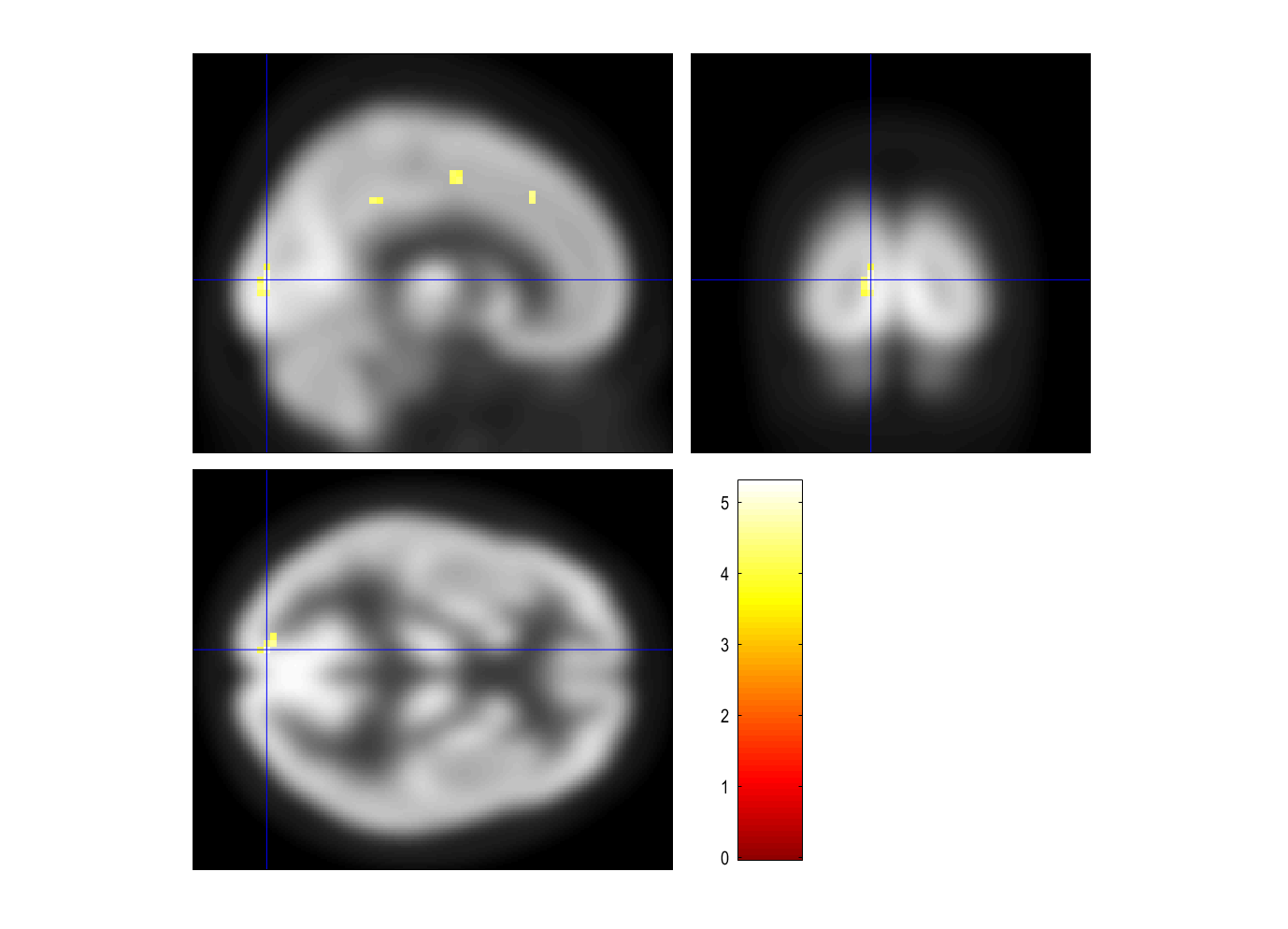


B

**Supplementary Fig. 25 Areas with relative hypometabolism in patient 14 in comparison to healthy controls** Single subject SPM analysis of mean 18F-FDG-PET scan (min 61-90) from subject one, which was voxel-wise compared to corresponding mean scans from the healthy control sample (T = 3.93, p < 0.001 uncorrected).


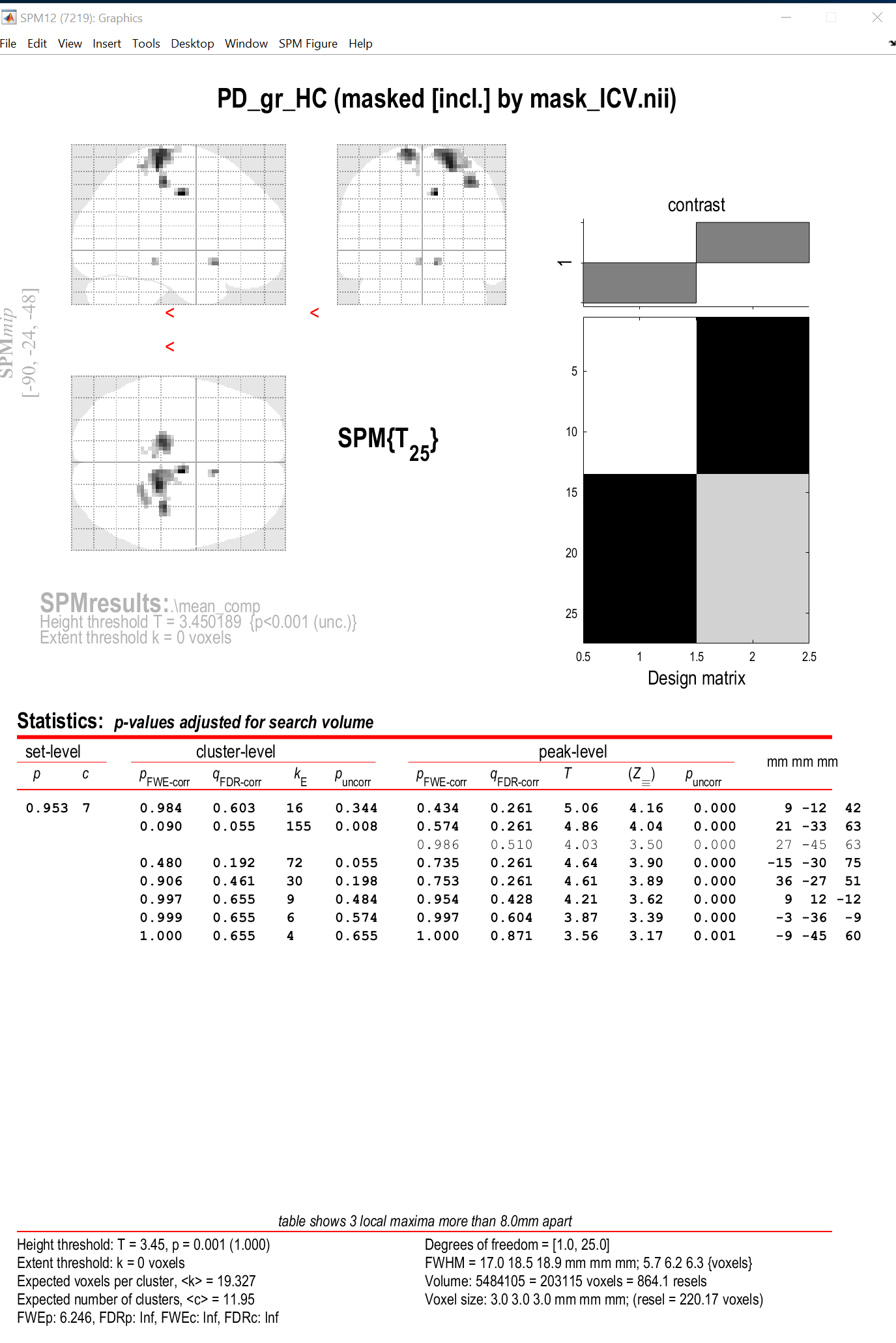


A


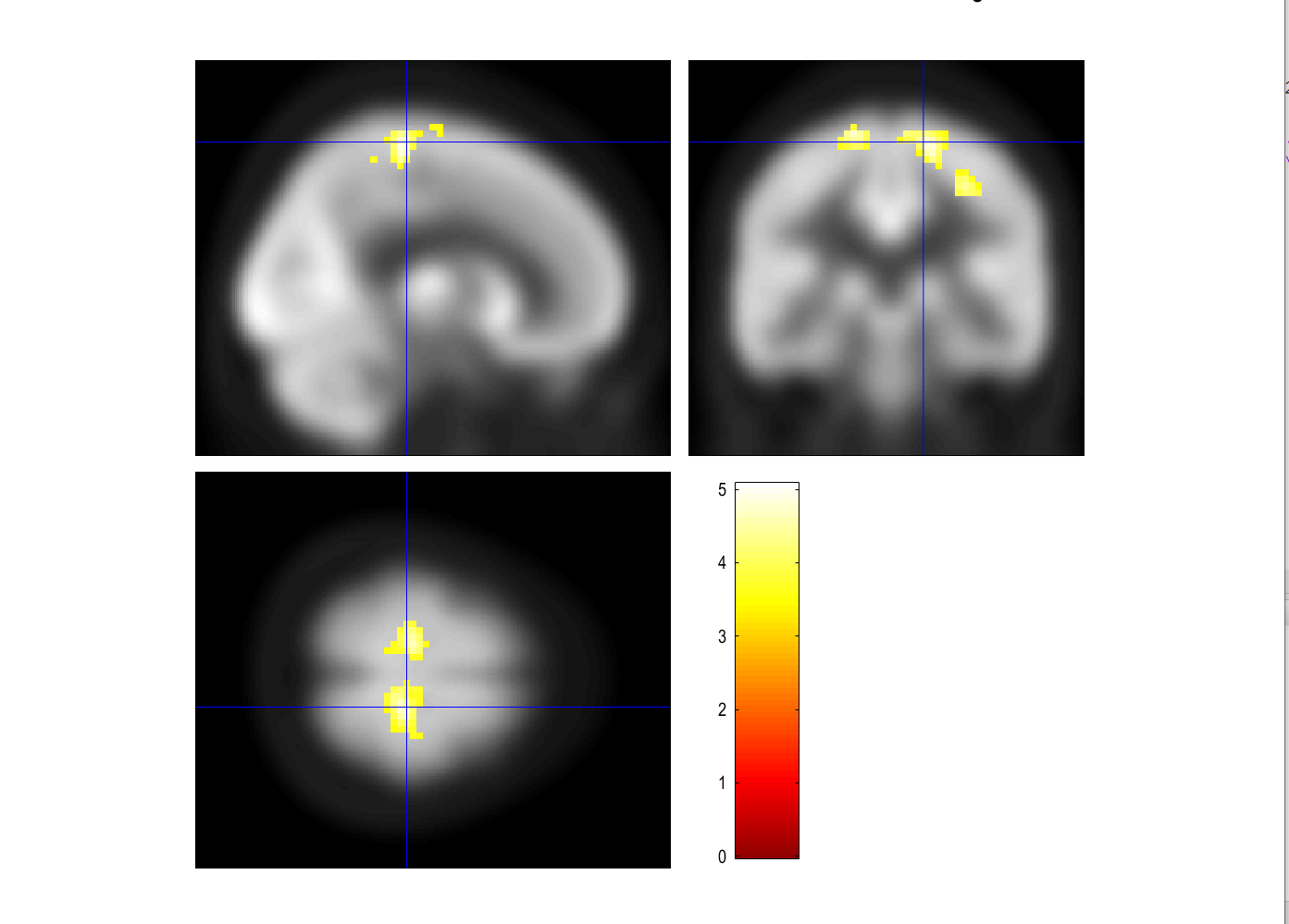


B

**Supplementary Fig. 26 Areas with relative hypermetabolism in patients in comparison to healthy controls** SPM group analysis of mean 18F-FDG-PET scans (min 61-90) from subject one, which was voxel-wise compared to corresponding mean scans from the healthy control sample (T = 3.45, p < 0.001 uncorrected).


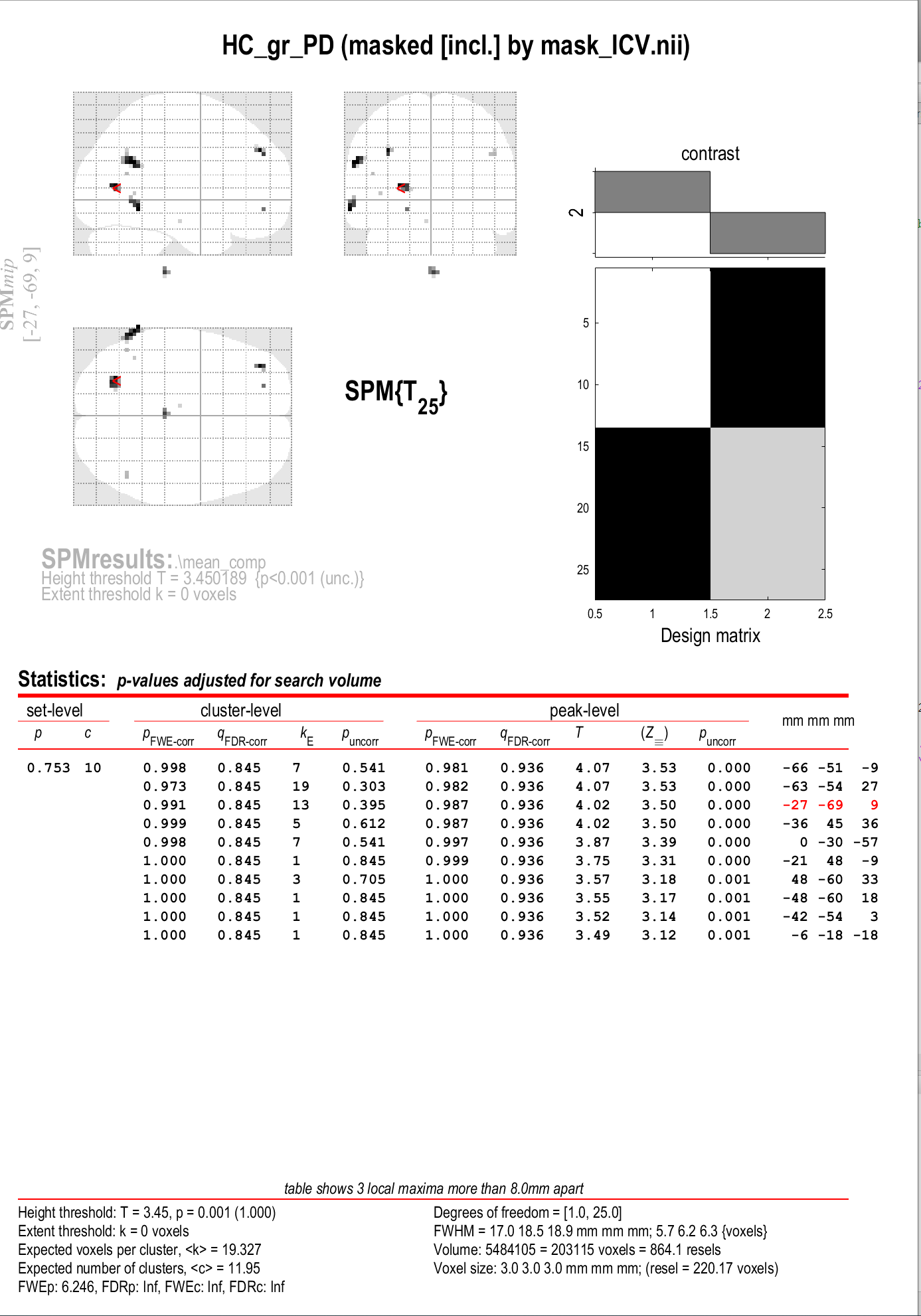


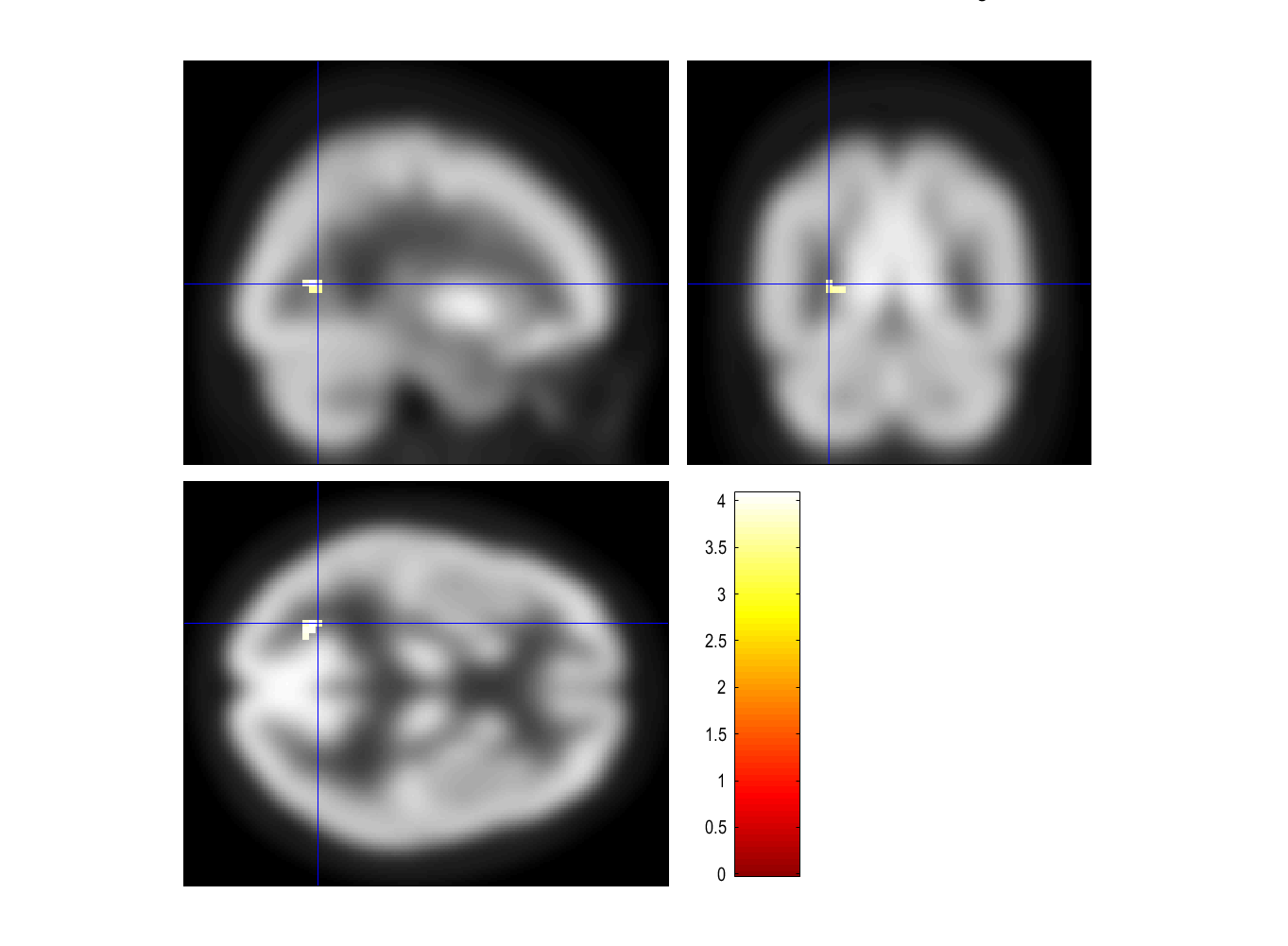


**Supplementary Fig. 27 Areas with relative hypometabolism in patients in comparison to healthy controls** SPM group analysis of mean 18F-FDG-PET scans (min 61-90) from subject one, which was voxel-wise compared to corresponding mean scans from the healthy control sample (T = 3.45, p < 0.001 uncorrected).
